# Dual relay Rh-/Pd-catalysis enables β-C(sp^3^)–H arylation of α-substituted amines†

**DOI:** 10.1039/d4sc06806h

**Published:** 2025-01-28

**Authors:** Shuailong Li, Sani Yahaya, Jan Bojanowski, Giulio Ragazzon, Paweł Dydio

**Affiliations:** a University of Cambridge Lensfield Road Cambridge CB2 1EW UK pd552@cam.ac.uk; b University of Strasbourg, CNRS ISIS UMR 7006, 8 Allée Gaspard Monge 67000 Strasbourg France

## Abstract

A dual relay catalytic protocol, built on reversible dehydrogenation of amines by Rh catalysis and C–H functionalisation of transient imines by Pd catalysis, is reported to enable regioselective arylation of amines at their unactivated β-C(sp^3^)–H bond. Notably, the new strategy is applicable to secondary anilines and N-PMP-protected primary aliphatic amines of intermediate steric demands, which is in contrast to the existing strategies that involve either free-amine-directed C–H activation for highly sterically hindered secondary aliphatic amines or steric-controlled migrative cross-coupling for unhindered *N*-Boc protected secondary aliphatic amines. Regioselectivity of the reaction is imposed by the electronic effects of transient imine intermediates rather than by the steric effects between specific starting materials and catalysts, thereby opening the uncharted scope of amines. In a broader sense, this study demonstrates new opportunities in dual relay catalysis involving hydrogen borrowing chemistry, previously explored in the functionalisation of alcohols, to execute otherwise challenging transformations for amines, commonly present in natural products, pharmaceuticals, biologically active molecules, and functional materials.

## Abstract 2

β-Arylation of secondary anilines and N-PMP-protected primary aliphatic amines was enabled by merging Rh-catalysed hydrogen borrowing and Pd-catalysed C–H functionalisation of transient imines.

## Graphical abstract

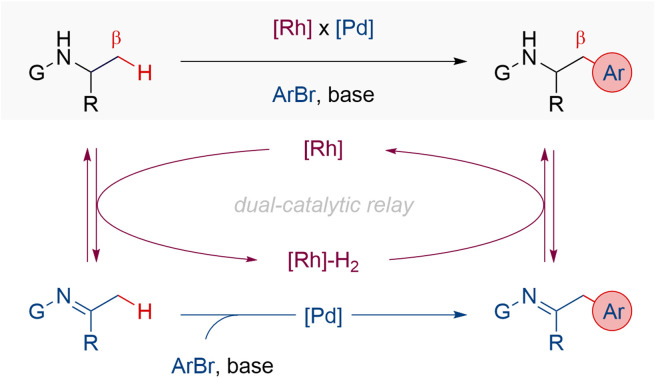

## Introduction

**Fig. 1:**
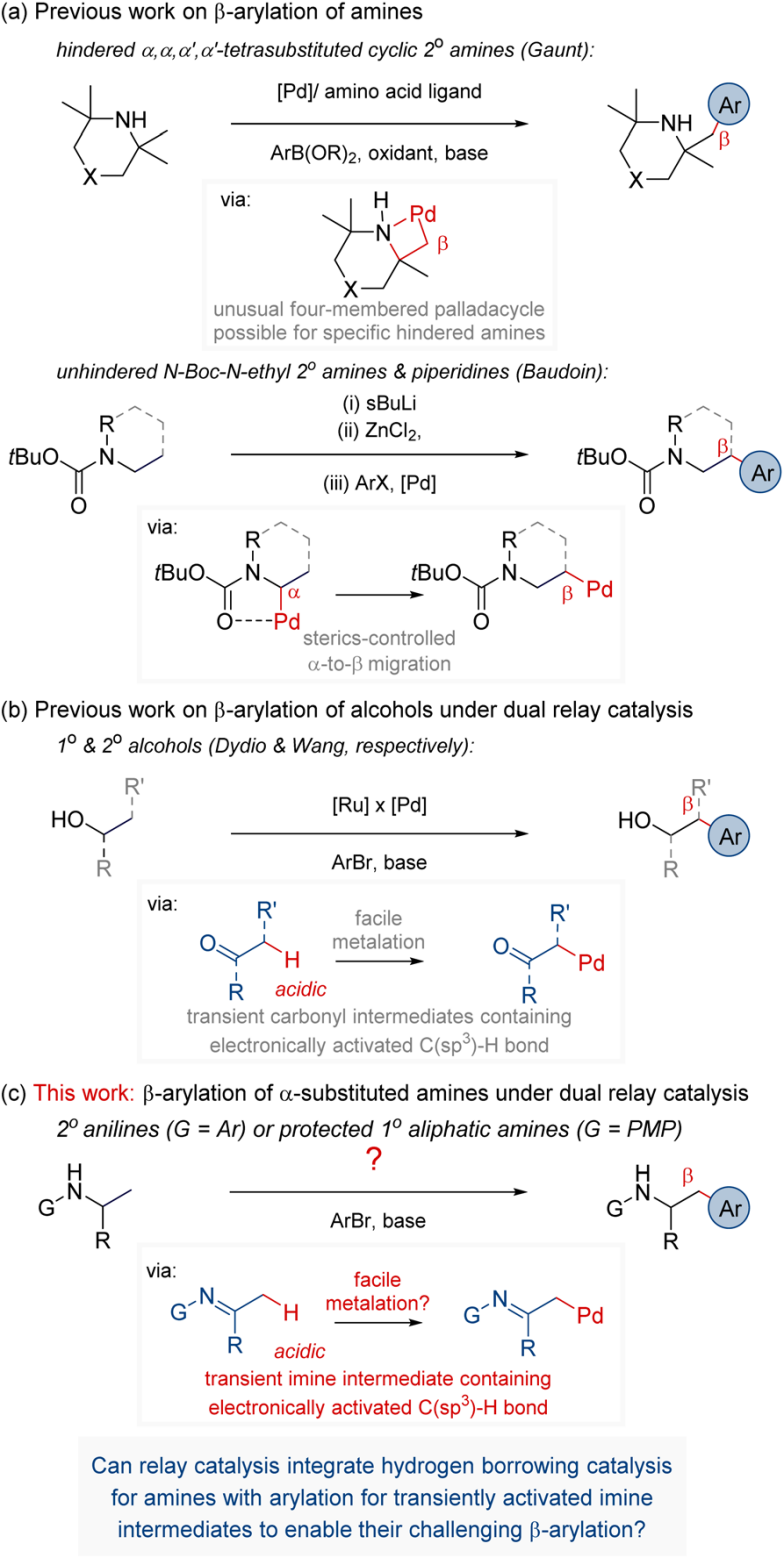
Context & the current work.

Given the widespread presence of aliphatic and aromatic amines, ranging from pharmaceuticals or agrochemicals to dyes or advanced materials, efficient methods for their synthesis and derivatisation are highly sought after.^1^ Various strategies were introduced to enable their site-selective C(sp^3^)–H functionalisation, each having its advantages and limitations concerning the scope, practicality, and generality.^2–9^ For instance, among the methods for the β-C(sp^3^)–H bond functionalisation, the strategy of ligand-enabled free-amine-directed C–H activation is particularly attractive, given that it does not require any additional steps to install or remove special directing groups and avoids any stoichiometric steps.^10,11^ Building on this strategy, Gaunt disclosed that free amines can undergo β-C(sp^3^)–H arylation catalysed by tailored Pd/amino-acid complex (Fig. 1a).^12^ However, the reaction occurs through a rare four-membered palladacycle^13,14^ and hence is applicable only to specific highly sterically hindered α,α,α′,α′-tetramethyl substituted secondary cyclic aliphatic amines. In turn, Baudoin reported that N-Boc-protected secondary aliphatic amines can undergo Pd-catalysed β-C(sp^3^)–H arylation through ligand-controlled migrative cross-coupling (Fig. 1a).^15–18^ However, the protocol is effective only for sterically unhindered alkyl chains of secondary aliphatic amines, such as *N*-ethyl amines and piperidines. In consequence, no reported strategies are effective for β-C(sp^3^)–H arylation of amines of intermediate steric demands, such as α-monosubstituted primary or secondary amines. Besides these, several studies on β-C(sp^3^)–H functionalisation reactions of amines other than β-C(sp^3^)–H arylation were also reported.^19–26^ Nevertheless, the toolkit of methods for selective β-C(sp^3^)–H functionalisation reactions remains underdeveloped when compared to myriads of α-, γ-, or δ-C(sp^3^)–H selective protocols.^27^ Thus, developing new strategies for β-C(sp^3^)–H functionalisation reactions of amines remains an important but challenging objective of chemical research.

Relay catalysis with multiple catalysts engaged in a sequence of independent reactions can unlock new transformations, including otherwise challenging C(sp^3^)–H bond functionalisation reactions.^28–41^ In that context, we^42,43^ and Wang^44^ reported protocols enabling selective β-C(sp^3^)–H bond arylation of primary and secondary alcohols under dual relay Ru-/Pd-catalysis (Fig. 1b). The reaction design capitalises on the reversible oxidation of alcohols by a Ru ‘hydrogen borrowing’ catalyst to form aldehyde/ketone intermediates. The transient carbonyl group temporarily activates the initially unactivated targeted C(sp^3^)–H bond, enabling its facile arylation by a Pd catalyst.

Bolstered by the capacity of dual relay catalysis to achieve β-C(sp^3^)–H arylation of alcohols, we sought a new strategy for β-C(sp^3^)–H arylation of amines (Fig. 1c). We considered that similar to alcohols, amines could undergo reversible oxidation under hydrogen borrowing catalysis^45–48^ to form transient imine intermediates.^49,50^ Noteworthy, imines have been extensively used as transient directing groups for C–H functionalisation of aldehydes, ketones, and primary amines.^51–57^ However, arylation of activated α-C(sp^3^)–H bonds of imines remains underdeveloped, in contrast to numerous protocols for Pd-catalysed arylation of activated α-C(sp^3^)–H bonds of aldehydes and ketones.^58^ Encouragingly, however, in the studies of domino synthesis of indoles, Barluenga and co-workers showed that a specific imine, *N*-phenyl-1-phenylethylamine, could undergo C–H arylation with 3-bromoanisole in the presence of a base and a Pd/phosphine complex.^59,60^ In addition, Seidel, Chen, and co-workers recently reported Pd-catalysed arylation of endocyclic 1-azaallyl anions of 2-aryl-1-piperidine, forming 2,3-diaryl-1-piperidine, which were subsequently reduced with NaBH_4_ to valuable *cis*-2,3-diarylpiperidines.^61^

## Results and discussion

**Scheme 1:**
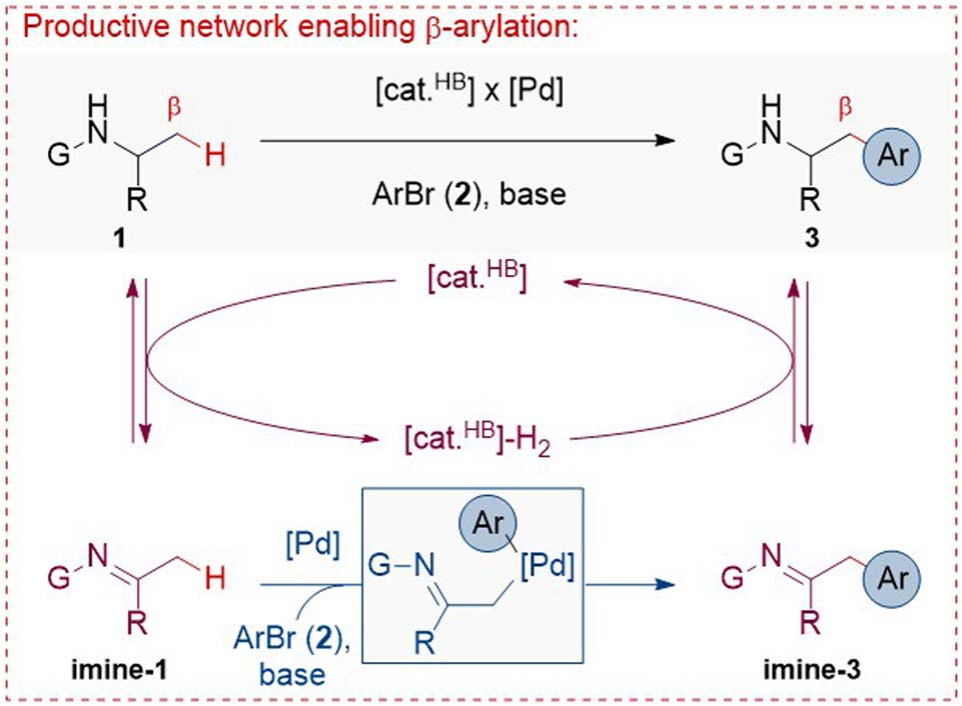
Design of β-arylation of amines in a dual-catalytic relay, building on reversible dehydrogenation of amines by [cat.HB] and arylation of transient imines by [Pd].

We considered that a meticulous combination of reversible dehydrogenation of amines by hydrogen-borrowing catalysis with arylation of transient imines by palladium catalysis could enable the unprecedented β-C(sp^3^)–H bond arylation of α-substituted amines (Scheme 1). Specifically, amine 1 is initially converted to imine-1 by a hydrogen borrowing catalyst, [cat.^HB^], which temporarily stores the ‘borrowed’ hydrogen, [cat.^HB^]-H_2_. Then, imine-1 reacts with aryl bromide 2 to form imine-3 in the presence of a base and a Pd catalyst, [Pd]. Imine-3 is eventually hydrogenated to product 3 by [cat.^HB^]-H_2_, consuming the initially ‘borrowed’ hydrogen and completing the overall process. The key challenges regard the compatibility of the catalytic species, the capacity of both processes to occur under the same conditions, and the ability to drive these processes toward the selective formation of the target product while inhibiting the formation of plausible side products.^29^ For instance, the other *N*-substituent (*i.e.*, group ‘G’ in Scheme 1) should not be susceptible to hydrogen borrowing, which would otherwise form a competitive imine intermediate, imposing site-selectivity issues. Furthermore, the initial amines should not undergo the classic Buchwald–Hartwig *N*-arylation under Pd catalysis,^62^ the arylated imine intermediates should not undergo secondary arylation processes,^59,60^ or imines should not undergo transamination processes, all of which would negatively impact the overall efficiency of the devised relay.

**Fig. 2:**
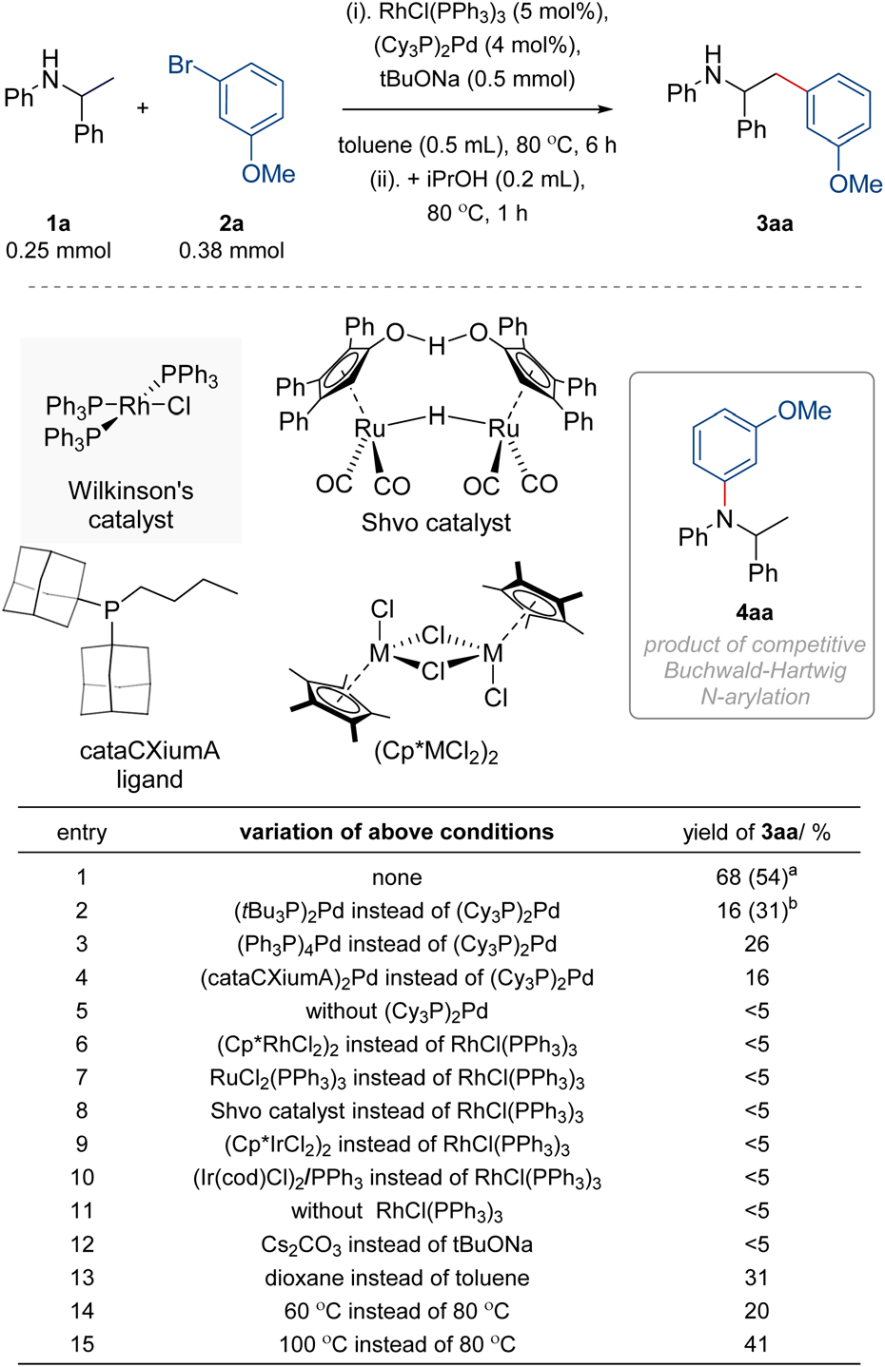
Reaction development – effect of different catalysts and conditions. Yields determined by ^1^H NMR analysis of reaction mixtures with an internal standard (1,3,5-trimethoxybenzene). ^a^Yield of isolated material reported in parentheses. ^b^Yield of 4aa with (*t*Bu_3_P)_2_Pd (<5% for other entries).

To investigate our design, we selected *N*-phenyl-1-phenylethylamine 1a as a model amine that, upon dehydrogenation, can form Barluenga's imine, which can react with 3-bromoanisole 2a and then be hydrogenated to target β-aryl amine 3aa (Fig. 2). During the initial exploration of a range of Ru, Ir, Rh, and Pd complexes, we observed that paring Rh with Pd catalysts enables the formation of target product 3aa under relatively mild conditions (80 °C, an alkoxide as a base) in contrast to pairing Ru or Ir with Pd complexes, which required more forcing conditions (>100 °C). However, the formation of 3aa was persistently accompanied by imine intermediates as the main side-products, irrespective of the reaction conditions and hydrogen borrowing complexes used. Fortunately, imines could be readily converted into amines upon the addition of isopropanol as a reductant, increasing the yield of target β-aryl amine 3aa.

With such approach, upon the condition optimisation, we found that the model reaction of 1a (0.25 mmol) and 2a (1.5 equiv.) formed 3aa in 68% NMR yield (54% isolated yield) along with 24% of unreacted 1a, when in the presence of Wilkinson's catalyst (RhCl(PPh_3_)_3_; 5 mol%), (Cy_3_P)_2_Pd (4 mol%), and sodium *tert*-butoxide (2 equiv.) in toluene (0.5 mL) at 80 °C for 6 h, followed by the addition of isopropanol (0.2 mL) and incubation at 80 °C for additional 1 h. Of note, attempts to increase the conversion of the remaining starting material by using more forcing conditions resulted in lower yields of 3aa due to different side processes. Fortunately, unreacted starting amines could be recovered when needed, increasing the effectiveness of the approach. Control experiments confirmed the importance of each element of the reaction conditions, as summarised in Fig. 2. For instance, the replacement of (Cy_3_P)_2_Pd with (*t*Bu_3_P)_2_Pd resulted in the formation of undesired Buchwald–Hartwig *N*-arylated amine 4aa as the main product (31% yield) along with minor targeted 3aa (16%) and unreacted 1a (25%). Likewise, the use of Ru- or Ir-based complexes previously used in hydrogen autotransfer reactions for amines^46,48,63–65^ in place of Wilkinson's catalyst was ineffective under the same conditions. Noteworthy, we found that the yield of the product is sensitive to the ratio of Pd/Rh complexes (Fig. S1†), with higher amounts of Pd resulting in lower yields due to competitive side processes. Given the limited stability of (Cy_3_P)_2_Pd (*e.g.*, ∼50% decomposition upon storage at 22 °C for 1 week in a nitrogen-filled glovebox, Fig. S2†), and its variable purity from commercial suppliers, the most reproducible results were obtained when (Cy_3_P)_2_Pd was prepared in house^66^ and stored carefully (at −40 °C in a glovebox; <5% decomposition after 3 months). Attractively, the use of high-purity (Cy_3_P)_2_Pd permits lowering its loading to just 1 mol% to form the product in 65% yield (Fig. S1†).^67^

**Fig. 3:**
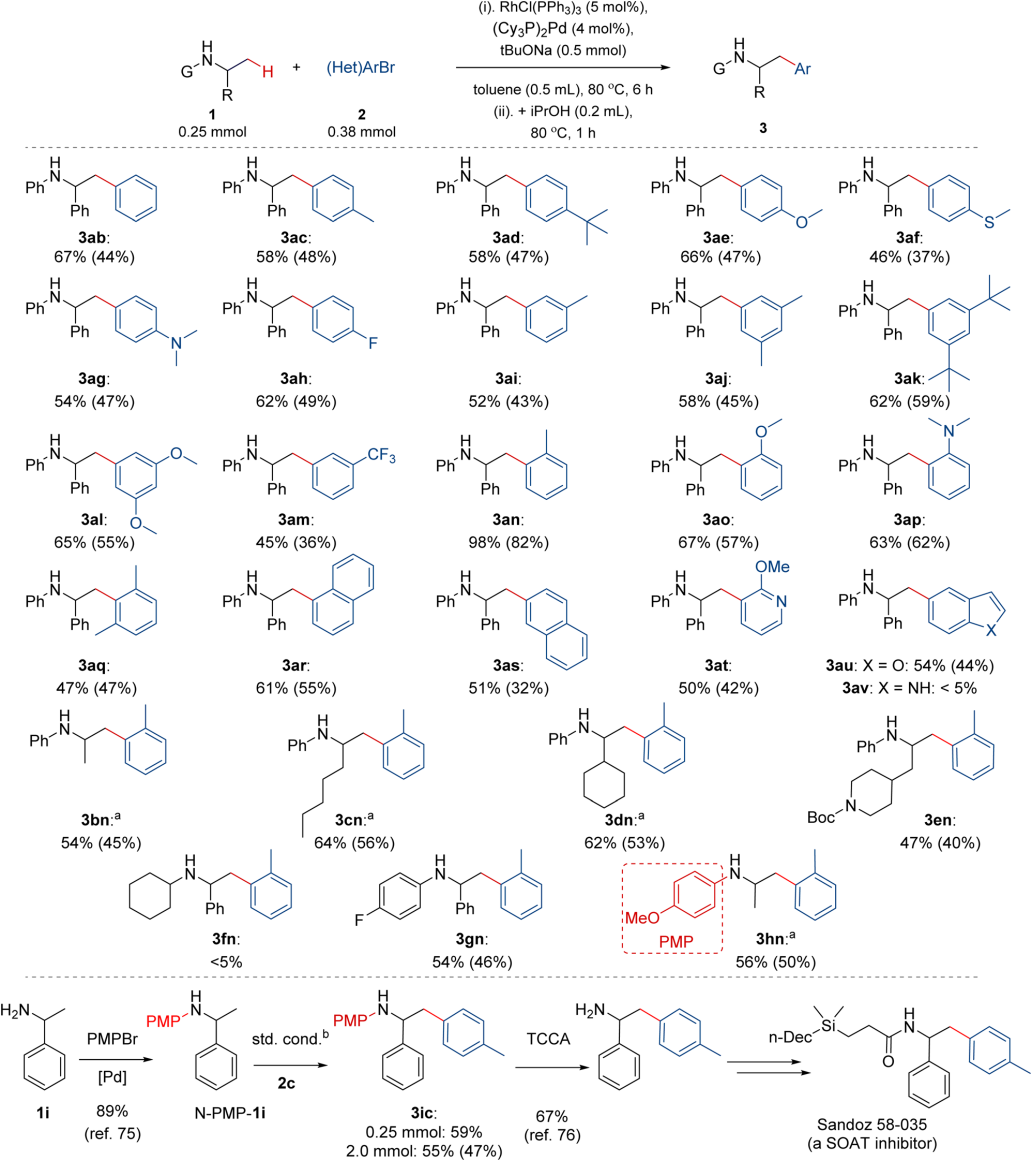
Scope of (hetero)aryl bromides and α-substituted amines. Both spectroscopic and isolated yields (in parenthesis) are reported; yields of isolated material are in some cases substantially lower than spectroscopic yields due to the difficulty of separating target products from remaining starting materials. ^a^2 mol% Pd, 10 mol% Rh. ^b^Standard conditions reported in Fig. 3, top.

With the optimized conditions for the model reaction of amine 1a with aryl bromide 2a, we next investigated the performance of this relay catalytic system with varied starting materials (Fig. 3). We found that a broad range of electronically-varied aryl bromides 2b–2r bearing different functional groups of varied sizes reacted effectively with amine 1a, forming β-aryl amines 3ab–3ar in 45–98% yields. Reactions with highly electron-deficient aryl bromides, such as 3,5-bis(trifluoromethyl)bromobenzene were low yielding, illustrating the current limitation of the method. Notably, different substitution patterns of aryl bromides were well tolerated, including *ortho*-, *meta*-, *para*-substituted, and most congested 2,6-disubstituted derivatives, such as 3aq. Further, polycyclic aryl bromides, such as naphthyls 2r–2s, proved competent substrates in these reactions, forming 3ar–3as in 51–61% yields. Finally, the reactions of 1a with heteroaryl bromides, such as pyridine 2t and benzofuran 2u, produced target 3at–3au in 50–54% yields, while the reaction with indole 2v formed trace amounts of the product.

We found that a modest but distinct range of structurally varied amines was suitable for the C–H arylation. Specifically, α-substituent could be varied from phenyl to methyl, *n*-pentyl, cyclohexyl, and *N*-Boc-piperidyl of different steric and electronic demands, as indicated by the formation of target *N*-phenyl α-substituted-β-arylethylamines 3bn–3en in 47–64% yields. We found that the *N*-aryl substituent is relevant. *N*-cyclohexyl amine 1f did not react to form target 3fn, instead undergoing decomposition, suggesting side-process occurring for the corresponding imine intermediates. Gratifyingly, the *N*-substituent can be an electron-rich or electron-deficient aryl ring (3gn–3hn, 54–56% yields), including the *N*-(*p*-methoxyphenyl) group (*N*-PMP), which can be readily installed^62,68^ and removed^69–71^ from primary amines. Attractively, *N*-PMP installation, β-(Csp^3^)–H arylation, and deprotection creates a new pathway from simple primary amines to α-alkyl-β-arylethylamines and α,β-bisarylethylamines, important classes of bio-active compounds, including neuro-pharmaceuticals, such as amphetamine, tenamfetamine, and norfenfluramine,^72,73^ and estrogen receptor inhibitors, such as Sandoz 58-035, relevant in pathologies, including atherosclerosis, Alzheimer's disease, and cancer.^74^ Exploiting the previously reported protocols for the installation^75^ and removal^76^ of *N*-PMP group, the α,β-bisarylethylamine scaffold of Sandoz 58-035 can be prepared thanks to the developed protocols (Fig. 3, bottom). Corresponding amine N-PMP-1i underwent β-arylation in 59% yield on 0.25 mmol scale and 55% yield (47% isolated yield) on 2 mmol scale, delivering over 300 mg of the material and showcasing the scalability of the reaction. Noteworthy, the remaining mass balance constitutes mostly unreacted amine 1i (42%), which could be recovered.

**Fig. 4:**
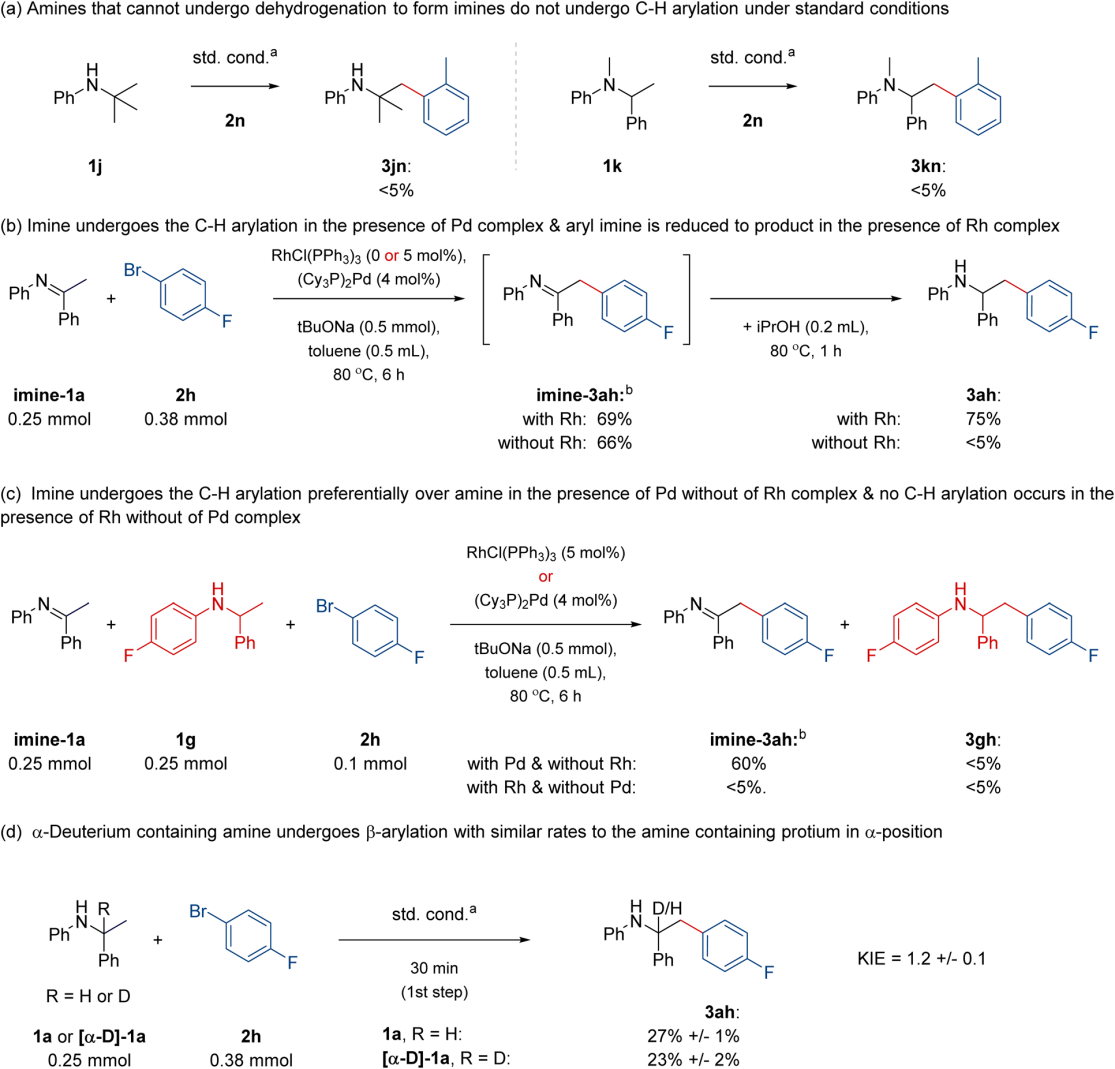
Control mechanistic experiments. ^a^Standard conditions reported in Fig. 3. ^b^Yield of imine-3ah was measured upon HCl-mediated hydrolysis to a ketone derivative; for details, see the ESI.†

Finally, we performed control mechanistic experiments that corroborate the proposed dual relay mechanism connecting the dehydrogenation of amines by Rh catalysis with C–H functionalisation of transient imines by Pd catalysis. First, amines 1j–1k that cannot undergo dehydrogenation to form imines did not form the C–H arylation products under the devised standard reaction conditions (Fig. 4a). Second, imine-1a reacted with 2h to form imine-3ah in 66–69% yield, under standard conditions, with or without the hydrogen-borrowing Wilkinson's catalyst present (Fig. 4b). In the experiment with Wilkinson's catalyst present, upon the addition of isopropanol, imine-3ah was eventually converted into amine 3ah in 75% overall yield (Fig. 4b). Third, the competition experiment between imine-1a and amine 1g reacting with 2h under the standard conditions in the presence of Pd complex without the hydrogen-borrowing Wilkinson's catalyst resulted in the selective arylation of imine-1a over amine 1g (Fig. 4c). In turn, the same experiment in the presence of Wilkinson's catalyst but without the Pd complex resulted in no arylation products (Fig. 4c). Lastly, deuterium-containing amine [α-D]-1a reacted with 2h in similar initial rates to nondeuterated amine 1a, suggesting that the dehydrogenation of the α-C–H bond is not the rate-limiting step in this dual catalytic process (Fig. 4d). Overall, these data are in line with the catalytic relay involving the initial Rh-catalysed formation of an imine intermediate, which undergoes the Pd-catalyzed reaction with an aryl bromide and is eventually hydrogenated by the Rh complex to the amine product (*cf.*, Scheme 1). In turn, they are inconsistent with the C–H arylation of an amine involving the direct C–H bond activation of the starting material.

## Conclusions

To summarise, the work presented here introduces dual relay catalysis with hydrogen borrowing chemistry to execute functionalisation for amines, exemplified by conducting otherwise challenging regioselective arylation of unactivated β-C(sp^3^)–H bonds of α-substituted secondary anilines or N-PMP protected primary aliphatic amines. Given different methods for the functionalisation of alcohols enabled by relay catalysis with hydrogen borrowing chemistry^42–44,77–100^ we expect our study will stimulate the research enabling other previously inaccessible transformations for amines.

## Data availability

All synthetic procedures, characterization data, spectroscopic data, supplementary figures and tables, and detailed information can be found in the ESI.†

## Author contributions

P. Dydio conceived and supervised the project. S. Li, J. Bojanowski and S. Yahaya carried out the experimental works. S. Li and S. Yahaya collected, solved, and refined all the data. P. Dydio wrote the manuscript with the feedback from all authors. All authors have given approval to the final version of the manuscript.

## Conflicts of interest

There are no conflicts to declare.

## Supplementary Material

SC-OLF-D4SC06806H-s001
