# Supplementary material for: Dual relay Rh-/Pd-catalysis enables β-C(sp3)–H arylation of α-substituted amines
Source: Chem Sci. 2025 Jan 28;16(9):4167–74. doi: 10.1039/d4sc06806h (PMC11791518; doi:10.1039/d4sc06806h)
Supplement: SC-016-D4SC06806H-s001 [file SC-016-D4SC06806H-s001.pdf]

Supplementary Information (SI) for Chemical Science.
This journal is © The Royal Society of Chemistry 2025

Dual Relay Rh-/Pd-Catalysis Enables β-C(sp^3)-H Arylation of α-Substituted Amines

Shuailong Li,[a,b,†] Sani Yahaya,[b†] Jan Bojanowski,[b] Giulio Ragazzon[b] and Paweł Dydio[*a,b]

[a] *University of Cambridge, Lensfield Road, Cambridge, CB2 1EW, UK.*
[b] *University of Strasbourg, CNRS, ISIS UMR 7006, 8 Allée Gaspard Monge, 67000 Strasbourg, France.*
[†] *These authors contributed equally to this work as the first author*
* *Correspondence to:* pd552@cam.ac.uk

Contents

1. General remarks 2
2. Preparation of catalyst $(Cy_3P)_2Pd$ 3
3. Preparation of starting materials 4
4. The effect of the ratio between $(Cy_3P)_2Pd$ /$RhCl(PPh_3)_3$ complexes & the influence of the $(Cy_3P)_2Pd$ quality 8
5. ^{31}P NMR spectra of prepared and commercial $(Cy_3P)_2Pd$ 10
6. General procedure for the preparation of β-arylamines 12
7. Scale-up reaction 24
8. Control mechanistic experiments 25
9. NMR spectra 29
10. References 70

1. General remarks

Unless stated otherwise, all reactions and manipulations were conducted on the laboratory bench or in a well-ventilated fume hood in air with reagent-grade solvents. Reactions under an inert gas atmosphere were set up in a nitrogen-filled glove box or by standard Schlenk techniques under nitrogen. Unless noted otherwise, all reagents and solvents were purchased from commercial suppliers and used without further purification. For experiments under an inert gas atmosphere, dried and degassed solvents were purchased from commercial suppliers, stored in a nitrogen-filled glove box and used as received. Column chromatography was carried out with the aid of a CombiFlash EZ Prep Chromatography System with integrated ELSD using the RediSep Rf (Gold) Silica Gel Disposable Flash columns or performed manually using Merck Kieselgel 60 (230-400 mesh). Analytical thin layer chromatography (TLC) was carried out on Merck Kieselgel F254 plates. TLC visualisation was carried out with ultraviolet light (254 nm), followed by staining with a 1% aqueous $KMnO_4$ solution. Preparative TLC was carried out on Sigma-Aldrich PLC Silica gel 60 F_{254}, 1 mm plates. NMR spectra were acquired on Bruker spectrometers at the facilities of the Yusuf Hamied Department of Chemistry, University of Cambridge, or the Institute of Science and Supramolecular Engineering, the University of Strasbourg and CNRS. NMR spectra were processed using the MestReNova software. Chemical shifts are reported in parts per million (ppm) downfield from an internal standard $CDCl_3$ (7.26 ppm for ^{1}H) and (77.16 ppm for ^{13}C), respectively. Coupling constants (*J*) are reported in hertz (Hz). NMR yields were calculated using 1,3,5-trimethoxybenzene as an internal standard. Electrospray-ionisation quadrupole-time-of-flight high-resolution mass spectrometric (ESI-QTOFHRMS) experiments were performed with a Synapt G3-S HDMS, Waters Co., Milfod, MA, USA, at the Yusuf Hamied Department of Chemistry, University of Cambridge. Electrospray ionisation high-resolution mass spectra (ESI-HRMS) were recorded on a ThermoFisher Ultimate 3000 instrument with a Scientific Vanquish Flex UHPLC instrument with a ThermoFisher Orbitrap: Exactive Plus with Extend Mass Range: Source HESI II (at ISIS).

2. Preparation of catalyst $(Cy_3P)_2Pd$

The $(Cy_3P)_2Pd$ was prepared according to the literature procedure.[1]

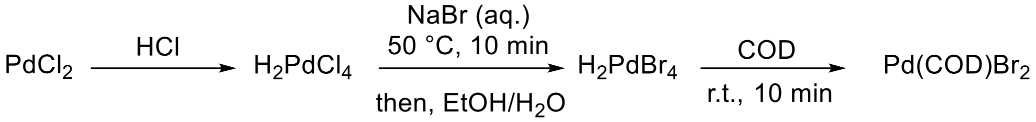

In a 100 mL flask, $PdCl_2$ (2.0 g) was treated with concentrated HCl (5 mL) and stirred at room temperature for 10 min. NaBr (4.65 g) in water (7 mL) was added and the mixture was allowed to stir at 50 °C for 10 min. After cooling down, the solution was filtered and the residue was washed with a mixture of ethanol/water (v/v = 3/1, 30 mL). To the filtrate 1,5-cyclooctadiene (3 mL) was added, and the mixture was slowly stirred at room temperature for 5 min while bright orange precipitate was produced. The suspension was settled for 10 min. The solid material was collected by filtration, washed with water (50 mL) and diethyl ether (50 mL) to give the $Pd(COD)Br_2$ as an orange solid (3.0 g, 90% yield).

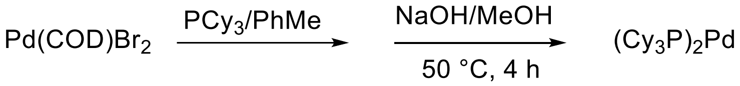

All subsequent manipulations were performed inside of a nitrogen-filled glovebox. A 20 mL vial was charged with $Pd(COD)Br_2$ (400 mg), followed by addition of toluene (2 mL), and the resulting suspension was allowed to stir at room temperature for 10 min. (Then cooling the solution in a freezer after this step is helpful for obtaining a higher yield). Then, NaOH (4.5 M in methanol, 1.3 mL) was added, which results in the formation of a white precipitate. A solution of PCy_3 (600 mg) in toluene (3 mL) was added to the mixture and allowed to stir at 50 °C for 4 h. After cooling to 22 °C, the supernatant liquid was transferred into a 100 mL flask, methanol (30 mL) was added and the mixture was stirred at room temperature for another 1 h (grey precipitate was formed). The suspension was filtered. The collected solid material was washed with methanol (3 x 5 mL). The obtained grey solid (600 mg, 66% yield) was dried under vacuum and stored in a glovebox in a freezer (-40 °C).

^{1}H NMR (500 MHz, C_6D_6) δ 2.27 – 2.16 (m, 12H), 1.87 – 1.77 (m, 18H), 1.74 - 1.62 (m, 18H), 1.33 – 1.21 (m, 18H).

^{31}P NMR (203 MHz, C_6D_6) δ 39. 1.

3. Preparation of starting materials

Method A: Substrates **1a**, **1g** were prepared according to the literature procedure.[2]

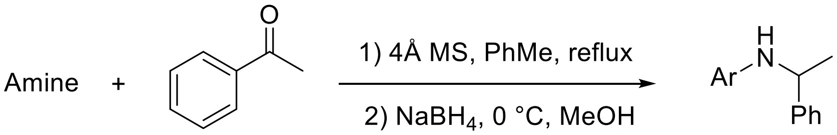

To a solution of corresponding aryl amine (10 mmol, 1 equiv.) and corresponding ketone (1 equiv.) in toluene (20 mL), activated 4Å MS (5 g) was added. The resulting mixture was allowed to reflux overnight under dry atmosphere (the reflux condenser was secured with a tube containing $CaCl_2$ as drying agent). The reaction mixture was cooled to room temperature and filtered through a plug of celite, which was then washed with copious amounts of methanol. The volatiles from the filtrate were evaporated under reduced pressure. (The imine intermediate can be recrystalized from hexane at -40 °C). To the residue, methanol (10 mL) was added. The mixture was cooled to 0 °C (an ice bath). $NaBH_4$ (0.8 g, 20 mmol, 2 equiv.) was added portion-wise. The reaction mixture was allowed to warm to room temperature and stir overnight. The reaction mixture was quenched with aqueous 1M NaOH followed by water. Excess diethyl ether was added to achieve separation of phases. The organic layer was collected, washed with brine, dried over anhydrous Na_2SO_4, and filtered. The volatiles from the filtrate were removed under reduced pressure. The reside was subjected to column chromatography (dry load with celite, 120 g silica, typically gradient from 0-20% diethyl ether in petroleum ether) to isolate the pure product.

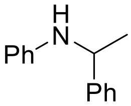

***N-(1-phenylethyl)aniline* 1a.** Following the above procedure, the title compound was obtained as pale-yellow oil using aniline (0.93 g, 10 mmol) and acetophenone (1.2 g, 10 mmol) and purified with silica-gel column chromatography, eluting with 10% diethyl ether in petroleum ether (containing 0.2% triethylamine) (60%, 1.2 g).[2b]

^{1}H NMR (400 MHz, $CDCl_3$) δ 7.42 – 7.36 (m, 2H), 7.36 – 7.29 (m, 2H), 7.28 – 7.20 (m, 1H), 7.16 – 7.06 (m, 2H), 6.69 (tt, *J* = 7.3, 1.1 Hz, 1H), 6.60 – 6.53 (m, 2H), 4.50 (q, *J* = 6.7 Hz, 1H), 4.03 (brs, 1H), 1.55 (d, *J* = 6.8 Hz, 3H).

^{13}C NMR (101 MHz, $CDCl_3$) δ 147.3, 145.3, 129.2, 128.8, 127, 126, 117.5, 113.5, 53.7, 25.1.

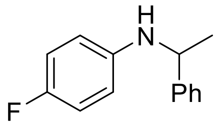

***4-fluoro-N-(1-phenylethyl)aniline* 1g.** Following the above procedure, the title compound was obtained as pale-yellow oil using 4-fluoroaniline (0.55 g, 5 mmol) and acetophenone (0.6 g, 5 mmol) and purified with silica-gel column chromatography, eluting with 10% diethyl ether in petroleum ether (containing 0.2% triethylamine) (56%, 0.6 g).

^{1}H NMR (500 MHz, CDCl$_3$) δ 7.39 – 7.28 (m, 4H), 7.28 – 7.19 (m, 1H), 6.86 – 6.75 (m, 2H), 6.51 – 6.38 (m, 2H), 4.42 (q, *J* = 6.7 Hz, 1H), 3.92 (brs, 1H), 1.51 (d, *J* = 6.7 Hz, 3H).

^{13}C NMR (101 MHz, CDCl$_3$) δ 155.8 (d, *J* = 234.9 Hz), 145.2, 143.8, 128.8, 127.1, 126.0, 115.6 (d, *J* = 22.2 Hz), 114.2 (d, *J* = 7.4 Hz), 54.2, 25.3.

^{19}F NMR (471 MHz, CDCl$_3$) δ -128.3.

HRMS (ESI, m/z): calculated for $C_{14}H_{15}FN$ $[M+H]^+$: 216.1189, found: 216.1199.

Method B: Substrates **1c, 1d, 1h** were prepared according to the literature procedure.[3]

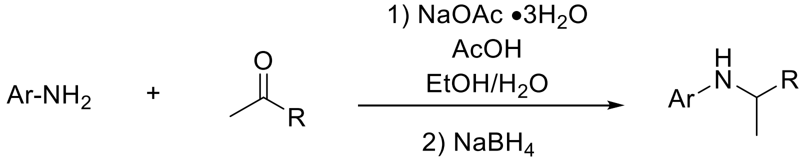

In a 100 mL round bottom flask, amine (5 mmol), ketone (5 mmol), NaOAc⊕3H_2O (10 mmol), glacial acetic acid (4.2 mL), water (12 mL), and ethanol (96%, 3 mL) were combined. The reaction mixture was allowed to stir at room temperature for 30 min, followed by slow addition of $NaBH_4$ (0.9 g). The reaction mixture was allowed to stir for 24 h at room temperature (conversion monitored by TLC). Then, ethyl acetate (100 mL) was added. The organic layer was separated, washed with water (2 x 100 mL), dried over Na_2SO_4, filtered, and the volatiles from the filtrate were removed under reduced pressure. The residue was subjected to flash column chromatography (silica gel, 5% to 20% ethyl acetate in hexanes) to isolate the pure material.

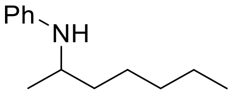

***N-(heptan-2-yl)aniline* 1c.** Following the above procedure, the title compound was obtained as colourless oil, using aniline (0.5 g, 5 mmol) and 2-heptanone (0.6 g, 5 mmol) and purified with silica-gel column chromatography, eluting with 5% to 10% diethyl ether in petroleum ether (containing 0.2% triethylamine) (66%, 0.63 g).[4]

^{1}H NMR (500 MHz, CDCl$_3$) δ 7.21 – 7.11 (m, 2H), 6.69 – 6.66 (m, 1H), 6.60 – 6.57 (m, 2H), 3.50 – 3.43 (m, 2H), 1.63 – 1.52 (m, 1H), 1.48 – 1.37 (m, 3H), 1.36 – 1.29 (m, 4H), 1.19 (d, *J* = 6.2 Hz, 3H), 0.93 – 0.88 (m, 3H).

^{13}C NMR (126 MHz, CDCl$_3$) δ 147.9, 129.4, 116.9, 113.2, 48.6, 37.3, 32.0, 26.0, 22.8, 20.9, 14.2.

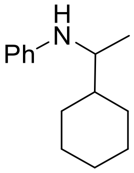

***N*-(1-cyclohexylethyl)aniline 1d.** Following the above procedure, the title compound was obtained as colourless oil, using aniline (0.5 g, 5 mmol) and 1-cyclohexylethan-1-one (0.6 g, 5 mmol) and purified with silica-gel column chromatography, eluting with 5% to 10% diethyl ether in petroleum ether (containing 0.2% triethylamine) (56%, 0.57 g).[4]

^{1}H NMR (500 MHz, CDCl$_3$) δ 7.21 – 7.14 (m, 2H), 6.67 (tt, *J* = 7.3, 1.1 Hz, 1H), 6.62 – 6.56 (m, 2H), 3.49 (brs, 1H), 3.39 – 3.30 (m, 1H), 1.88 – 1.66 (m, 5H), 1.51 – 1.44 (m, 1H), 1.34 – 1. 04 (m, 8H).

^{13}C NMR (126 MHz, CDCl$_3$) δ 148.1, 129.4, 116.6, 113.1, 53.1, 43.1, 29.9, 28.5, 26.8, 26.6, 26.5, 17.6.

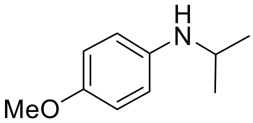

***N*-isopropyl-4-methoxyaniline 1h.** Following the above procedure, the title compound was obtained as colourless oil, using p-anisidine (0.6 g, 5 mmol) and acetone (0.6 g, 10 mmol) and purified with silica-gel column chromatography, eluting with a gradient 10% to 20% of diethyl ether in petroleum ether (containing 0.2% triethylamine) (85%, 0.71 g).[5]

^{1}H NMR (500 MHz, Chloroform-*d*) δ 6.83 – 6.71 (m, 2H), 6.61 – 6.53 (m, 2H), 3.75 (s, 3H), 3.55 (h, *J* = 6.3, 1H), 3.14 (brs, 1H), 1.19 (dd, *J* = 6.3, 1.0 Hz, 6H).

^{13}C NMR (126 MHz, CDCl$_3$) δ 152.1, 141.9, 115.0, 115.0, 55.9, 45.3, 23.2.

Substrates **1e** was prepared according to the literature procedure.[6]

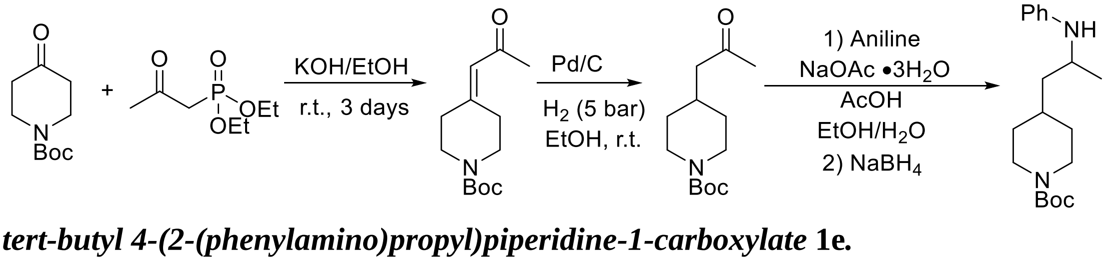

***tert*-butyl 4-(2-(phenylamino)propyl)piperidine-1-carboxylate 1e.**

To a solution of diethyl 2-oxopropylphosphonate (2.5 g, 13 mmol) and KOH (0.6 g, 11 mol) in ethanol (12 mL) at 0 °C, *tert*-butyl 4-oxopiperidine-1-carboxylate (2.0 g, 10 mmol) was added. The mixture was allowed to warm to room temperature and stir at this temperature for 3 days. Then, the volatiles from the mixture were removed under reduced pressure. The residue was triturated with diethyl ether (50 mL) at room

temperature. The combined ether extracts were dried over Na_2SO_4, and filtered. The volatiles from the filtrate were removed under reduced pressure. The reside was subjected to column chromatography with a mixture of ethyl acetate and hexanes (2/3) to afford the α,β-unsaturated ketone as a white solid (1.5 g). The material (1.5 g) was dissolved in ethanol (8 mL) and Pd/C (0.1 g) was added, the mixture was then transferred into a high-pressure reactor, which was then charged with hydrogen (15 bar). The reaction mixture was allowed to stir at room temperature for 5 h. The hydrogen gas was released in a well-ventilated fume hood. The mixture was filtered through a plug of celite, and the volatiles from the filtrate were removed under reduced pressure to afford the ketone intermediate (1.5 g), which was subjected to the next step without any purification. Specifically, the ketone intermediate (1.5 g) was subjected to the reaction with aniline (0.6 g, 6 mmol) as described in above Method B. The title compound was obtained as white solid using silica-gel column chromatography, eluting with 12% diethyl ether in petroleum ether (no pressure column) (22%, 0.42 g).

^{1}H NMR (400 MHz, $CDCl_3$) δ 7.20 – 7.11 (m, 2H), 6.67 (tt, *J* = 7.2, 1.1 Hz, 1H), 6.60 – 6.53 (m, 2H), 4.07 (brs, 2H), 3.58 (h, *J* = 6.3 Hz, 1H), 3.36 (brs, 1H), 2.79 - 2.57 (m, 2H), 1.68 (t, *J* = 9.6 Hz, 2H), 1.63 – 1.58 (m, 1H), 1.55 – 1.48 (m, 1H), 1.46 (s, 9H), 1.36 – 1.29 (m, 1H), 1.23 – 1.03 (m, 5H).

^{13}C NMR (101 MHz, $CDCl_3$) δ 155.0, 147.6, 129.5, 117.1, 113.2, 79.3, 45.7, 44.6, 33.1, 32.5, 32.4, 28.6, 21.3.

HRMS (ESI, m/z): calculated for $C_{19}H_{31}N_2O_2$ $[M+H]^+$: 319.2386, found: 319.2379.

4. The effect of the ratio between $(Cy_3P)_2Pd$ / $RhCl(PPh_3)_3$ complexes & the influence of the $(Cy_3P)_2Pd$ quality

The initial development of the model reaction and the optimization of the conditions were performed with a single batch of $(Cy_3P)_2Pd$ complex. These experiments identified the highest yield of the reaction (68%) in the presence of 4 mol% of $(Cy_3P)_2Pd$ complex and 5 mol% of $RhCl(PPh_3)_3$. The yield of the reaction was reproducible (± <5%) when using the same materials. However, the subsequent experiments with other batches of $(Cy_3P)_2Pd$ complex indicated the diminished reproducibility of the reaction, with the yield varying by ± >15% between the experiments conducted with different batches of $(Cy_3P)_2Pd$. (The experiments with different batches of $RhCl(PPh_3)_3$, whether commercial or prepared in house, were reproducible). The subsequent analysis, which is summarized in Figure S2 and detailed in Section 5, showed that: (i) Commercially available batches of '$(Cy_3P)_2Pd$' may contain <50% or even trace amounts of the actual $(Cy_3P)_2Pd$ complex, depending on the supplier, as judged by the $^{31}P\{^1H\}$ NMR spectra. (ii) The spectroscopically pure $(Cy_3P)_2Pd$ complex can be readily prepared, following an excellent literature procedure described and cited in Section 2. (iii) The spectroscopically pure $(Cy_3P)_2Pd$ complex can be stored at -40 °C for months in a nitrogen-filled glovebox (<5% decomposition after 3 months, as judged by the $^{31}P\{^1H\}$ NMR spectroscopy). However, (iv) $(Cy_3P)_2Pd$ decomposes readily at ambient temperature in a nitrogen-filled glovebox (~50% decomposition after 1 week at room temperature, as judged by the $^{31}P\{^1H\}$ NMR spectroscopy). (v) Experiments with varied amounts of spectroscopically pure $(Cy_3P)_2Pd$ and varied amounts of $RhCl(PPh_3)_3$ indicated the yield is sensitive to the ratio of Pd to Rh, with higher amounts of (spectroscopically pure) $(Cy_3P)_2Pd$ lowering the yield of the reaction, as shown in Figure S1. Thus, the quality and storage of $(Cy_3P)_2Pd$ must be taken into account in the catalytic experiments to ensure the optimal performance of the reaction.

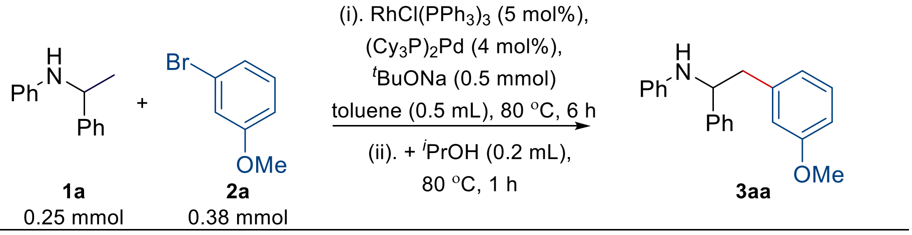

fixed 5 mol% [Rh]

[Pd]/ (mol%)	**3aa**, nmr yield (%)
1	65
2	62
4	56
6	45
8	40

fixed 4 mol% [Pd]

[Rh]/ (mol%)	**3aa**, nmr yield (%)
1.0	9
2.5	26
5.0	56
7.5	61
10.0	62

Figure S1. Results of the standard reaction with the varied ratio of $(Cy_3P)_2Pd$/ $RhCl(PPh_3)_3$ complexes - the experiments with spectroscopically pure $(Cy_3P)_2Pd$.

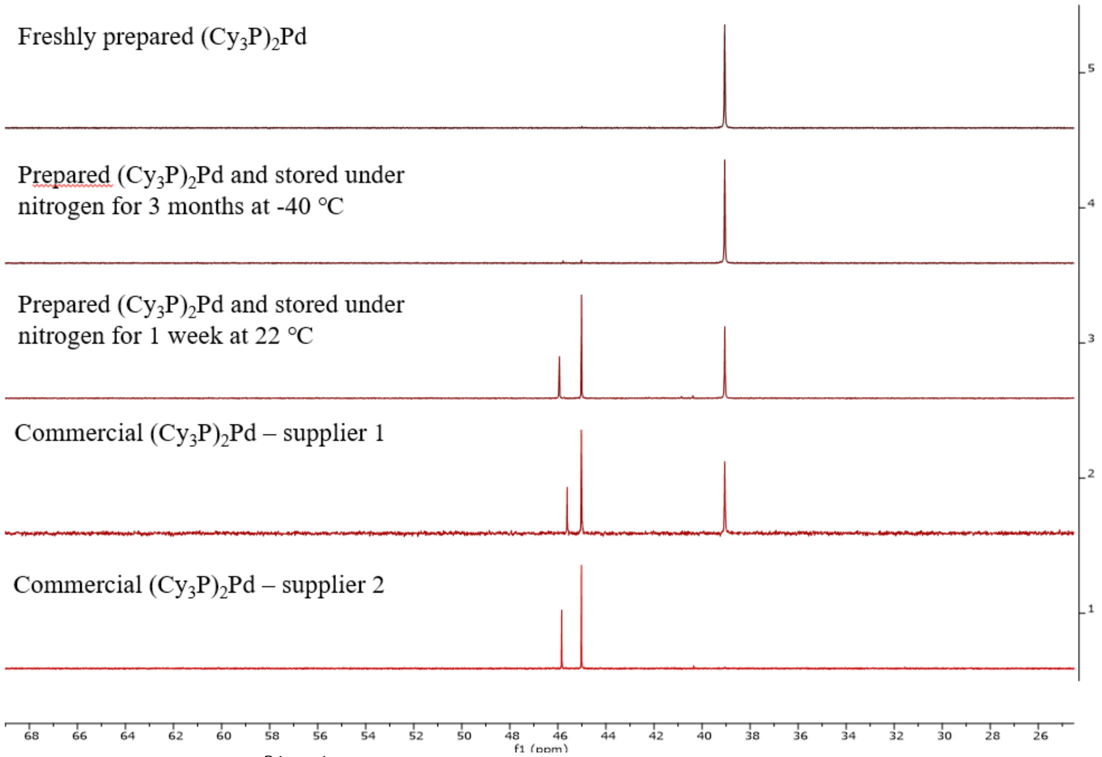

Figure S2. $^{31}P\{^{1}H\}$ NMR spectra of different batches of $(Cy_3P)_2Pd$.

5. ^{31}P NMR spectra of prepared and commercial $(Cy_3P)_2Pd$

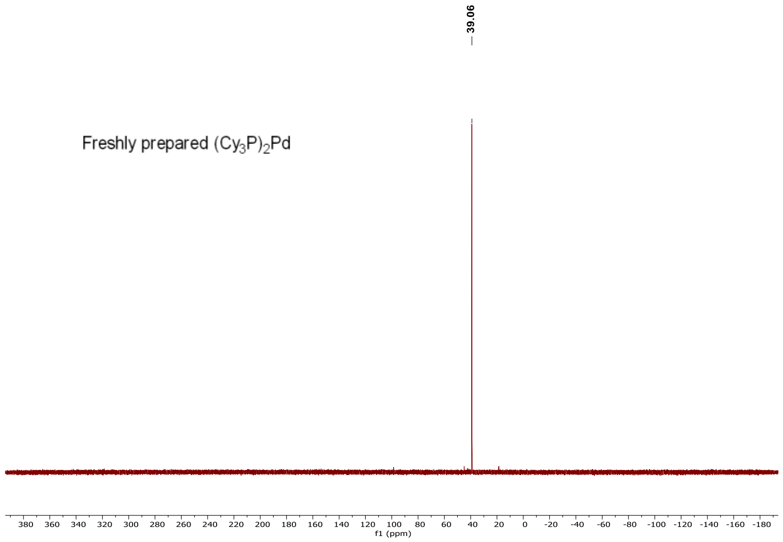

Figure S3. ^{31}P{^{1}H} NMR spectrum of freshly prepared $(Cy_3P)_2Pd$.

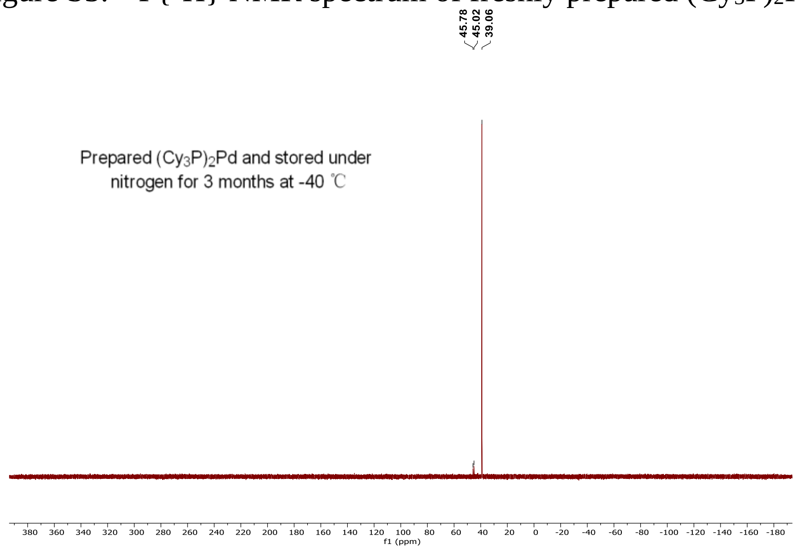

Figure S4. ^{31}P{^{1}H} NMR spectrum of prepared $(Cy_3P)_2Pd$ and stored under nitrogen for 3 months at -40 °C.

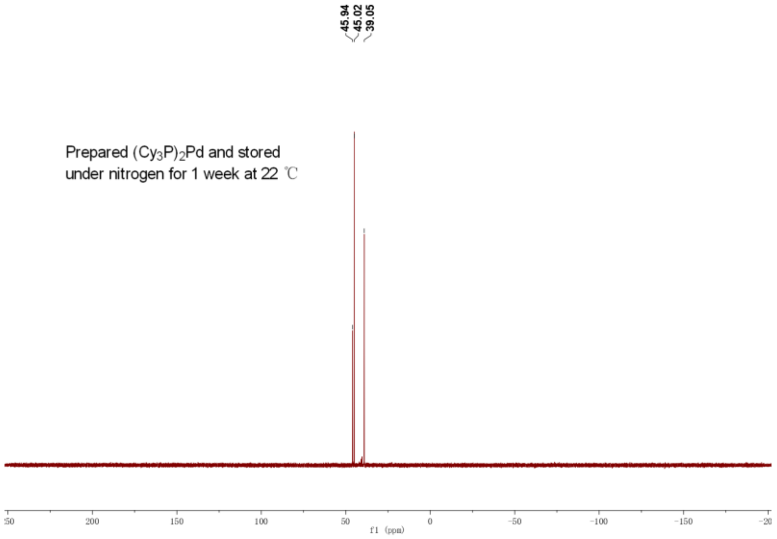

Figure S5. $^{31}P\{^{1}H\}$ NMR spectrum of prepared $(Cy_3P)_2Pd$ and stored under nitrogen for 1 week at 22 °C.

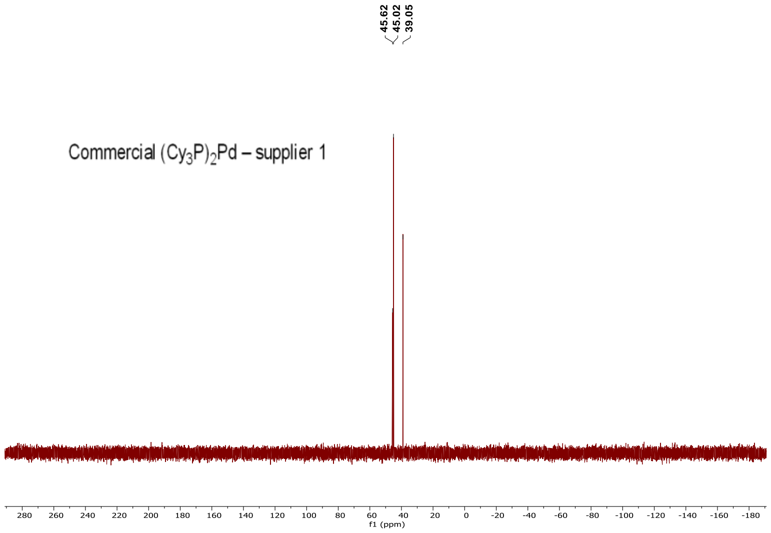

Figure S6. $^{31}P\{^{1}H\}$ NMR spectrum of commercial $(Cy_3P)_2Pd$ – supplier 1.

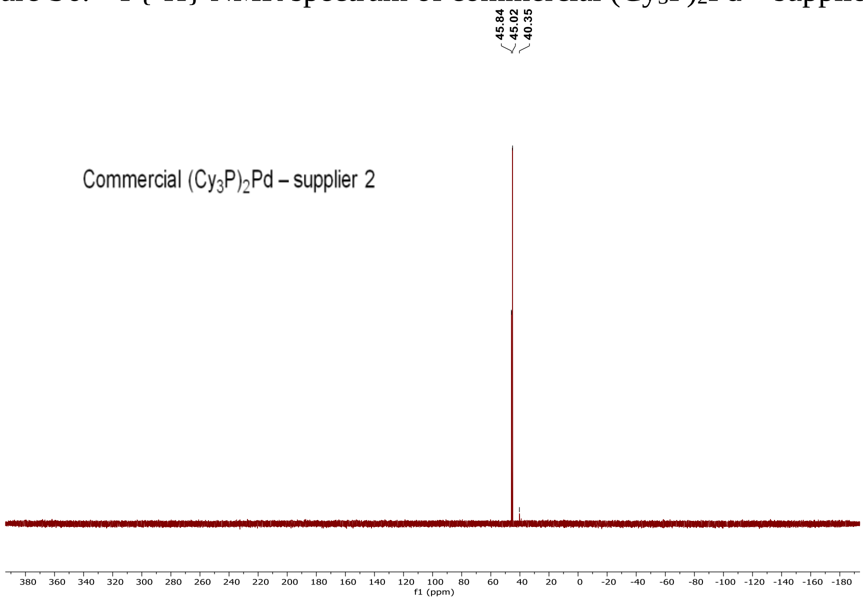

Figure S7. $^{31}P\{^{1}H\}$ NMR spectrum of commercial $(Cy_3P)_2Pd$ – supplier 2.

6. General procedure for the preparation of β-arylamines

Under an inert atmosphere, amine (0.25 mmol, 1 equiv.), aryl bromide (0.38 mmol, 1.5 equiv.), NaO^{t}Bu (48.3 mg, 2 equiv.), Wilkinson's catalyst (11.6 mg, 5 mol%), and $(Cy_3P)_2Pd$ (6.7 mg, 4 mol%) were suspended in toluene (0.5 mL) in a 4 mL vial equipped with a stir bar. It was then sealed, placed in a preheated oil bath, and allowed to stir at the described temperature (typically 80 °C) for the stated time (typically 6 h). The vial was allowed to cool to room temperature and ^{i}PrOH (0.2 mL) was added under inert atmosphere. The vial was resealed, and the reaction mixture was allowed to stir for 1 h at the described temperature. (Prior to the addition of ^{i}PrOH, the GCMS analysis of the reaction mixture showed the presence of both arylated amine and arylated imine, indicating incomplete reduction. Therefore, to recover the target arylated amine in a higher yield, ^{i}PrOH was added to shift the equilibrium toward the amine. Through the oxidation of ^{i}PrOH to acetone, a hydrogenated Rh catalyst is formed in excess, which results in the complete reduction of the imine to the amine.) After cooling to room temperature, the contents of the vial were filtered through a small plug of Celite (~ 2 g). The Celite plug was washed with ethyl acetate (~10 mL). The volatiles from the combined filtrates were removed under reduced pressure. The target product was isolated from the residue via column chromatography on silica gel (either manual column or using a CombiFlash instrument), typically using a mixture of petroleum ether and ethyl acetate (typically a gradient from 100:0 to 80:20, 0.1% NEt_3 was usually added) as the eluent. Fractions containing the pure product (judged by TLC and/or GC-MS analyses) were combined, and the solvents were removed under reduced pressure to yield the target product.

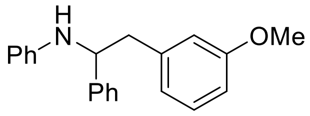

***N*-(2-(3-methoxyphenyl)-1-phenylethyl)aniline** **3aa.** Following the general procedure, the title compound was obtained as pale-yellow oil using *N*-(1-phenylethyl)aniline (49.3 mg, 0.25 mmol) and 1-bromo-3-methoxybenzene (70.2 mg, 0.38 mmol) and purified with silica gold column chromatography, eluting with a gradient (0-20%) of diethyl ether in petroleum ether (containing 0.1% triethylamine) (54%, 41.1 mg).[7]

^{1}H NMR (400 MHz, $CDCl_3$) δ 7.31 – 7.20 (m, 4H), 7.20 – 7.09 (m, 2H), 7.03 – 6.94 (m, 2H), 6.72 – 6.66 (m, 2H), 6.60 – 6.52 (m, 2H), 6.40 (dd, *J* = 8.7, 1.0 Hz, 2H), 4.52 (dd, *J* = 8.2, 5.6 Hz, 1H), 4.07 (brs, 1H), 3.66 (s, 3H), 3.05 (dd, *J* = 13.9, 5.6 Hz, 1H), 2.92 (dd, *J* = 13.9, 8.2 Hz, 1H).

^{13}C NMR (101 MHz, $CDCl_3$) δ 159.8, 147.4, 143.5, 139.3, 129.6, 129.1, 128.7, 127.2, 126.6, 121.7, 117.6, 115.0, 113.8, 112.3, 59.2, 55.2, 45.3.

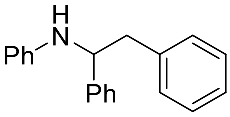

N-(1,2-diphenylethyl)aniline **3ab.** Following the general procedure, the title compound was obtained as colourless oil using *N*-(1-phenylethyl)aniline (50 μL, 0.25 mmol) and bromobenzene (59.3 mg, 0.38 mmol) and purified with silica gold column chromatography, eluting with 2% diethyl ether in n-pentane (44%, 30.0 mg).[8]

^{1}H NMR (400 MHz, CDCl$_3$) δ 7.44 – 7.28 (m, 8H), 7.21 (d, J = 6.7 Hz, 2H), 7.13 (t, J = 8.0 Hz, 2H), 6.71 (t, J = 7.3 Hz, 1H), 6.54 (d, J = 8.0 Hz, 2H), 4.67 (dd, J = 8.2, 5.7 Hz, 1H), 4.22 (bs, 1H), 3.22 (dd, J = 14.0, 5.7 Hz, 1H), 3.10 (dd, J = 14.0, 8.2 Hz, 1H).

^{13}C NMR (101 MHz, CDCl$_3$) δ 147.4, 143.6, 137.8, 129.3, 129.1, 128.7, 128.7, 127.2, 126.9, 126.6, 117.6, 113.8, 59.4, 45.3.

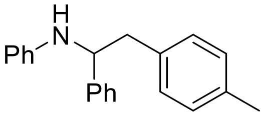

N-(1-phenyl-2-(p-tolyl)ethyl)aniline **3ac.** Following the general procedure, the title compound was obtained as pale-yellow oil using *N*-(1-phenylethyl)aniline (50 μL, 0.25 mmol) and 1-bromo-4-methylbenzene (63.5 mg, 0.38 mmol) and purified with silica gold column chromatography, eluting with a gradient (0-20%) of diethyl ether in petroleum ether (containing 0.1% triethylamine, 2% toluene) (48%, 34.4 mg).[8]

^{1}H NMR (500 MHz, CDCl$_3$) δ 7.38 – 7.29 (m, 4H), 7.29 – 7.21 (m, 1H), 7.13 – 7.00 (m, 6H), 6.69 – 6.60 (m, 1H), 6.48 – 6.46 (m, 2H), 4.57 (dd, *J* = 8.3, 5.6 Hz, 1H), 4.13 (brs, 1H), 3.12 (dd, *J* = 14.1, 5.5 Hz, 1H), 2.98 (dd, *J* = 14.1, 8.3 Hz, 1H), 2.33 (s, 3H).

^{13}C NMR (126 MHz, CDCl$_3$) δ 147.5, 143.7, 136.4, 134.7, 129.4, 129.2, 129.1, 128.7, 127.1, 126.6, 117.6, 113.8, 59.4, 44.9, 21.2.

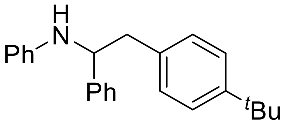

N-(2-(4-(tert-butyl)phenyl)-1-phenylethyl)aniline **3ad.** Following the general procedure, the title compound was obtained as colourless oil using *N*-(1-phenylethyl)aniline (50 μL, 0.25 mmol) and 1-bromo-4-(*tert*-butyl)benzene (80.6 mg, 0.38 mmol) and purified with silica gold column chromatography, eluting with a gradient (0-20%) of diethyl ether in petroleum ether (47%, 38.8 mg).[9]

^{1}H NMR (400 MHz, CDCl$_3$) δ 7.34 – 7.29 (m, 2H), 7.29 – 7.22 (m, 4H), 7.22 – 7.14 (m, 1H), 7.08 – 7.02 (m, 2H), 7.02 – 6.97 (m, 2H), 6.57 (tt, J = 7.3, 1.1 Hz, 1H), 6.41 (dd, J = 8.7, 1.1 Hz, 2H), 4.52 (dd, J = 8.8, 5.1 Hz, 1H), 4.11 (bs, 1H), 3.08 (dd, J = 14.2, 5.1 Hz, 1H), 2.89 (dd, J = 14.2, 8.8 Hz, 1H), 1.26 (s, 9H).

^{13}C NMR (101 MHz, CDCl$_3$) δ 149.7, 147.5, 143.9, 134.8, 129.1, 128.9, 128.7, 127.1, 126.5, 125.6, 117.5, 113.8, 59.2, 44.9, 34.6, 31.5.

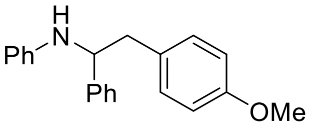

***N-(2-(4-methoxyphenyl)-1-phenylethyl)aniline* 3ae.** Following the general procedure, the title compound was obtained as white crystals using *N*-(1-phenylethyl)aniline (50 μL, 0.25 mmol) and 1-bromo-4-methoxybenzene (70.2 mg, 0.38 mmol) and purified with silica gold column chromatography, eluting with a gradient (0-20%) of methanol in petroleum ether (containing 0.1% triethylamine) (47%, 35.4 mg).[10]

^{1}H NMR (400 MHz, CDCl$_3$) δ 7.30 – 7.12 (m, 5H), 7.05 – 6.93 (m, 4H), 6.81 – 6.71 (m, 2H), 6.58 (tt, J = 7.2, 1.1 Hz, 1H), 6.42 (dd, J = 8.6, 1.1 Hz, 2H), 4.48 (dd, J = 8.0, 5.8 Hz, 1H), 4.20 (brs, 1H), 3.72 (s, 3H), 3.03 (dd, J = 14.0, 5.8 Hz, 1H), 2.92 (dd, J = 14.0, 8.0 Hz, 1H).

^{13}C NMR (101 MHz, CDCl$_3$) δ 158.5, 147.2, 143.4, 130.3, 129.7, 129.1, 128.7, 127.2, 126.6, 117.7, 114.0, 113.9, 59.6, 55.4, 44.3.

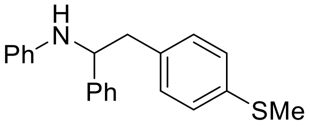

***N-(2-(4-(methylthio)phenyl)-1-phenylethyl)aniline* 3af.** Following the general procedure, the title compound was obtained as yellow oil using *N*-(1-phenylethyl)aniline (50 μL, 0.25 mmol) and (4-bromophenyl)(methyl)sulfane (76.8 mg, 0.38 mmol) and purified with silica gold column chromatography, eluting with a gradient (0-20%) of diethyl ether in petroleum ether (37%, 29.7 mg).[11]

^{1}H NMR (400 MHz, CDCl$_3$) δ 7.28 – 7.19 (m, 4H), 7.19 – 7.11 (m, 1H), 7.10 – 7.02 (m, 2H), 7.02 – 6.88 (m, 4H), 6.56 (tt, J = 7.3, 1.1 Hz, 1H), 6.40 (dd, J = 8.7, 1.1 Hz, 2H), 4.48 (dd, J = 7.9, 6.0 Hz, 1H), 4.23 (brs, 1H), 3.02 (dd, J = 14.0, 6.0 Hz, 1H), 2.93 (dd, J = 14.0, 7.9 Hz, 1H), 2.38 (s, 3H).

^{13}C NMR (101 MHz, CDCl$_3$) δ 147.0, 143.1, 136.7, 134.6, 129.8, 129.2, 128.7, 127.3, 127.0, 126.7, 117.9, 114.0, 59.5, 44.5, 16.1.

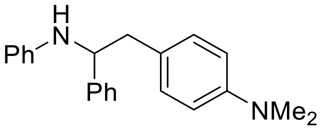

***N,N-dimethyl-4-(2-phenyl-2-(phenylamino)ethyl)aniline* 3ag.** Following the general procedure, the title compound was obtained as colourless oil using *N*-(1-phenylethyl)aniline (50 μL, 0.25 mmol) and 4-bromo-*N,N*-dimethylaniline (75.6 mg, 0.38 mmol) and purified with silica gold column chromatography, eluting with a gradient (0-20%) of diethyl ether in petroleum ether (47%, 37.3 mg).

^{1}H NMR (400 MHz, CDCl$_3$) δ 7.29 – 7.19 (m, 4H), 7.18 – 7.11 (m, 1H), 6.99 – 6.88 (m, 4H), 6.61 – 6.56 (m, 2H), 6.53 (tt, J = 7.3, 1.1 Hz, 1H), 6.40 – 6.34 (m, 2H), 4.44

(dd, J = 8.4, 5.3 Hz, 1H), 4.07 (s, 1H), 2.99 (dd, J = 14.1, 5.4 Hz, 1H), 2.84 (s, 6H), 2.80 (dd, J = 14.1, 8.4 Hz, 1H).

^{13}C NMR (101 MHz, CDCl$_3$) δ 149.7, 147.7, 144.1, 130.1, 129.2, 128.7, 127.1, 126.7, 125.6, 117.5, 113.9, 113.1, 59.5, 44.5, 40.9.

HRMS (ESI, m/z): calculated for $C_{22}H_{25}N_2$ $[M+H]^+$: 318.2046, found: 318.2031.

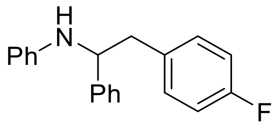

***N-(2-(4-fluorophenyl)-1-phenylethyl)aniline* 3ah.** Following the general procedure, the title compound was obtained as pale- yellow oil using *N*-(1-phenylethyl)aniline (50 μL, 0.25 mmol) and 1-bromo-4-fluorobenzene (66.2 mg, 0.38 mmol) and purified with silica gold column chromatography, eluting with a gradient (0-20%) of diethyl ether in petroleum ether (49%, 35.6 mg).[9]

^{1}H NMR (400 MHz, CDCl$_3$) δ 7.31 – 7.17 (m, 5H), 7.08 – 6.96 (m, 4H), 6.96 – 6.87 (m, 2H), 6.63 (tt, J = 7.4, 1.1 Hz, 1H), 6.46 (dd, J = 8.7, 1.1 Hz, 2H), 4.53 (dd, J = 7.6, 6.3 Hz, 1H), 4.23 (brs, 1H), 3.11 – 2.96 (m, 2H).

^{13}C NMR (101 MHz, CDCl$_3$) δ 161.9 (d, J = 244.8 Hz), 147.0, 143.0, 133.4 (d, J = 3.3 Hz), 130.8 (d, J = 7.9 Hz), 129.2, 128.7, 127.3, 126.6, 117.9, 115.4 (d, J = 21.2 Hz), 113.9, 59.6, 44.2.

^{19}F NMR (376 MHz, CDCl$_3$) δ -116.1.

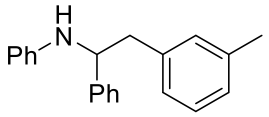

***N-(1-phenyl-2-(m-tolyl)ethyl)aniline* 3ai.** Following the general procedure, the title compound was obtained as pale-yellow oil using *N*-(1-phenylethyl)aniline (50 μL, 0.25 mmol) and 1-bromo-3-methylbenzene (64.6 mg, 0.38 mmol) and purified with silica gold column chromatography, eluting with a gradient (0-20%) of diethyl ether in petroleum ether (containing 0.1% triethylamine, 2% toluene) (43%, 30.9 mg).[8]

^{1}H NMR (400 MHz, CDCl$_3$) δ 7.38 – 7.29 (m, 4H), 7.27 – 7.25 (m, 1H), 7.20 – 7.16 (m, 1H), 7.08 – 7.04 (m, 3H), 6.96 – 6.94 (m, 2H), 6.73 – 6.57 (m, 1H), 6.48 – 6.46 (m, 2H), 4.58 (dd, *J* = 8.5, 5.5 Hz, 1H), 4.14 (brs, 1H), 3.12 (dd, *J* = 13.9, 5.5 Hz, 1H), 2.96 (dd, *J* = 13.9, 8.5 Hz, 1H), 2.32 (s, 3H).

^{13}C NMR (101 MHz, CDCl$_3$) δ 147.5, 143.8, 138.3, 137.7, 130.1, 129.1, 128.7, 128.6, 127.6, 127.2, 126.6, 126.3, 117.6, 113.8, 59.4, 45.4, 21.5.

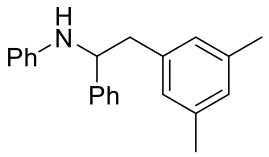

***N*-(2-(3,5-dimethylphenyl)-1-phenylethyl)aniline** **3aj.** Following the general procedure, the title compound was obtained as pale-yellow oil using *N*-(1-phenylethyl)aniline (50 µL, 0.25 mmol) and 1-bromo-3,5-dimethylbenzene (70.0 mg, 0.38 mmol) and purified with silica gold column chromatography, eluting with a gradient (0-20%) of diethyl ether in petroleum ether (containing 0.1% triethylamine, 2% toluene) (45%, 33.9 mg).[7]

^{1}H NMR (400 MHz, CDCl$_3$) δ 7.42 – 7.30 (m, 4H), 7.30 – 7.22 (m, 1H), 7.11 – 7.02 (m, 2H), 6.89 (s, 1H), 6.79 (d, *J* = 1.6 Hz, 2H), 6.66 – 6.62 (m, 1H), 6.52 – 6.42 (m, 2H), 4.57 (dd, *J* = 8.7, 5.2 Hz, 1H), 4.17 (brs, 1H), 3.10 (dd, *J* = 13.9, 5.2 Hz, 1H), 2.90 (dd, *J* = 14.0, 8.8 Hz, 1H), 2.30 (s, 6H).

^{13}C NMR (101 MHz, CDCl$_3$) δ 147.5, 143.9, 138.2, 137.7, 129.1, 128.7, 128.5, 127.1, 127.1, 126.5, 117.5, 113.9, 59.4, 45.4, 21.4.

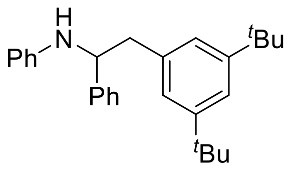

***N*-(2-(3,5-di-tert-butylphenyl)-1-phenylethyl)aniline** **3ak.** Following the general procedure, the title compound was obtained as pale yellow oil using *N*-(1-phenylethyl)aniline (50 µL, 0.25 mmol) and 1-bromo-3,5-di-*tert*-butylbenzene (101.8 mg, 0.38 mmol) and purified with silica gold column chromatography, eluting with a gradient (0-20%) of diethyl ether in petroleum ether (59%, 57.2 mg).

^{1}H NMR (400 MHz, CDCl$_3$) δ 7.30 – 7.23 (m, 5H), 7.23 – 7.15 (m, 1H), 7.07 – 6.99 (m, 2H), 6.85 (d, J = 1.8 Hz, 2H), 6.60 (tt, J = 7.3, 1.1 Hz, 1H), 6.43 (dd, J = 8.7, 1.1 Hz, 2H), 4.53 (dd, J = 7.3, 5.9 Hz, 1H), 4.19 (s, 1H), 3.10 (dd, J = 13.6, 5.9 Hz, 1H), 3.01 (dd, J = 13.6, 7.4 Hz, 1H), 1.25 (s, 18H).

^{13}C NMR (101 MHz, CDCl$_3$) δ 151.0, 147.6, 143.7, 136.6, 129.2, 128.7, 127.1, 126.8, 123.9, 120.8, 117.6, 113.9, 59.4, 45.8, 35.0, 31.7.

HRMS (ESI, m/z): calculated for $C_{28}H_{36}N_1$ $[M+H]^+$: 388.2910, found: 388.2907.

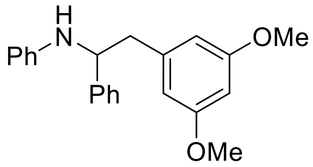

***N*-(2-(3,5-dimethoxyphenyl)-1-phenylethyl)aniline** **3al.** Following the general procedure, the title compound was obtained as colourless oil using *N*-(1-phenylethyl)aniline (50 µL, 0.25 mmol) and 1-bromo-3,5-dimethoxybenzene (82.1 mg, 0.38 mmol) and purified with silica gold column chromatography, eluting with a gradient (0-20%) of diethyl ether in petroleum ether (55%, 45.5 mg).[10]

^{1}H NMR (400 MHz, CDCl$_3$) δ 7.31 – 7.21 (m, 4H), 7.20 – 7.13 (m, 1H), 7.02 – 6.95 (m, 2H), 6.56 (tt, J = 7.3, 1.1 Hz, 1H), 6.40 (dd, J = 8.7, 1.1 Hz, 2H), 6.26 (t, J = 2.3

Hz, 1H), 6.19 (d, J = 2.3 Hz, 2H), 4.50 (dd, J = 8.1, 5.6 Hz, 1H), 4.13 (bs, 1H), 3.64 (s, 6H), 3.01 (dd, J = 13.8, 5.6 Hz, 1H), 2.88 (dd, J = 13.8, 8.1 Hz, 1H).
^{13}C NMR (101 MHz, CDCl$_3$) δ 161.0, 147.4, 143.6, 140.1, 129.2, 128.8, 127.3, 126.7, 117.7, 113.9, 107.5, 99.0, 59.3, 55.5, 45.6.

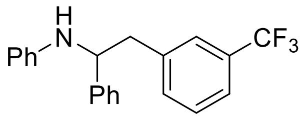

N-(1-phenyl-2-(3-(trifluoromethyl)phenyl)ethyl)aniline **3am.** Following the general procedure, the title compound was obtained as pale- yellow oil using *N*-(1-phenylethyl)aniline (50 μL, 0.25 mmol) and 1-bromo-3-(trifluoromethyl)benzene (85.1 mg, 0.38 mmol) and purified with silica gold column chromatography, eluting with a gradient (0-20%) of methanol in petroleum ether (containing 0.1% triethylamine) (36%, 30.8 mg).[11]
^{1}H NMR (400 MHz, CDCl$_3$) δ 7.44 (d, J = 7.7 Hz, 1H), 7.37 – 7.24 (m, 1H), 7.27 – 7.15 (m, 7H), 7.08 – 6.99 (m, 2H), 6.62 (tt, J = 7.3, 1.1 Hz, 1H), 6.46 (d, J = 7.5 Hz, 2H), 4.57 (t, J = 6.9 Hz, 1H), 4.05 (bs, 1H), 3.10 (m, 2H).
^{13}C NMR (101 MHz, CDCl$_3$) δ 147.0, 142.7, 138.8, 132.7, 130.8 (d, J = 32.1 Hz), 129.3, 129.0, 128.8, 127.5, 126.6, 126.2 (d, J = 3.8 Hz), 124.2 (q, J = 272.4Hz), 123.7 (q, J = 3.9 Hz), 117.9, 113.8, 59.3, 44.8.
^{19}F NMR (376 MHz, CDCl$_3$) δ -62.6.

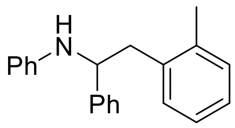

N-(1-phenyl-2-(o-tolyl)ethyl)aniline **3an.** Following the general procedure, the title compound was obtained as yellow oil using *N*-(1-phenylethyl)aniline (50 μL, 0.25 mmol) and 1-bromo-2-methylbenzene (64.6 mg, 0.38 mmol) and purified with silica gold column chromatography, eluting with a gradient (0-20%) of diethyl ether in petroleum ether (containing 0.1% triethylamine) (82%, 58.8 mg).[8]
^{1}H NMR (400 MHz, CDCl$_3$) δ 7.44 – 7.33 (m, 4H), 7.33 – 7.26 (m, 1H), 7.23 – 7.15 (m, 4H), 7.15 – 7.08 (m, 2H), 6.70 (tt, J = 7.3, 1.1 Hz, 1H), 6.56 – 6.50 (m, 2H), 4.64 (dd, J = 8.4, 5.9 Hz, 1H), 4.32 (br s, 1H), 3.17 (dd, J = 14.3, 5.9 Hz, 1H), 3.09 (dd, J = 14.3, 8.4 Hz, 1H), 2.31 (s, 3H).
^{13}C NMR (101 MHz, CDCl$_3$) δ 147.4, 143.8, 136.8, 136.1, 130.7, 129.7, 129.1, 128.7, 127.2, 126.9, 126.4, 126.2, 117.7, 113.9, 58.4, 42.8, 19.6.

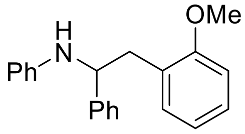

N-(2-(2-methoxyphenyl)-1-phenylethyl)aniline **3ao.** Following the general procedure, the title compound was obtained as white crystals using *N*-(1-phenylethyl)aniline (50 μL, 0.25 mmol) and 1-bromo-2-methoxybenzene (70.7 mg, 0.38 mmol) and purified with silica gold column chromatography, eluting with a gradient (0-20%) of diethyl ether in petroleum (57%, 42.9 mg).

^{1}H NMR (400 MHz, CDCl$_3$) δ 7.47 – 7.39 (m, 2H), 7.39 – 7.30 (m, 2H), 7.33 – 7.19 (m, 2H), 7.11 – 7.02 (m, 3H), 6.95 – 6.85 (m, 2H), 6.62 (t, J = 7.3 Hz, 1H), 6.47 (d, J = 7.6 Hz, 2H), 4.86 (brs, 1H), 4.59 (dd, J = 8.9, 4.8 Hz, 1H), 3.93 (s, 3H), 3.16 (dd, J = 13.8, 9.0 Hz, 1H), 3.06 (dd, J = 13.8, 4.8 Hz, 1H).

^{13}C NMR (101 MHz, CDCl$_3$) δ 157.8, 147.8, 144.4, 131.2, 129.1, 128.7, 128.2, 127.1, 127.1, 126.6, 120.9, 117.1, 113.6, 110.7, 59.8, 55.5, 40.1.

HRMS (ESI, m/z): calculated for $C_{21}H_{22}NO$ [M+H]$^+$: 305.1730, found: 305.1714.

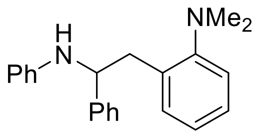

N,N-dimethyl-2-(2-phenyl-2-(phenylamino)ethyl)aniline **3ap.** Following the general procedure, the title compound was obtained as yellow oil using *N*-(1-phenylethyl)aniline (50 μL, 0.25 mmol) and 2-bromo-*N,N*-dimethylaniline (75.6 mg, 0.38 mmol) and purified with silica gold column chromatography, eluting with a gradient (0-20%) of diethyl ether in petroleum ether (62%, 49.2 mg).

^{1}H NMR (400 MHz, CDCl$_3$) δ 7.35 (dt, J = 8.1, 1.9 Hz, 2H), 7.30 – 7.20 (m, 2H), 7.18 – 7.12 (m, 1H), 7.12 – 7.07 (m, 2H), 7.05 – 7.00 (m, 1H), 6.96 – 6.87 (m, 3H), 6.44 (tt, J = 7.2, 1.1 Hz, 1H), 6.28 (dd, J = 8.7, 1.1 Hz, 2H), 6.14 (s, 1H), 4.35 (dd, J = 9.9, 3.3 Hz, 1H), 3.18 (dd, J = 13.8, 9.9 Hz, 1H), 2.84 (dd, J = 13.7, 3.3 Hz, 1H), 2.72 (s, 6H).

^{13}C NMR (101 MHz, CDCl$_3$) δ 153.0, 148.2, 144.8, 134.8, 131.6, 129.1, 128.8, 128.0, 127.1, 126.5, 124.8, 120.2, 116.3, 113.0, 61.7, 45.7, 41.6.

HRMS (ESI, m/z): calculated for $C_{22}H_{25}N_2$ [M+H]$^+$: 318.2048, found: 318.2032.

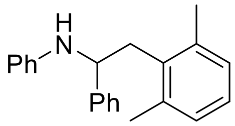

N-(2-(2,6-dimethylphenyl)-1-phenylethyl)aniline **3aq.** Following the general procedure, the title compound was obtained as yellow oil using *N*-(1-phenylethyl)aniline (50 μL, 0.25 mmol) and 2-bromo-1,3-dimethylbenzene (69.9 mg, 0.38 mmol) and purified with silica gold column chromatography, eluting with 2% diethyl ether in n-pentane (47%, 35.0 mg).

^{1}H NMR (400 MHz, CDCl$_3$) δ 7.32 – 7.21 (m, 5H), 7.13 – 6.99 (m, 5H), 6.66 (t, J = 7.3 Hz, 1H), 6.46 (d, J = 7.6 Hz, 2H), 4.56 (t, J = 7.4 Hz, 1H), 4.25 (s, 1H), 3.20 (dd, J = 13.6, 7.0 Hz, 1H), 3.11 (dd, J = 13.6, 7.8 Hz, 1H), 2.22 (s, 6H).

^{13}C NMR (101 MHz, CDCl$_3$) δ 147.6, 143.7, 137.5, 134.7, 129.2, 128.7, 128.6, 127.2, 126.8, 126.5, 117.7, 114.0, 58.2, 39.4, 20.5.
HRMS (ESI, m/z): calculated for $C_{22}H_{24}N$ $[M+H]^+$: 303.1937, found: 303.1922.

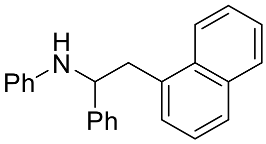

***N*-(2-(naphthalen-1-yl)-1-phenylethyl)aniline** **3ar.** Following the general procedure, the title compound was obtained as yellow oil using *N*-(1-phenylethyl)aniline (50 μL, 0.25 mmol) and 1-bromonaphthalene (78.3 mg, 0.38 mmol) and purified with silica gold column chromatography, eluting with a gradient (0-20%) of methanol in petroleum ether (containing 0.1% triethylamine) (55%, 44.4 mg).[8]
^{1}H NMR (400 MHz, CDCl$_3$) δ 8.06 (d, J = 8.6 Hz, 1H), 7.90 – 7.75 (m, 1H), 7.69 (d, J = 8.2 Hz, 1H), 7.52 – 7.36 (m, 2H), 7.36 – 7.30 (m, 3H), 7.29 – 7.20 (m, 2H), 7.18 (td, J = 7.1, 1.4 Hz, 2H), 6.99 – 6.90 (m, 2H), 6.53 (tt, J = 7.4, 1.1 Hz, 1H), 6.35 (dd, J = 8.6, 1.1 Hz, 2H), 4.70 (dd, J = 8.8, 5.3 Hz, 1H), 4.15 (bs, 1H), 3.51 (dd, J = 14.4, 5.3 Hz, 1H), 3.36 (dd, J = 14.4, 8.8 Hz, 1H).
^{13}C NMR (101 MHz, CDCl$_3$) δ 147.4, 144.1, 134.1, 133.9, 132.3, 129.1, 129.1, 128.8, 127.8, 127.3, 127.3, 126.4, 126.4, 125.9, 125.5, 123.6, 117.6, 113.8, 58.6, 42.8.

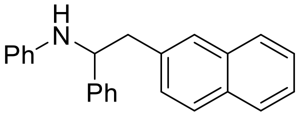

***N*-(2-(naphthalen-2-yl)-1-phenylethyl)aniline** **3as.** Following the general procedure, the title compound was obtained as pale yellow oil using *N*-(1-phenylethyl)aniline (50 μL, 0.25 mmol) and 2-bromonaphthalene (78.3 mg, 0.38 mmol) and purified with silica gold column chromatography, eluting with 2% diethyl ether in n-pentane (32%, 26.0 mg).[8]
^{1}H NMR (400 MHz, CDCl$_3$) δ 7.87 – 7.74 (m, 3H), 7.65 – 7.59 (m, 1H), 7.54 – 7.45 (m, 2H), 7.43 – 7.30 (m, 4H), 7.32 – 7.21 (m, 3H), 7.13 – 6.98 (m, 2H), 6.65 (tt, J = 7.3, 1.1 Hz, 1H), 6.50 – 6.42 (m, 1H), 4.74 (dd, J = 8.2, 5.7 Hz, 1H), 4.20 (bs, 1H), 3.33 (dd, J = 14.0, 5.6 Hz, 1H), 3.21 (dd, J = 14.0, 8.2 Hz, 1H).
^{13}C NMR (101 MHz, CDCl$_3$) δ 147.5, 143.6, 135.4, 133.7, 132.6, 129.2, 128.9, 128.4, 128.1, 127.9, 127.8, 127.6, 127.3, 126.7, 126.3, 125.8, 117.8, 113.9, 59.3, 45.6.

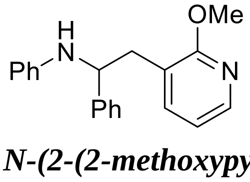

***N*-(2-(2-methoxypyridin-3-yl)-1-phenylethyl)aniline** **3at.** Following the general procedure, the title compound was obtained as pale-yellow oil using *N*-(1-phenylethyl)aniline (50 μL, 0.25 mmol) and 3-bromo-2-methoxypyridine (71.1 mg,

0.38 mmol) and purified with silica gold column chromatography, eluting with a gradient (0-20%) of diethyl ether in petroleum ether (containing 0.1% triethylamine, 2% toluene) (42%, 32.0 mg).

^{1}H NMR (400 MHz, $CDCl_3$) δ 8.05 (dd, *J* = 5.0, 1.8 Hz, 1H), 7.40 – 7.27 (m, 4H), 7.28 – 7.13 (m, 2H), 7.11 – 7.00 (m, 2H), 6.78 – 6.75 (m, 1H), 6.65 – 6.60 (m, 1H), 6.49 – 6.46 (m, 2H), 4.87 – 4.33 (m, 2H), 4.05 (s, 3H), 3.11 (dd, *J* = 13.9, 8.4 Hz, 1H), 3.00 (dd, *J* = 14.0, 5.2 Hz, 1H).

^{13}C NMR (101 MHz, $CDCl_3$) δ 162.4, 147.4, 145.3, 143.6, 139.3, 129.2, 128.7, 127.2, 126.5, 121.2, 117.3, 117.0, 113.4, 58.9, 53.6, 39.4.

HRMS (ESI, m/z): calculated for $C_{22}H_{20}NO$ $[M+H]^+$: 305.1654, found: 305.1645.

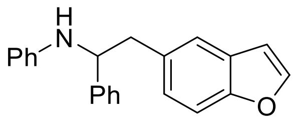

***N-(2-(benzofuran-5-yl)-1-phenylethyl)aniline* 3au.** Following the general procedure, the title compound was obtained as yellow oil using *N*-(1-phenylethyl)aniline (50 μL, 0.25 mmol) and 5-bromobenzofuran (74.5 mg, 0.38 mmol) and purified with silica gold column chromatography, eluting with a gradient (0-20%) of methanol in petroleum ether (containing 0.1% triethylamine) (44%, 34.7 mg).

^{1}H NMR (400 MHz, $CDCl_3$) δ 7.56 (d, J = 2.2 Hz, 1H), 7.36 (d, J = 8.4 Hz, 1H), 7.33 – 7.21 (m, 5H), 7.24 – 7.15 (m, 1H), 7.05 – 6.95 (m, 3H), 6.66 (dd, J = 2.2, 0.9 Hz, 1H), 6.58 (tt, J = 7.3, 1.1 Hz, 1H), 6.46 – 6.38 (m, 2H), 4.58 (dd, J = 8.2, 5.7 Hz, 1H), 4.11 (brs, 1H), 3.19 (dd, J = 14.0, 5.7 Hz, 1H), 3.05 (dd, J = 14.0, 8.2 Hz, 1H).

^{13}C NMR (101 MHz, $CDCl_3$) δ 154.1, 147.4, 145.4, 143.6, 132.2, 129.1, 128.7, 127.8, 127.2, 126.6, 125.6, 121.6, 117.6, 113.8, 111.4, 106.6, 59.7, 45.2.

HRMS (ESI, m/z): calculated for $C_{22}H_{20}NO$ $[M+H]^+$: 315.1573, found: 315.1556.

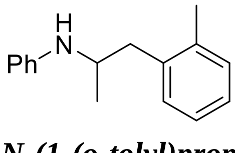

***N-(1-(o-tolyl)propan-2-yl)aniline* 3bn.** Following the general procedure with 10 mol% $Rh(PPh_3)_3Cl$ (23.0 mg) and 2 mol% $(Cy_3P)_2Pd$ (3.3 mg), the title compound was obtained as pale-yellow oil using *N*-isopropylaniline (33.8 mg, 0.25 mmol) and 1-bromo-2-methylbenzene (64.5 mg, 0.38 mmol) and purified with preparative thin layer chromatography, with hexane/toluene (v/v = 50/50) (42%, 25.3 mg).

^{1}H NMR (400 MHz, $CDCl_3$) δ 7.22 – 7.13 (m, 6H), 6.73 – 6.69 (m, 1H), 6.68 – 6.59 (m, 2H), 3.86 – 3.61 (m, 1H), 3.63 (brs, 1H), 3.02 (dd, *J* = 13.8, 5.5 Hz, 1H), 2.68 (dd, *J* = 13.8, 7.8 Hz, 1H), 2.37 (s, 3H), 1.19 (d, *J* = 6.4 Hz, 3H).

^{13}C NMR (101 MHz, $CDCl_3$) δ 147.4, 137.3, 136.6, 130.5, 130.1, 129.5, 126.5, 126.0, 117.4, 113.5, 49.2, 40.3, 20.6, 20.0.

HRMS (ESI, m/z): calculated for $C_{16}H_{20}N$ $[M+H]^+$: 226.1596, found: 226.1586.

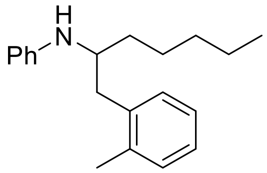

***N*-(1-(o-tolyl)heptan-2-yl)aniline** **3cn.** Following the general procedure with 10 mol% $Rh(PPh_3)_3Cl$ (23.0 mg) and 2 mol% $(Cy_3P)_2Pd$ (3.3 mg), the title compound was obtained as pale-yellow oil using *N*-(heptan-2-yl)aniline (47.8 mg, 0.25 mmol) and 1-bromo-2-methylbenzene (46 μL, 0.38 mmol) and purified with preparative thin layer chromatography, with n-hexane/toluene (v/v = 50/50) (56%, 39.4 mg).

^{1}H NMR (500 MHz, $CDCl_3$) δ 7.21 – 7.10 (m, 6H), 6.72 – 6.65 (m, 1H), 6.63 – 6.58 (m, 2H), 3.69 – 3.64 (m, 1H), 3.49 (brs, 1H), 2.91 (dd, *J* = 14.1, 6.0 Hz, 1H), 2.79 (dd, *J* = 14.1, 7.0 Hz, 1H), 2.36 (s, 3H), 1.63 – 1.57 (m, 1H), 1.54 – 1.42 (m, 2H), 1.40 – 1.33 (m, 1H), 1.31 – 1.25 (m, 4H), 0.88 (t, *J* = 6.9 Hz, 3H).

^{13}C NMR (126 MHz, $CDCl_3$) δ 147.9, 137.5, 136.6, 130.5, 130.0, 129.4, 126.4, 126.0, 117.0, 113.2, 53.6, 38.6, 34.8, 32.0, 25.9, 22.8, 20.0, 14.2.

HRMS (ESI, m/z): calculated for $C_{20}H_{28}N$ $[M+H]^+$: 282.2222, found: 282.2216.

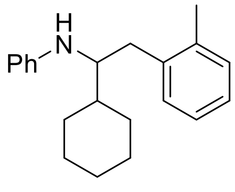

***N*-(1-cyclohexyl-2-(o-tolyl)ethyl)aniline** **3dn.** Following the general procedure with 10 mol% $Rh(PPh_3)_3Cl$ (23.0 mg) and 2 mol% $(Cy_3P)_2Pd$ (3.3 mg), the title compound was obtained as pale-yellow oil using *N*-(1-cyclohexylethyl)aniline (50.8 mg, 0.25 mmol) and 1-bromo-2-methylbenzene (46 μL, 0.38 mmol) and purified with preparative thin layer chromatography, with n-hexane/toluene (v/v = 50/50) (53%, 38.9 mg).

^{1}H NMR (400 MHz, $CDCl_3$) δ 7.19 – 7.07 (m, 6H), 6.64 – 6.60 (m, 1H), 6.54 – 6.47 (m, 2H), 3.57 – 3.53 (m, 2H), 2.92 (dd, *J* = 14.3, 5.3 Hz, 1H), 2.68 (dd, *J* = 14.3, 8.2 Hz, 1H), 2.36 (s, 3H), 1.93 – 1.57 (m, 6H), 1.38 – 1.15 (m, 5H).

^{13}C NMR (101 MHz, $CDCl_3$) δ 148.4, 138.0, 136.4, 130.4, 129.8, 129.3, 126.3, 126.0, 116.7, 113.1, 58.4, 41.4, 35.6, 29.7, 28.6, 26.8, 26.7, 26.6, 19.9.

HRMS (ESI, m/z): calculated for $C_{21}H_{28}N$ $[M+H]^+$: 294.2222, found: 294.2215.

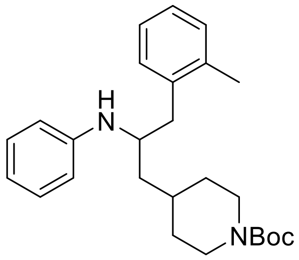

***tert*-butyl 4-(2-(phenylamino)-3-(*o*-tolyl)propyl)piperidine-1-carboxylate** **3en.** Following the general procedure, the title compound was obtained as pale-brown oil using *tert*-butyl 4-(2-(phenylamino)propyl)piperidine-1-carboxylate (79.5 mg, 0.25 mmol) and 1-bromo-2-methylbenzene (46 µL, 0.38 mmol) and purified with preparative thin layer chromatography, with ethyl acetate/toluene/diethyl ether (v/v/v = 17/33/50) (40%, 40.8 mg).

^{1}H NMR (500 MHz, $CDCl_3$) δ 7.21 – 7.09 (m, 6H), 6.72 – 6.65 (m, 1H), 6.63 – 6.58 (m, 2H), 4.02 (brs, 2H), 3.84 – 3.75 (m, 1H), 3.44 (brs, 1H), 2.96 (dd, *J* = 14.0, 5.3 Hz, 1H), 2.69 (dd, *J* = 14.0, 7.5 Hz, 1H), 2.62 – 2.60 (m, 2H), 2.32 (s, 3H), 1.67 – 1.59 (m, 3H), 1.43 (s, 9H), 1.42 – 1.39 (m, 2H), 1.20 – 1.07 (m, 1H), 1.02 – 0.91 (m, 1H).

^{13}C NMR (126 MHz, $CDCl_3$) δ 154.9, 147.6, 137.1, 136.5, 130.5, 130.0, 129.6, 126.4, 126.0, 117.3, 113.2, 79.3, 50.7, 41.9, 38.7, 33.0, 32.9, 31.8, 28.6, 20.1.

HRMS (ESI, m/z): calculated for $C_{26}H_{37}N_2O_2$ $[M+H]^+$: 409.2855, found: 409.2843.

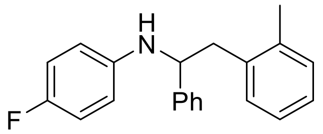

4-fluoro-N-(1-phenyl-2-(o-tolyl)ethyl)aniline **3gn.** Following the general procedure, the title compound was obtained as pale-yellow oil using 4-fluoro-*N*-(1-phenylethyl)aniline (55.8 mg, 0.25 mmol) and 1-bromo-2-methylbenzene (46 µL, 0.38 mmol) and purified with preparative thin layer chromatography, with n-hexane/toluene (v/v = 50/50) (46%, 35.1 mg).

^{1}H NMR (400 MHz, $CDCl_3$) δ 7.34 – 7.24 (m, 5H), 7.21 – 7.08 (m, 4H), 6.81 – 6.72 (m, 2H), 6.45 – 6.35 (m, 2H), 4.53 (dd, *J* = 8.5, 5.7 Hz, 1H), 4.10 (brs, 1H), 3.12 (dd, *J* = 14.3, 5.8 Hz, 1H), 3.03 (dd, *J* = 14.3, 8.5 Hz, 1H), 2.26 (s, 3H).

^{13}C NMR (101 MHz, $CDCl_3$) δ 155.9 (d, *J* = 235.0 Hz), 143.8, 143.7, 136.9, 136.1, 130.7, 129.7, 128.8, 127.3, 127.0, 126.4, 126.2, 115.5 (d, *J* = 22.2 Hz), 114.6 (d, *J* = 7.4 Hz), 59.0, 42.8, 19.6.

^{19}F NMR (376 MHz, $CDCl_3$) δ -128.9.

HRMS (ESI, m/z): calculated for $C_{21}H_{21}FN$ $[M+H]^+$: 306.1658, found: 306.1648.

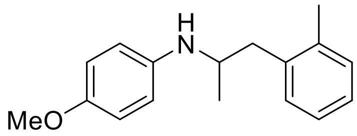

4-methoxy-N-(1-(o-tolyl)propan-2-yl)aniline **3hn.** Following the general procedure with 10 mol% $Rh(PPh_3)_3Cl$ (23.0 mg) and 2 mol% $(Cy_3P)_2Pd$ (3.3 mg), the title compound was obtained as pale-yellow oil using *N*-isopropyl-4-methoxyaniline (41.3 mg, 0.25 mmol) and 1-bromo-2-methylbenzene (46 µL, 0.38 mmol) and purified with preparative thin layer chromatography, eluting with a gradient of diethyl ether /ethyl acetate/toluene (v/v/v = 40/10/50) (50%, 31.9 mg).[12]

^{1}H NMR (400 MHz, CDCl$_3$) δ 7.15 – 7.14 (m, 4H), 6.81 – 6.78 (m, 2H), 6.63 – 6.61 (m, 2H), 3.76 (s, 3H), 3.73 – 3.63 (m, 1H), 3.13 (brs, 1H), 3.01 (dd, *J* = 13.7, 5.2 Hz, 1H), 2.63 (dd, *J* = 13.7, 7.9 Hz, 1H), 2.35 (s, 3H), 1.17 (d, *J* = 6.3 Hz, 3H).
^{13}C NMR (101 MHz, CDCl$_3$) δ 152.2, 141.7, 137.3, 136.4, 130.3, 129.9, 126.3, 125.8, 115.2, 114.9, 55.8, 50.2, 40.2, 20.5, 19.8.

7. Scale-up reaction

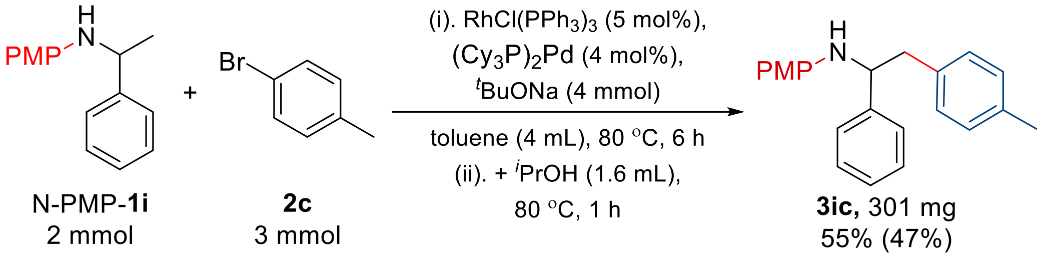

Under an inert atmosphere, N-PMP-**1i** (2 mmol, 1 equiv.),[13] 4-bromotoluene (3 mmol, 1.5 equiv.), NaO^{t}Bu (385 mg, 2 equiv.), Wilkinson's catalyst (93 mg, 5 mol%), and $(Cy_3P)_2Pd$ (53 mg, 4 mol%) were dissolved in toluene (4 mL) in a 25 mL vial equipped with a stir bar. It was then sealed, placed in a preheated oil bath, and allowed to stir at 80 °C for 6 h. The vial was allowed to cool to room temperature and ^{i}PrOH (1.6 mL) was added under an inert atmosphere. The vial was resealed, and the reaction mixture was allowed to stir for 1 h at 80 °C. After cooling to room temperature, the volatiles were removed under reduced pressure. The target product was isolated from the residue via column chromatography, eluding with a 10% diethyl ether in petroleum ether (containing 0.2% NEt_3). Fractions containing the pure product (judged by TLC and GC-MS analyses) were combined, and the solvents were removed under reduced pressure to yield the target product **3ic** (301 mg, 47%).[14]

^{1}H NMR (400 MHz, $CDCl_3$) δ 7.39 – 7.27 (m, 4H), 7.27 – 7.19 (m, 1H), 7.14 – 6.98 (m, 4H), 6.70 – 6.59 (m, 2H), 6.42 (d, *J* = 8.9 Hz, 2H), 4.49 (dd, *J* = 8.4, 5.4 Hz, 1H), 3.87 (bs, 1H), 3.68 (s, 3H), 3.10 (dd, *J* = 14.0, 5.4 Hz, 1H), 2.95 (dd, *J* = 14.0, 8.4 Hz, 1H), 2.33 (s, 3H).
^{13}C NMR (101 MHz, $CDCl_3$) δ 152.2, 144.0, 141.8, 136.3, 134.8, 129.4, 129.2, 128.7, 127.1, 126.6, 115.0, 114.8, 60.2, 55.8, 45.0, 21.2.

8. Control mechanistic experiments

Arylation of imine-1a with 2h

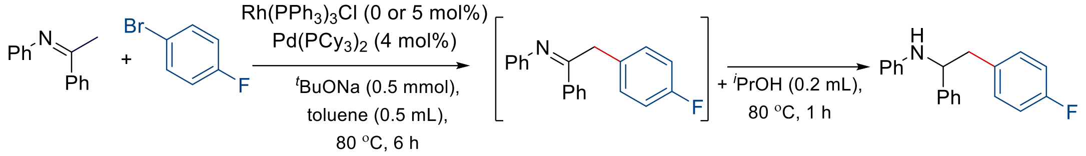

imine-1a 0.25 mmol; **2h** 0.38 mmol

imine-3ah with Rh: 69%; without Rh: 66%

3ah. with Rh: 75%; without Rh: <5%

With Rh:

- Under an inert atmosphere, a 4 mL vial equipped with a stir bar was charged with **imine-1a** (48.8 mg, 0.25 mmol, 1 equiv.), **2h** (66.5 mg, 0.38 mmol, 1.5 equiv.), NaO^{t}Bu (48.3 mg, 0.5 mmol), $Rh(PPh_3)_3Cl$ (11.6 mg, 5 mol%), $(Cy_3P)_2Pd$ (6. 7 mg, 4 mol%), and toluene (0.5 mL). It was then sealed, placed in a preheated oil bath, and allowed to stir at 80 °C for 6 h. Then, **imine-3ah** was hydrolysed to a ketone derivative to determine the NMR yield following the reported protocol:[15] After cooling to room temperature, the mixture was treated with aqueous HCl (1 M, 5 mL) and diethyl ether (5 mL). After allowing to stir at room temperature for 1 h, the separated aqueous phase was washed with ethyl acetate (2 x 5 mL). The combined organic phases were dried over $MgSO_4$ and filtered. The volatiles from the filtrate were removed under reduced pressure, 1,3,5-trimethoxybenzene (42.2 mg) was added, and the residue was subjected to the NMR analysis to determine the spectroscopic yield of hydrolysed **imine-3ah**.
- The experiment was repeated, the hydrolysis step was omitted but isopropanol (0.2 mL) was added and the mixture was allowed to stir at 80 °C for 1 h. Spectroscopic yield of **3ah** was determined following the standard procedure with 1,3,5-trimethoxybenzene as the internal standard.

Without Rh:

- Under an inert atmosphere, a 4 mL vial equipped with a stir bar was charged with **imine-1a** (48.8 mg, 0.25 mmol, 1 equiv.), **2h** (66.5 mg, 0.38 mmol, 1.5 equiv.), NaO^{t}Bu (48.3 mg, 0.5 mmol), $(Cy_3P)_2Pd$ (4 mol%, 6.67 mg), and toluene (0.5 mL). It was then sealed, placed in a preheated oil bath, and allowed to stir at 80 °C for 6 h. Then, **imine-3ah** was hydrolysed to a ketone derivative to determine the NMR yield following the reported protocol:[15] After cooling to room temperature, the mixture was treated with aqueous HCl (1 M, 5 mL) and diethyl ether (5 mL). After allowing to stir at room temperature for 1 h, the separated aqueous phase was washed with ethyl acetate (2 x 5 mL). The combined organic phases were dried over $MgSO_4$ and filtered. The volatiles from the filtrate were removed under reduced pressure, 1,3,5-trimethoxybenzene (42.2 mg) was added, and the residue was subjected to the NMR analysis to determine the spectroscopic yield of hydrolysed **imine-3ah.**

- The experiment was repeated, the hydrolysis step was omitted but isopropanol (0.2 mL) was added and the mixture was allowed to stir at 80 °C for 1 h. Spectroscopic yield of **3ah** was determined following the standard procedure with 1,3,5-trimethoxybenzene as the internal standard.

Arylation of a mixture of imine-1a and 1g with 2h

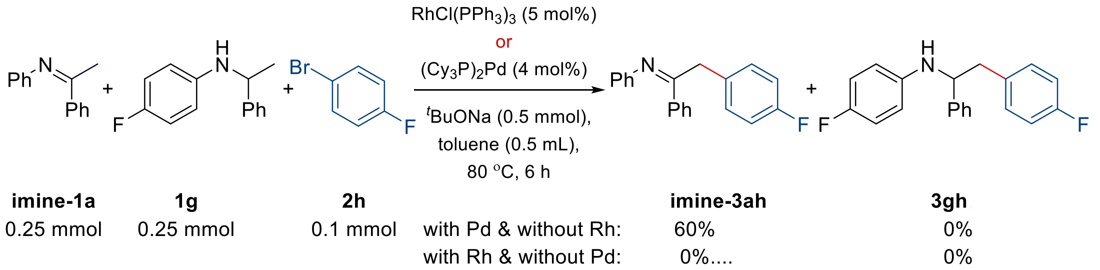

With Pd & without Rh:

Under an inert atmosphere, a 4 mL vial equipped with a stir bar was charged with **imine-1a** (48.8 mg, 0.25 mmol, 1 equiv.), **1g** (53.8 mg, 0.25 mmol, 1 equiv.), **2h** (12.5 mg, 0.1 mmol, 0.4 equiv.), NaO*t*Bu (48.3 mg, 0.5 mmol), $(Cy_3P)_2Pd$ (4 mol%, 6.67 mg), and toluene (0.5 mL). It was then sealed, placed in a preheated oil bath, and stirred at 80 °C for 6 h. 1,3,5-trimethoxybenzene (42.2 mg) was added, and the residue was subjected to the NMR analysis to determine the spectroscopic yield of **3gh** (not detected in the NMR or GCMS data). Then, **imine-3ah** was hydrolysed to a ketone derivative to determine the NMR yield following the reported protocol:[15] The mixture was treated with aqueous HCl (1 M, 5 mL) and diethyl ether (5 mL). After allowing to stir at room temperature for 1 h, the separated aqueous phase was washed with ethyl acetate (2 x 5 mL). The combined organic phases were dried over $MgSO_4$ and filtered. The volatiles from the filtrate were removed under reduced pressure, and the residue was subjected to the NMR analysis to determine the spectroscopic yield of hydrolysed **imine-3ah**.

With Rh & without Pd:

Under an inert atmosphere, a 4 mL vial equipped with a stir bar was charged with **imine-1a** (48.8 mg, 0.25 mmol, 1 equiv.), **1g** (53.8 mg, 0.25 mmol, 1 equiv.), **2h** (12.5 mg, 0.1 mmol, 0.4 equiv.), NaO*t*Bu (48.3 mg, 0.5 mmol), $Rh(PPh_3)_3Cl$ (5 mol%, 11.56 mg), and toluene (0.5 mL). It was then sealed, placed in a preheated oil bath, and stirred at 80 °C for 6 h. 1,3,5-trimethoxybenzene (42.2 mg) was added, and the residue was subjected to the NMR analysis to determine the spectroscopic yield of **3gh** (not detected in the NMR or GCMS data). Then, **imine-3ah** was hydrolysed to a ketone derivative to determine the NMR yield following the reported protocol:[15] The mixture was treated with aqueous HCl (1 M, 5 mL) and diethyl ether (5 mL). After allowing to stir at room temperature for 1 h, the separated aqueous phase was washed with ethyl acetate (2 x 5 mL). The combined organic phases were dried over $MgSO_4$ and filtered. The volatiles from the filtrate were removed under reduced pressure, and

the residue was subjected to the NMR analysis to determine the spectroscopic yield of hydrolysed **imine-3ah**.

Synthesis of deuterated substrate

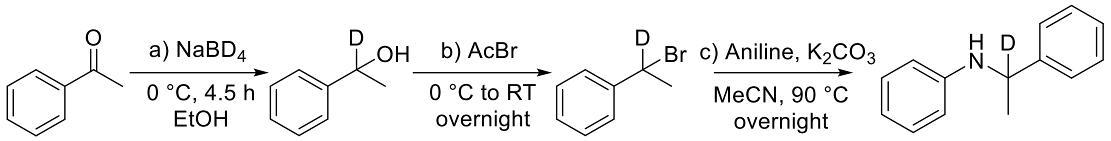

***N*-(1-phenylethyl-1-d) aniline** was synthesized according to the literature procedure.[16]

a) In a round bottom flask, acetophenone (2.34 mL, 20.0 mmol, 1 equiv.) was dissolved in EtOH (5.4 mL, 7.41 M) and cooled to 0 °C. $NaBD_4$ (268 mg, 6.40 mmol, 0.32 equiv.) was added slowly and the reaction mixture was stirred at 0 °C for 4.5 h. Then, saturated aqueous NH_4Cl solution (30 mL) was added. The aqueous phase was extracted with ethyl acetate (3x20 mL). The combined organic layers were dried over $MgSO_4$. Volatiles were removed under reduced pressure. **1-Phenylethan-1-d-1-ol** was obtained as a colorless oil (2.03 g, 16.5 mmol, 82% yield) and used without further purification.

b) Neat **1-phenylethan-1-d-1-ol** (2.03 g, 16.5 mmol, 1 equiv.) was stirred in a round bottom flask at 0 °C. Acetyl bromide (2.4 mL, 33 mmol, 2 equiv.) was added slowly. The reaction mixture was stirred overnight, allowing to warm up to room temperature. Then, water (10 mL) was added. The aqueous phase was extracted with dichloromethane (3x10 mL). The combined organic layers were dried over $MgSO_4$. Volatiles were removed under reduced pressure. **1-(1-Bromoethyl-1-d)benzene** was obtained as a slightly brownish-yellow oil (2.6 g, 14 mmol, 85% yield) and used without further purification.

c) A Teflon-capped glass vial was charged with aniline (1.6 mL, 18 mmol, 1 equiv.), **1-(1-bromoethyl-1-d)benzene** (2.6 g, 14 mmol, 0.8 equiv.), K_2CO_3 (2.9 g, 21 mmol, 1.2 equiv.) and MeCN (1 M). The reaction mixture was stirred at 90 °C overnight. Upon cooling to room temperature, the reaction mixture was diluted with ethyl acetate, and solids were filtered off. Volatiles from the filtrate were removed under reduced pressure. The product was purified via column chromatography using silica gel, eluding with a 10% diethyl ether in petroleum ether (containing 0.2% NEt_3). Fractions containing the pure product (judged by TLC) were combined, and the solvents were removed under reduced pressure to yield the target product **[α-D]-1a** (1.99 g, 72% yield, >99% D).

^{1}H NMR (400 MHz, $CDCl_3$) δ 7.43 – 7.30 (m, 4H), 7.29 – 7.21 (m, 1H), 7.15 – 7.05 (m, 2H), 6.67 (tt, *J* = 7.3, 1.1 Hz, 1H), 6.57 – 6.49 (m, 2H), 4.04 (s, 1H), 1.53 (s, 3H).
^{13}C NMR (101 MHz, $CDCl_3$) δ 147.4, 145.3, 129.2, 128.8, 127.0, 126.0, 117.4, 113.4, 53.7 – 52.7 (m), 25.0.

KIE experiments

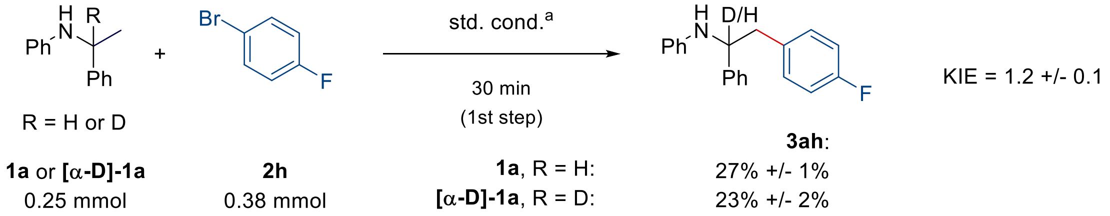

Under an inert atmosphere, a 4 mL vial equipped with a stir bar was charged with **1a** or **[α-D]-1a** (49.3 mg, 0.25 mmol, 1 equiv.), **2h** (66.5 mg, 0.38 mmol, 1.5 equiv.), NaO^{t}Bu (48.3 mg, 0.5 mmol), Rh(PPh_3)$_3$Cl (5 mol%, 11.56 mg), (Cy_3P)$_2$Pd (4 mol%, 6.67 mg) and toluene (0.5 mL). It was then sealed, placed in a preheated oil bath, and stirred at 80 °C for 30 min. The vial was allowed to cool to room temperature and ^{i}PrOH (0.2 mL) was added under inert atmosphere. The vial was resealed, and the reaction mixture was allowed to stir for at 80 °C 1 h. The vial was allowed to cool to room temperature and 1,3,5-trimethoxybenzene (42.2 mg) was added, and the residue was subjected to the NMR analysis to determine the spectroscopic yield of **3ah**.

Result:

**Vial**	Substrate	Yield%
1a	**1a**	27
1b	**1a**	26
1c	**1a**	28
2a	**D-1a**	21
2b	**D-1a**	24
2c	**D-1a**	23

9. NMR spectra

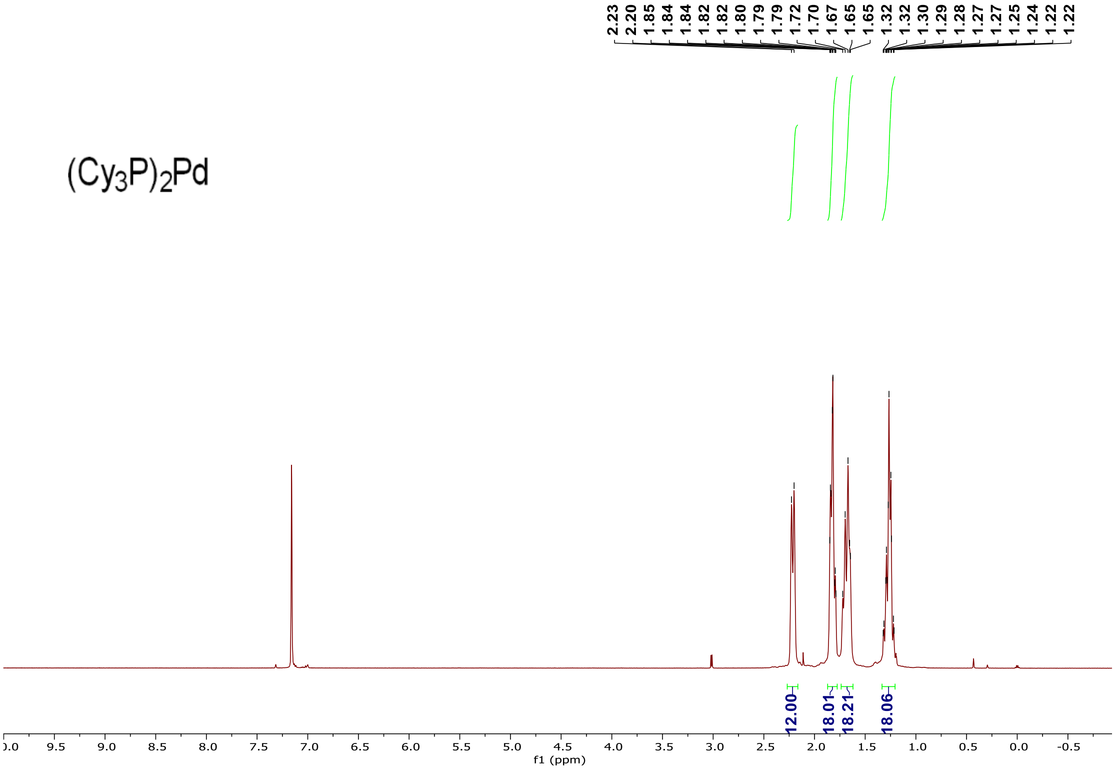

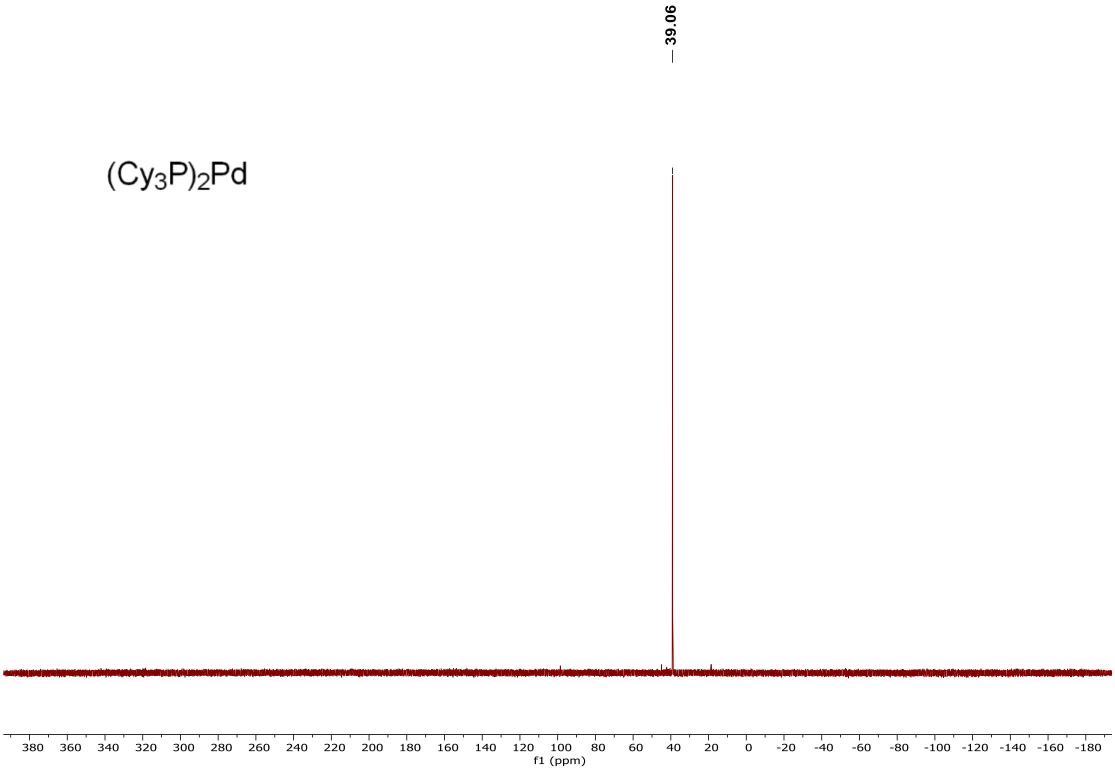

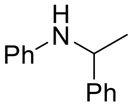
Ph
N
H
Ph

1a

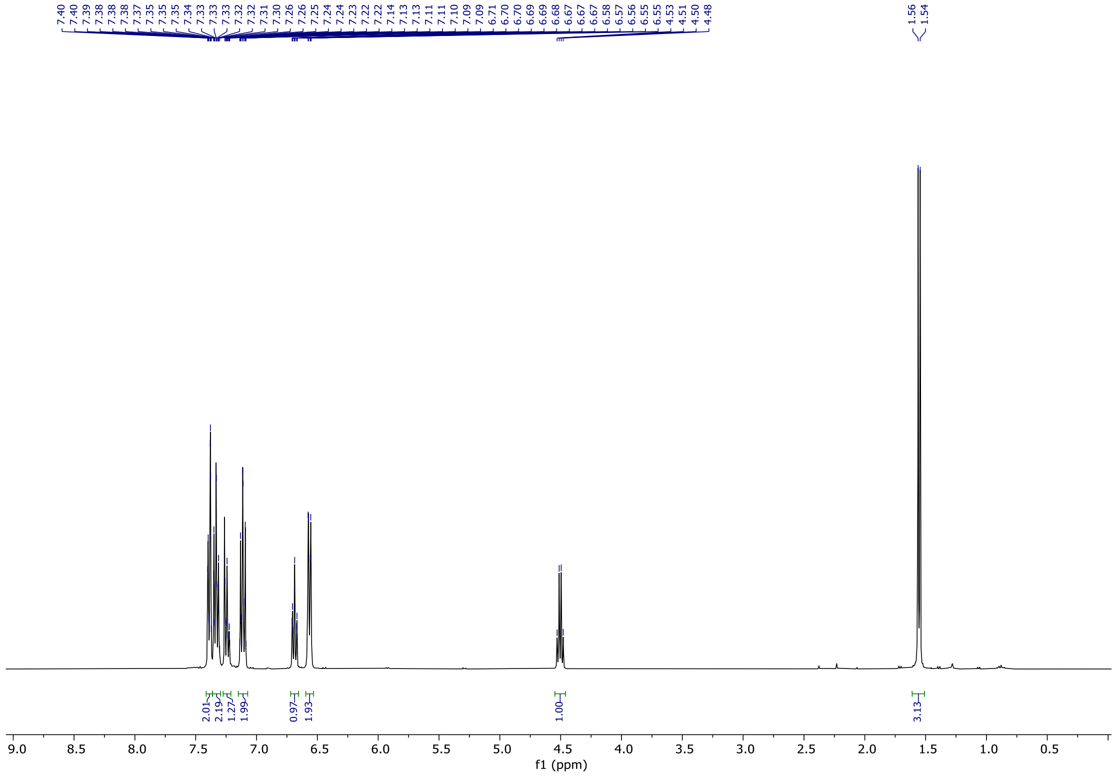
7.40
7.40
7.39
7.38
7.38
7.38
7.37
7.35
7.35
7.35
7.34
7.33
7.33
7.33
7.32
7.32
7.31
7.30
7.26
7.26
7.25
7.24
7.24
7.23
7.22
7.22
7.14
7.13
7.13
7.11
7.11
7.10
7.09
7.09
6.71
6.70
6.70
6.69
6.69
6.68
6.67
6.67
6.67
6.58
6.57
6.56
6.55
6.55
4.53
4.51
4.50
4.48
1.56
1.54
2.01
2.19
1.27
1.99
0.97
1.93
1.00
3.13
9.0
8.5
8.0
7.5
7.0
6.5
6.0
5.5
5.0
4.5
4.0
3.5
3.0
2.5
2.0
1.5
1.0
0.5
f1 (ppm)

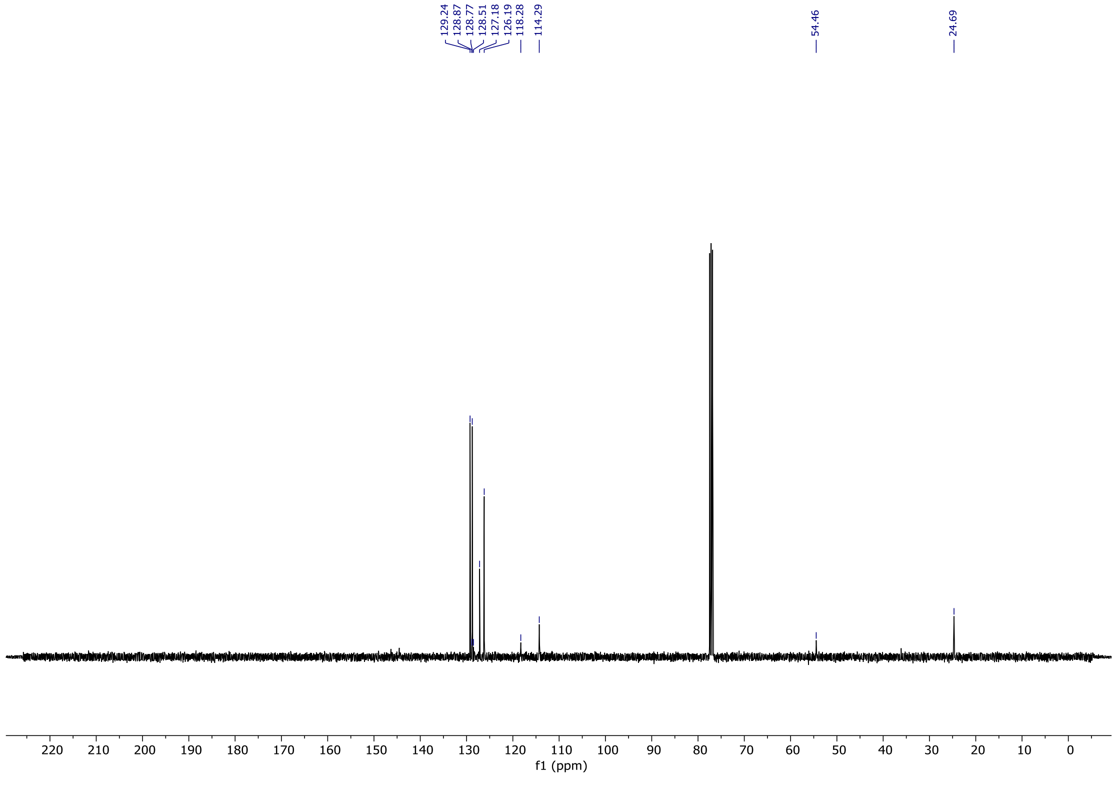
129.24
128.87
128.77
128.51
127.18
126.19
118.28
114.29
54.46
24.69
220
210
200
190
180
170
160
150
140
130
120
110
100
90
80
70
60
50
40
30
20
10
0
f1 (ppm)

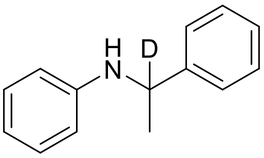
H
N
D

[α-D]-1a

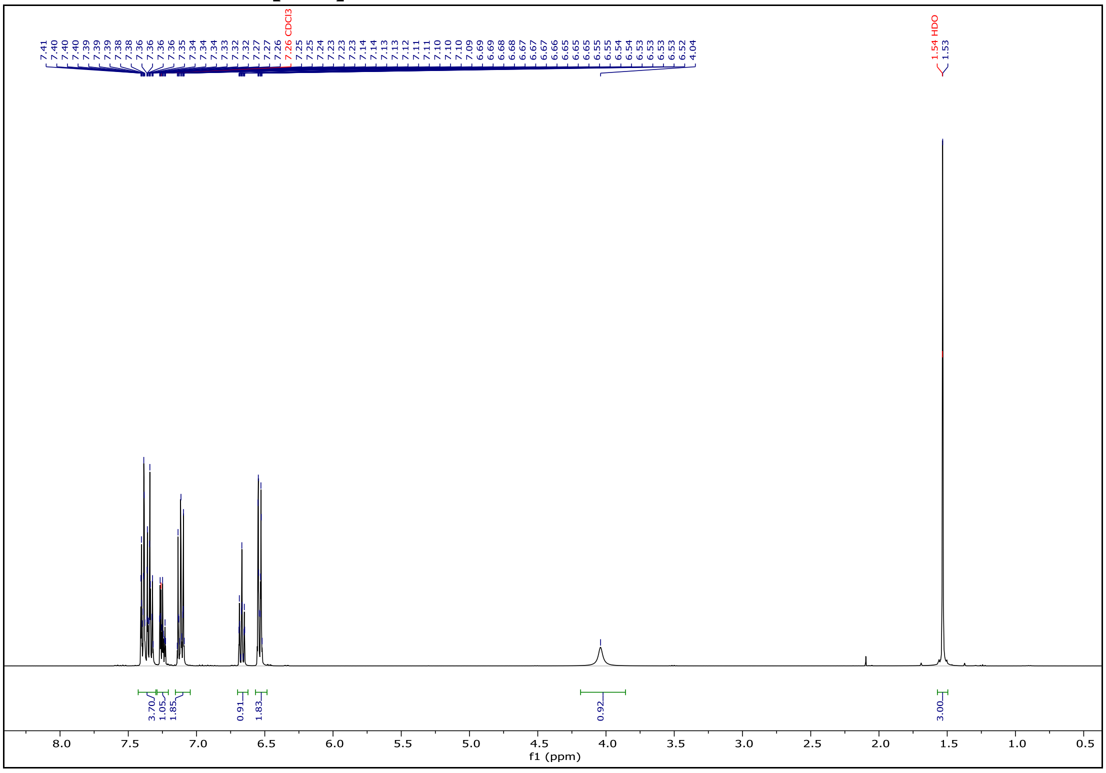
7.26 CDCl3
1.54 HDO
1.53
3.70
1.05
1.85
0.91
1.83
0.92
3.00
f1 (ppm)

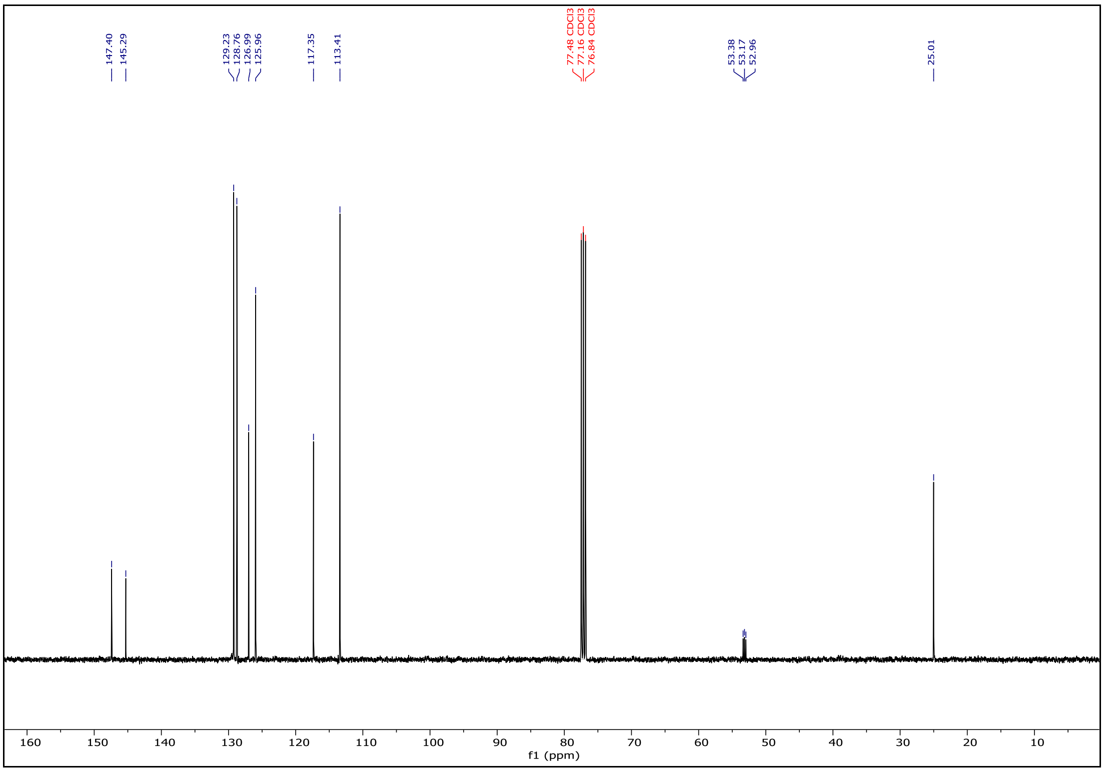
147.40
145.29
129.23
128.76
126.99
125.96
117.35
113.41
77.48 CDCl3
77.16 CDCl3
76.84 CDCl3
53.38
53.17
52.96
25.01
f1 (ppm)

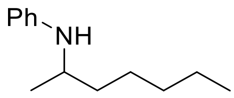
Ph
NH

1c

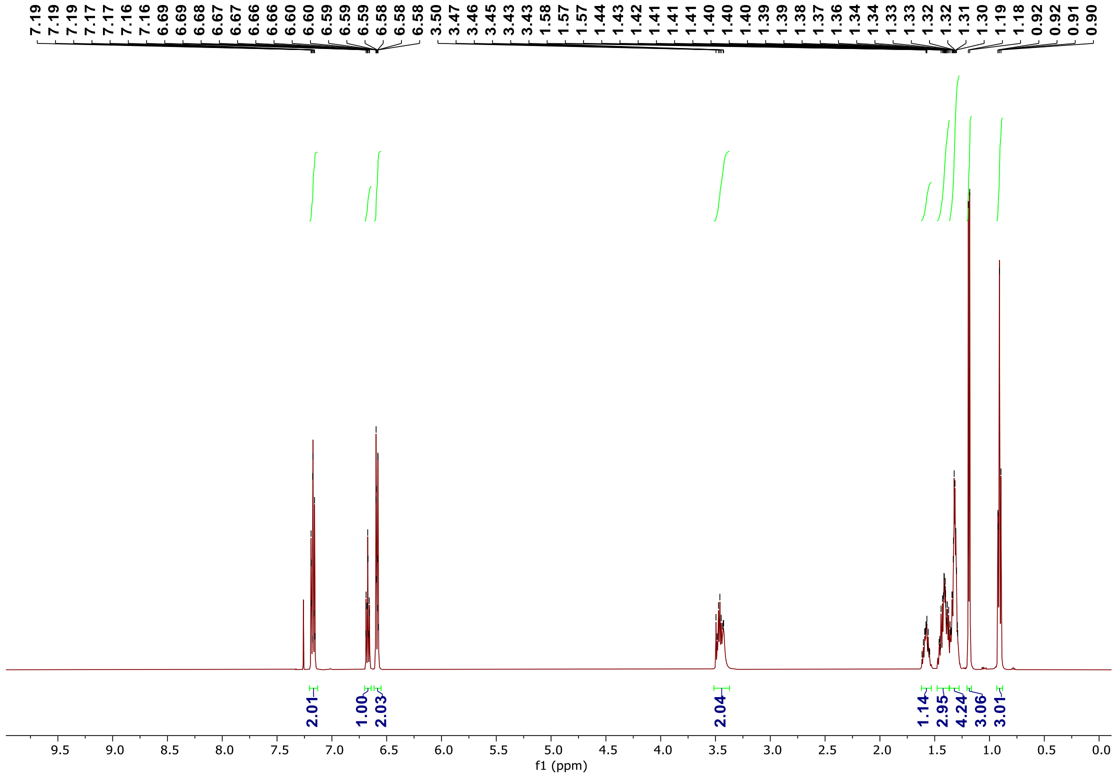
7.19
7.19
7.19
7.17
7.17
7.16
7.16
6.69
6.69
6.68
6.67
6.67
6.66
6.66
6.60
6.60
6.59
6.59
6.59
6.58
6.58
6.58
3.50
3.47
3.46
3.45
3.43
3.43
1.58
1.57
1.57
1.44
1.43
1.42
1.41
1.41
1.41
1.40
1.40
1.40
1.39
1.39
1.38
1.37
1.36
1.34
1.34
1.33
1.33
1.32
1.32
1.32
1.31
1.30
1.19
1.18
0.92
0.92
0.91
0.90
2.01
1.00
2.03
2.04
1.14
2.95
4.24
3.06
3.01
9.5 9.0 8.5 8.0 7.5 7.0 6.5 6.0 5.5 5.0 4.5 4.0 3.5 3.0 2.5 2.0 1.5 1.0 0.5 0.0
f1 (ppm)

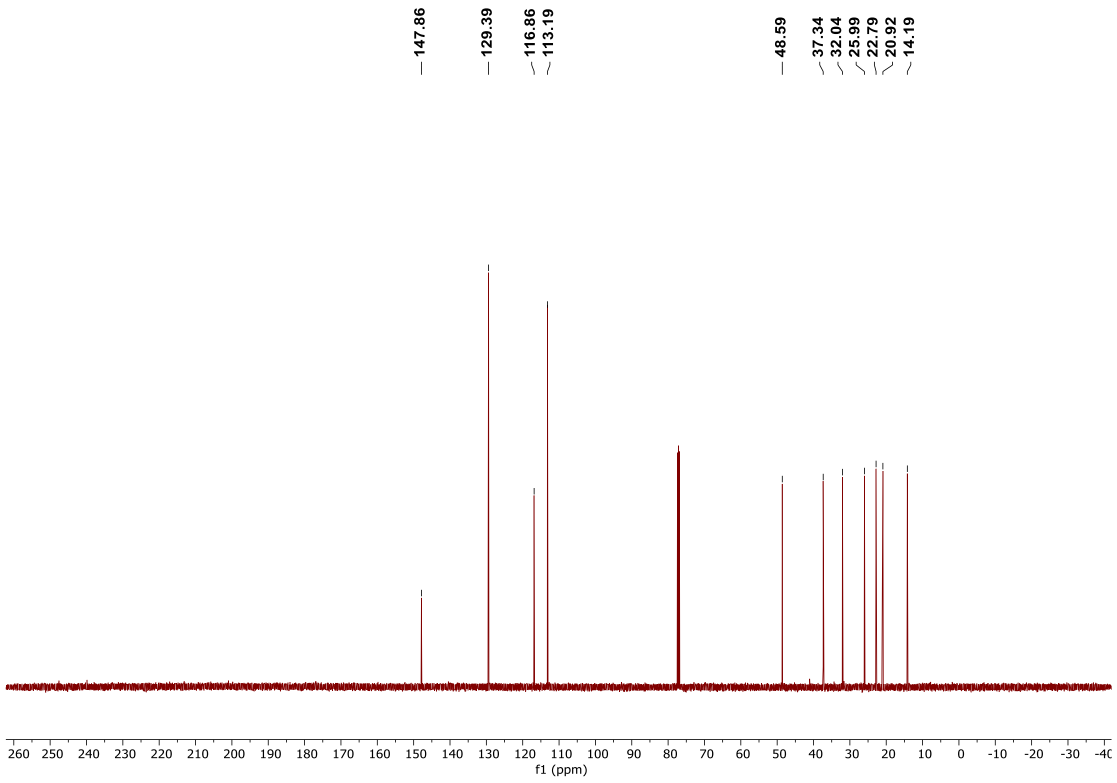
147.86
129.39
116.86
113.19
48.59
37.34
32.04
25.99
22.79
20.92
14.19
260 250 240 230 220 210 200 190 180 170 160 150 140 130 120 110 100 90 80 70 60 50 40 30 20 10 0 -10 -20 -30 -40
f1 (ppm)

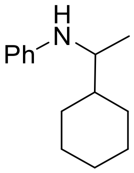
H
N
Ph

1d

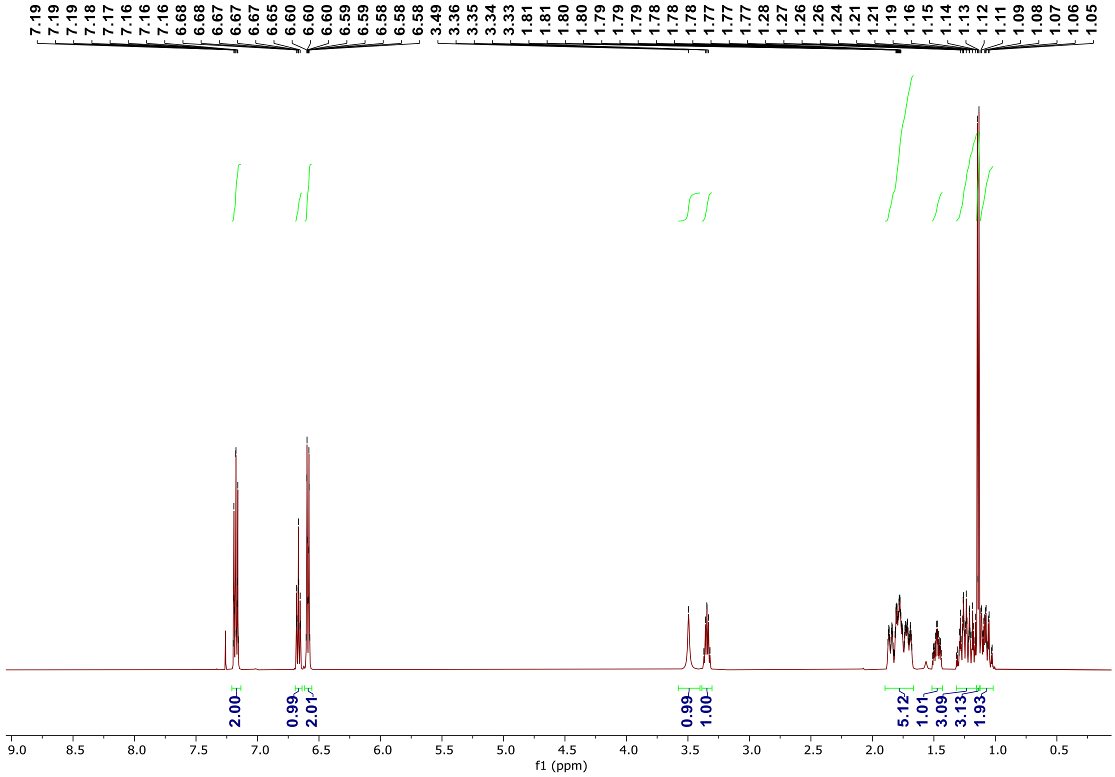
7.19
7.19
7.19
7.18
7.17
7.16
7.16
7.16
6.68
6.68
6.67
6.67
6.67
6.65
6.60
6.60
6.60
6.59
6.58
6.58
6.58
3.49
3.36
3.35
3.34
3.33
1.81
1.81
1.80
1.79
1.79
1.79
1.78
1.78
1.78
1.77
1.77
1.77
1.28
1.27
1.26
1.26
1.24
1.21
1.21
1.19
1.16
1.15
1.14
1.13
1.12
1.11
1.09
1.08
1.07
1.06
1.05
2.00
0.99
2.01
0.99
1.00
5.12
1.01
3.09
3.13
1.93
9.0 8.5 8.0 7.5 7.0 6.5 6.0 5.5 5.0 4.5 4.0 3.5 3.0 2.5 2.0 1.5 1.0 0.5
f1 (ppm)

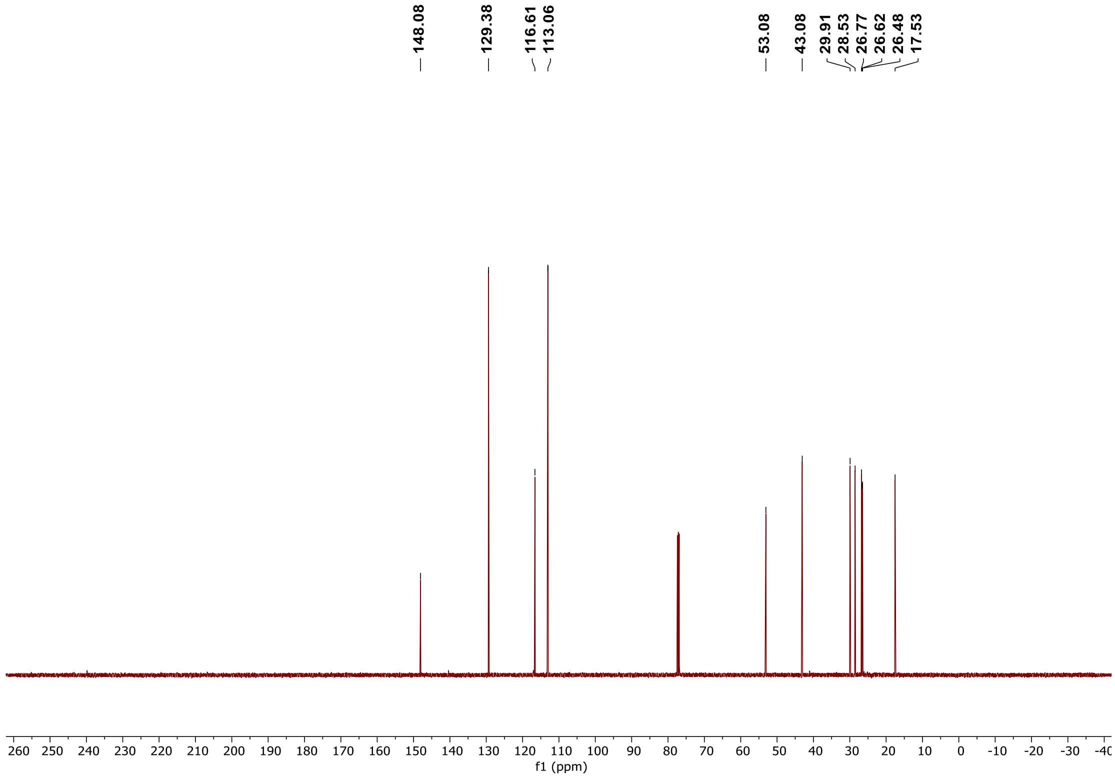
148.08
129.38
116.61
113.06
53.08
43.08
29.91
28.53
26.77
26.62
26.48
17.53
260 250 240 230 220 210 200 190 180 170 160 150 140 130 120 110 100 90 80 70 60 50 40 30 20 10 0 -10 -20 -30 -40
f1 (ppm)

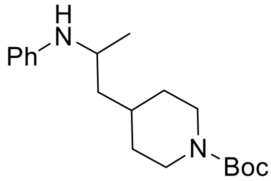

H
N
Ph
N
Boc

1e

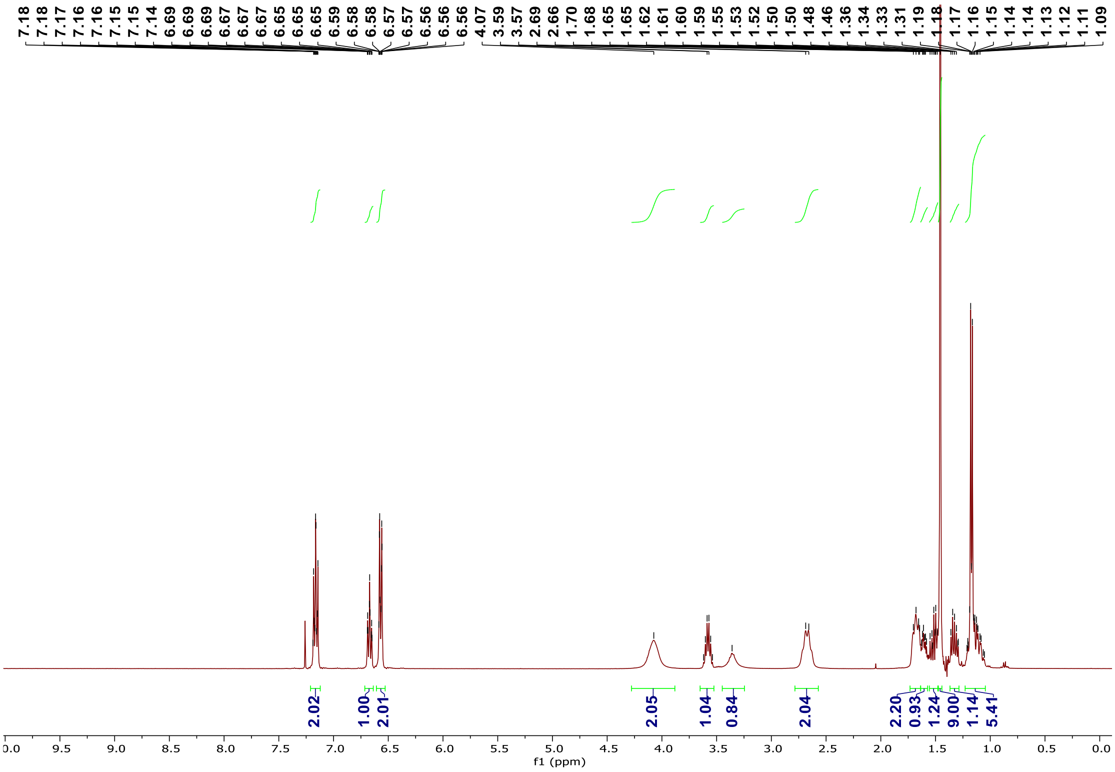

7.18 7.18 7.17 7.16 7.16 7.15 7.15 7.14 6.69 6.69 6.69 6.67 6.67 6.65 6.65 6.65 6.59 6.58 6.58 6.57 6.56 6.56 6.56 4.07 3.59 3.57 2.69 2.66 1.70 1.68 1.65 1.65 1.62 1.61 1.60 1.59 1.55 1.53 1.52 1.50 1.50 1.48 1.46 1.36 1.34 1.33 1.31 1.19 1.18 1.17 1.16 1.15 1.14 1.14 1.13 1.12 1.11 1.09
2.02 1.00 2.01 2.05 1.04 0.84 2.04 2.20 0.93 1.24 9.00 1.14 5.41
f1 (ppm)

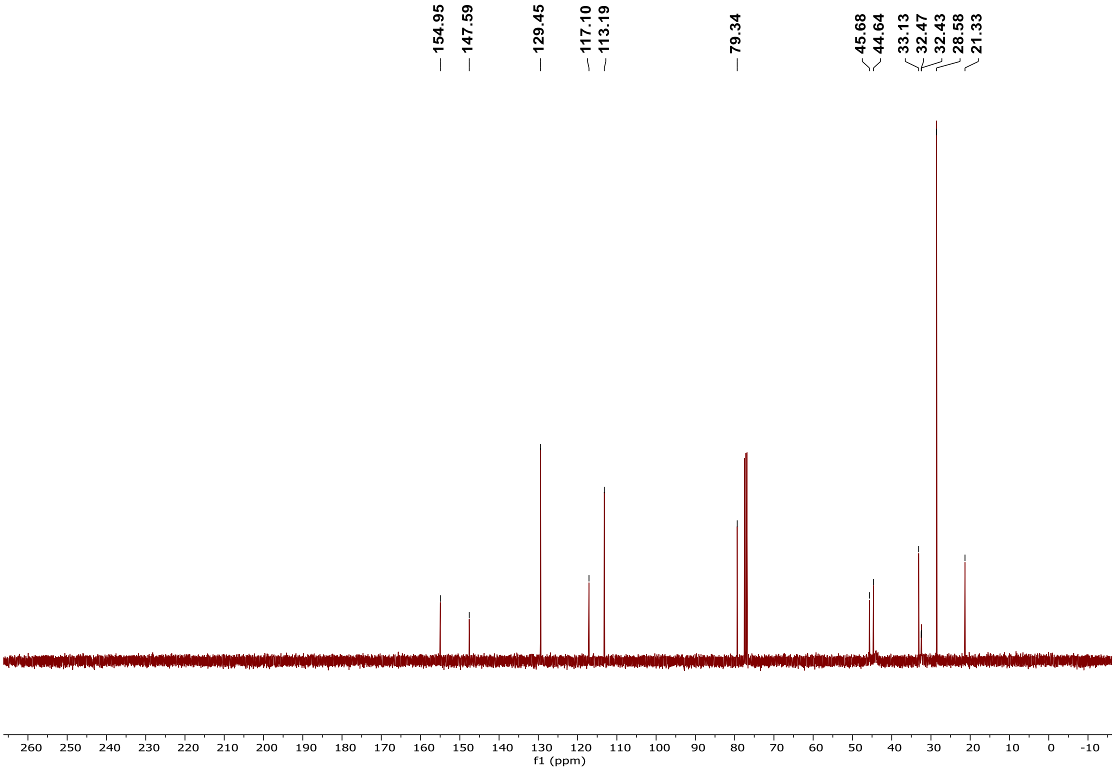

154.95 147.59 129.45 117.10 113.19 79.34 45.68 44.64 33.13 32.47 32.43 28.58 21.33
f1 (ppm)

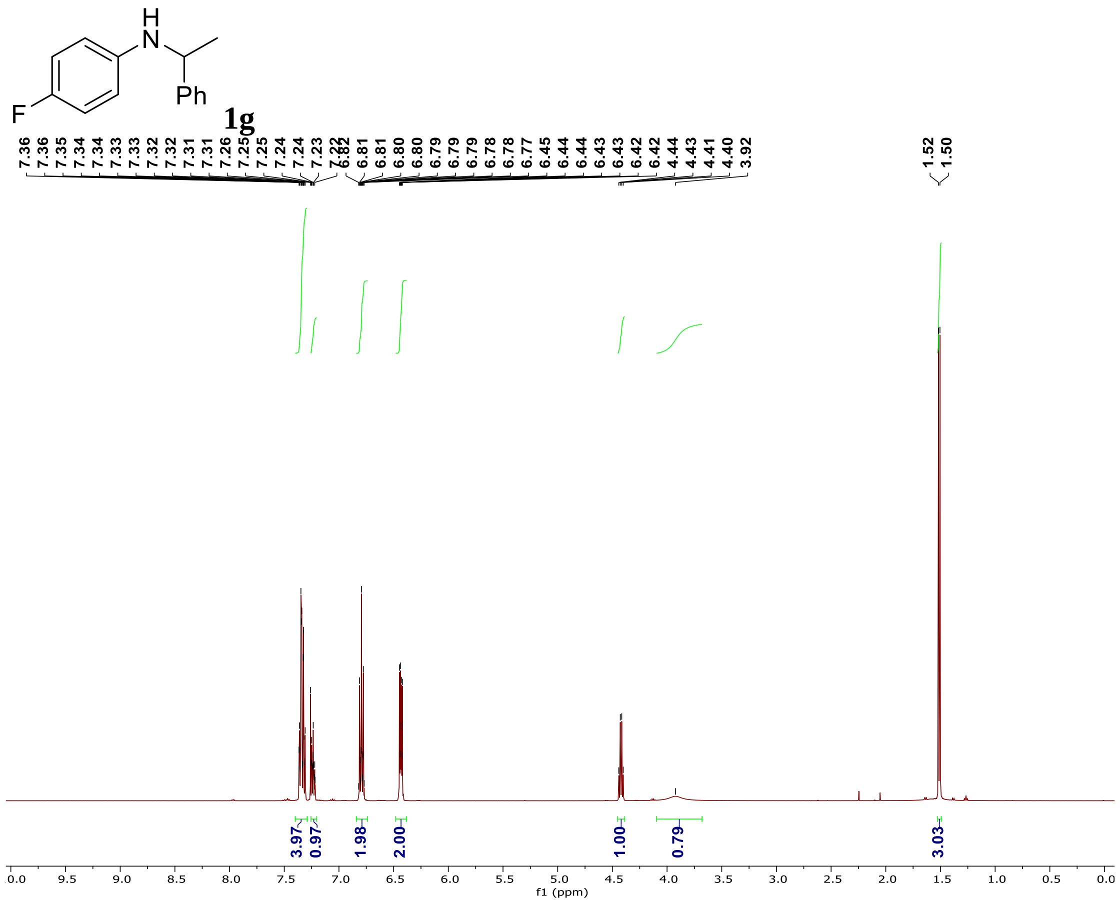

F
H
N
Ph
1g
7.36
7.36
7.35
7.34
7.34
7.33
7.33
7.32
7.32
7.31
7.31
7.26
7.25
7.25
7.24
7.24
7.23
6.81
6.81
6.80
6.80
6.79
6.79
6.79
6.78
6.78
6.77
6.45
6.44
6.44
6.43
6.43
6.42
6.42
4.44
4.43
4.41
4.40
3.92
1.52
1.50
3.97
0.97
1.98
2.00
1.00
0.79
3.03
f1 (ppm)

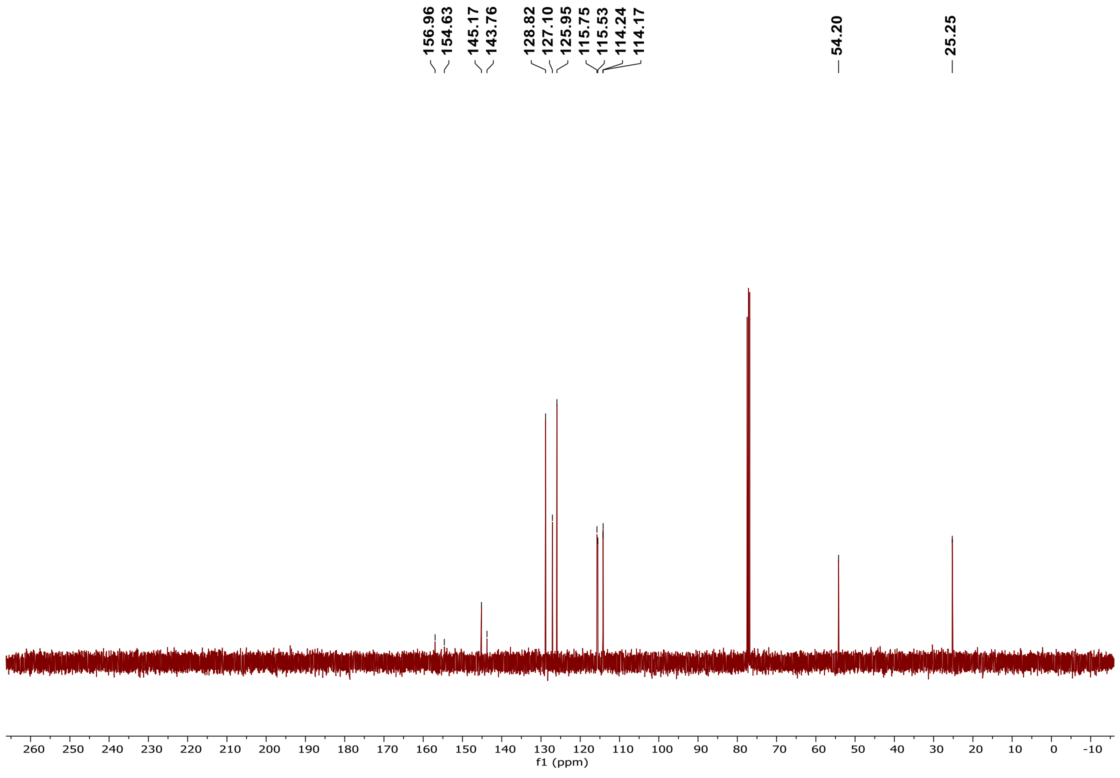

156.96
154.63
145.17
143.76
128.82
127.10
125.95
115.75
115.53
114.24
114.17
54.20
25.25
f1 (ppm)

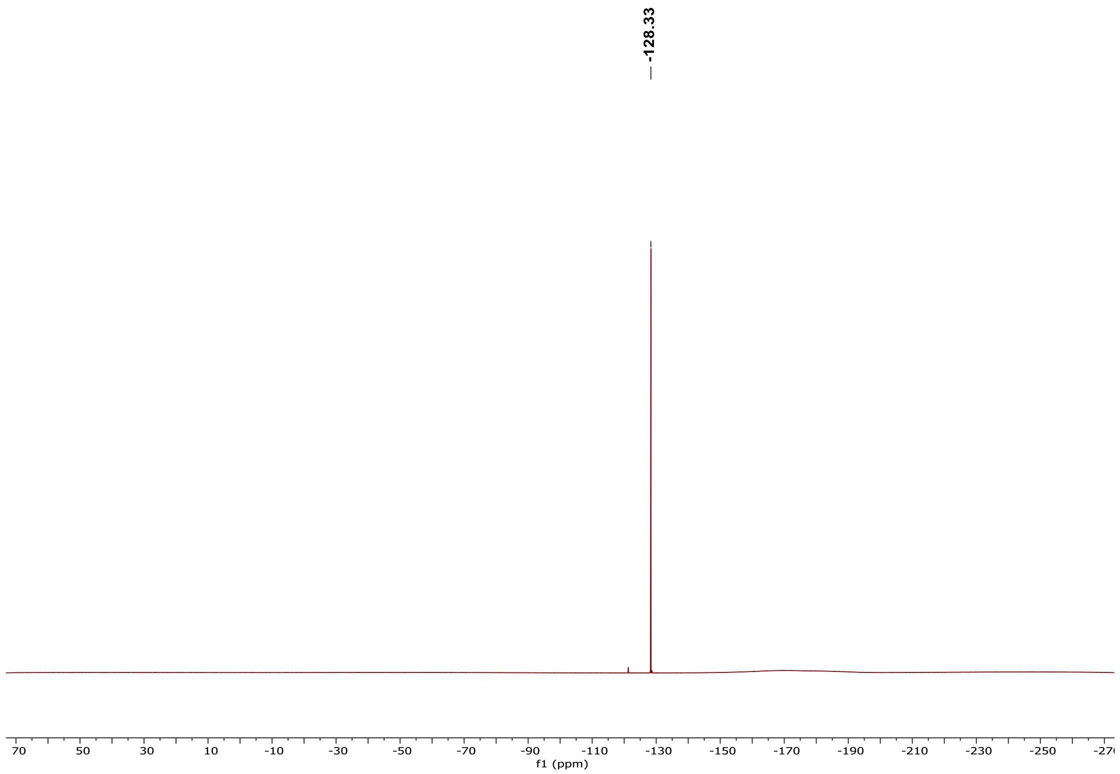
-128.33
70
50
30
10
-10
-30
-50
-70
-90
-110
-130
-150
-170
-190
-210
-230
-250
f1 (ppm)

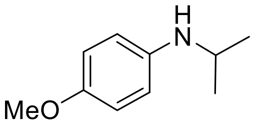

1h

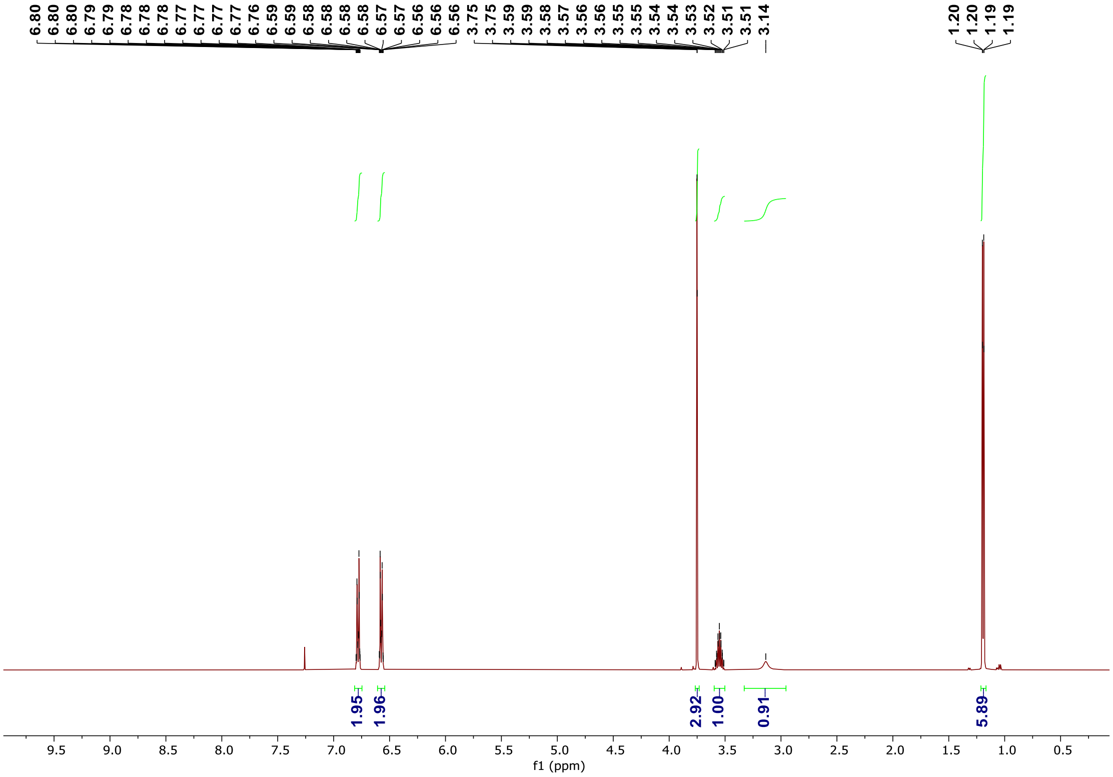

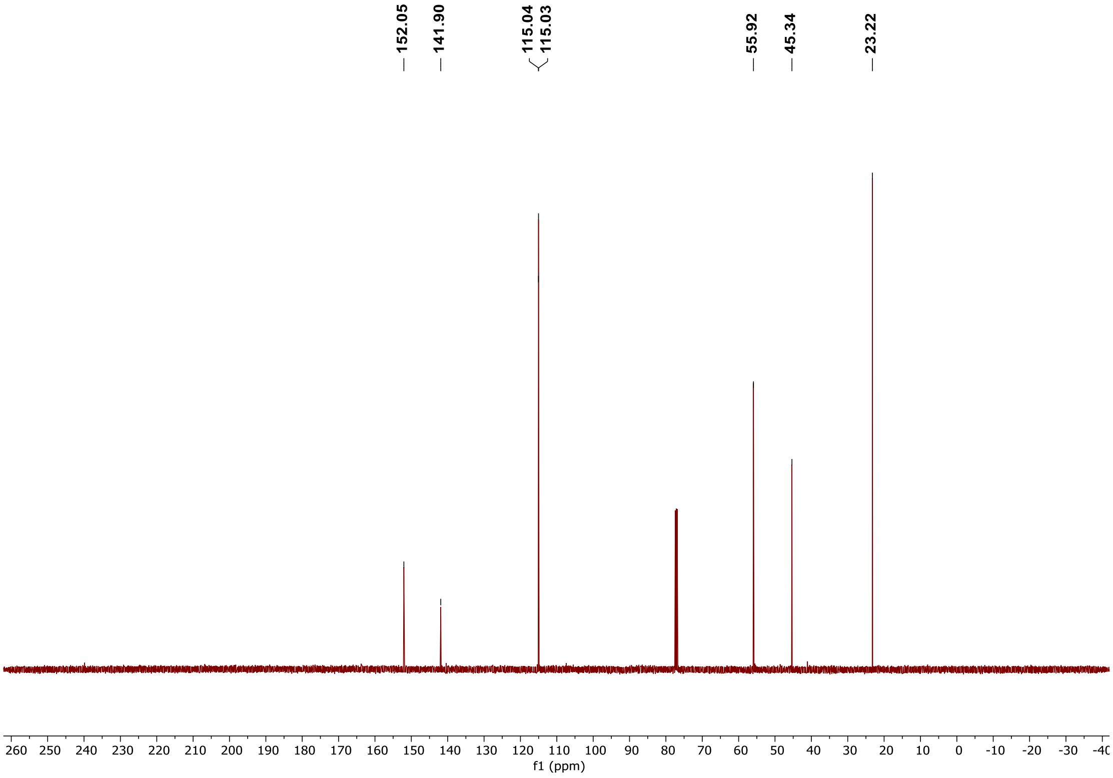

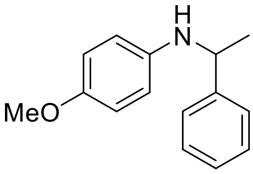

N-PMP-**1i**

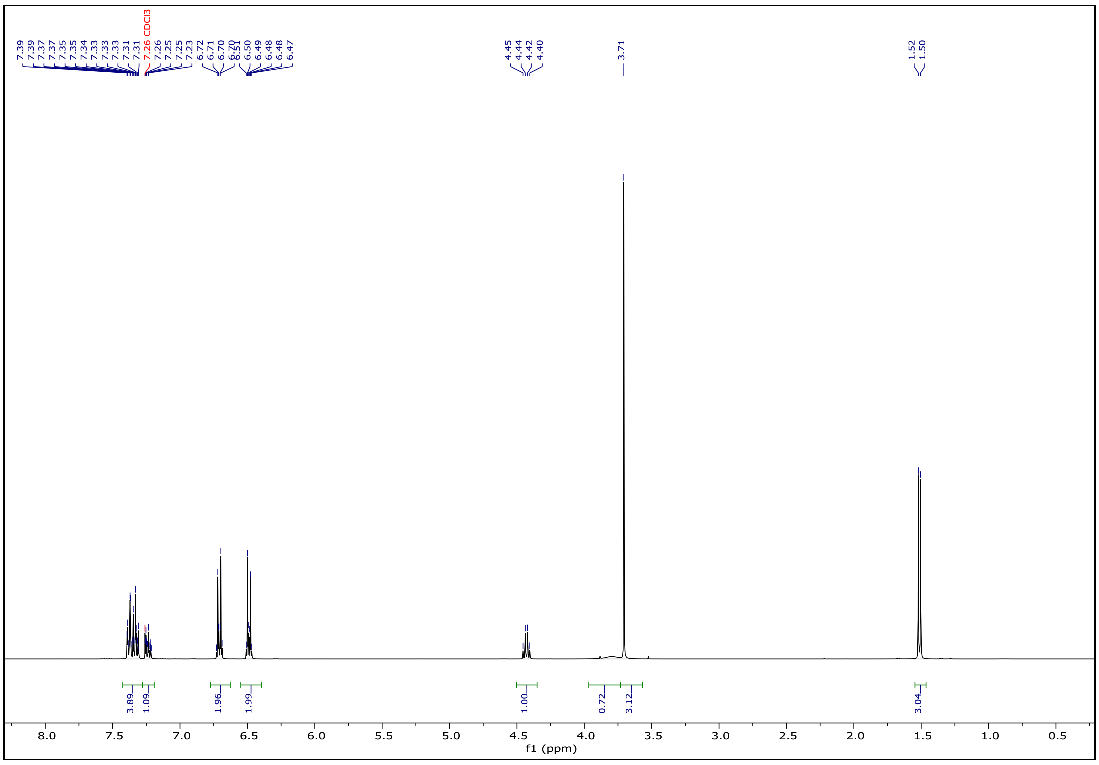

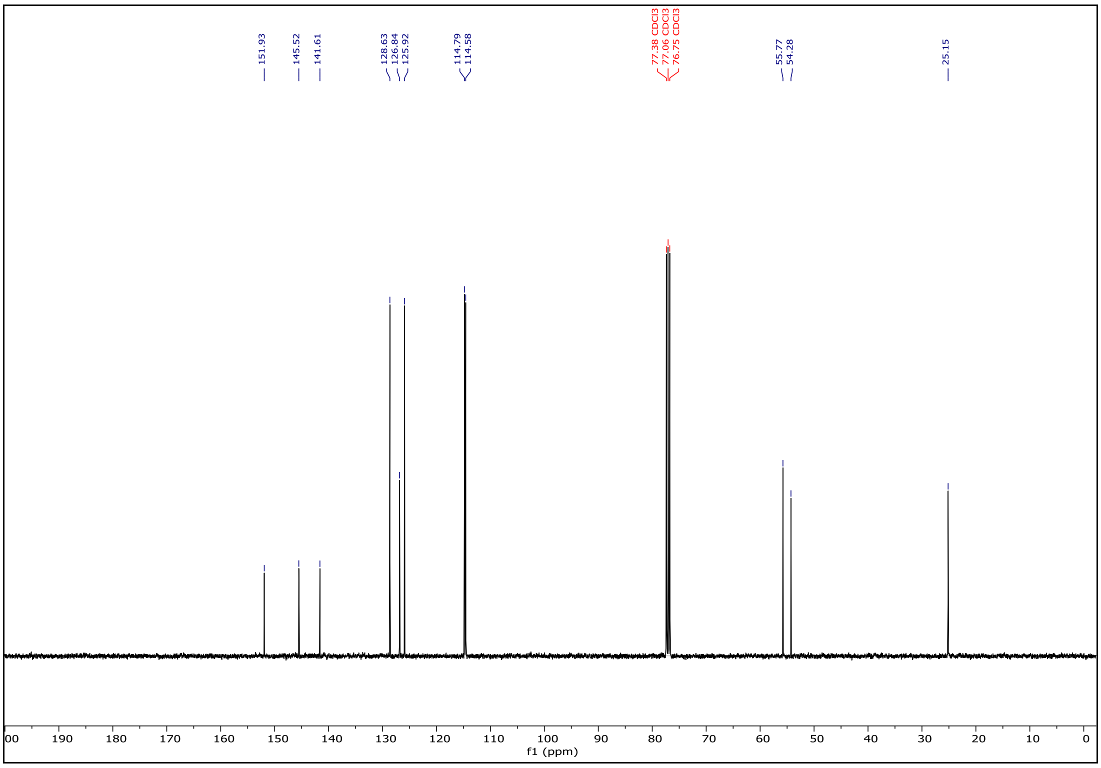

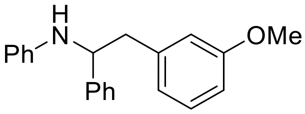
H
N
Ph
Ph
OMe

3aa

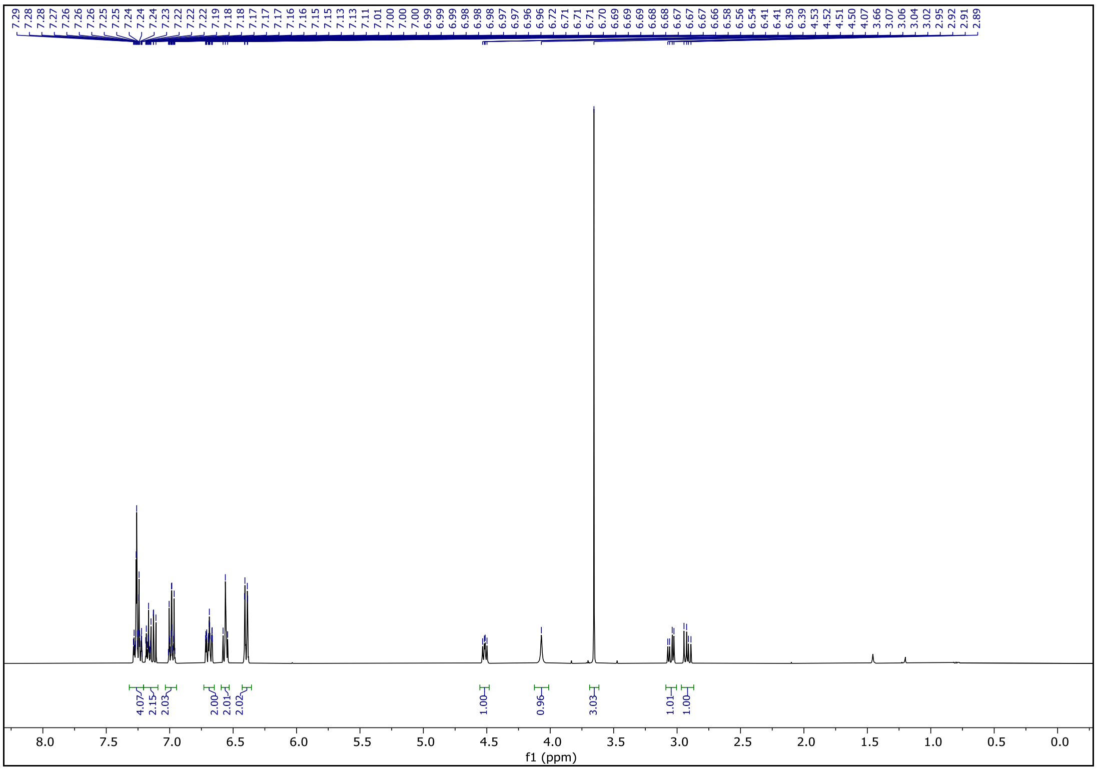
7.29 7.28 7.27 7.26 7.26 7.26 7.25 7.24 7.24 7.24 7.23 7.22 7.22 7.22 7.19 7.18 7.18 7.17 7.17 7.17 7.16 7.16 7.15 7.15 7.13 7.13 7.11 7.01 7.00 7.00 7.00 6.99 6.99 6.99 6.98 6.98 6.98 6.97 6.97 6.96 6.96 6.72 6.71 6.71 6.71 6.70 6.69 6.69 6.69 6.68 6.68 6.67 6.67 6.67 6.66 6.58 6.56 6.54 6.41 6.41 6.39 6.39 4.53 4.52 4.51 4.50 4.07 3.66 3.07 3.06 3.04 3.02 2.95 2.92 2.91 2.89
4.07
2.15
2.03
2.00
2.01
2.02
1.00
0.96
3.03
1.01
1.00
8.0 7.5 7.0 6.5 6.0 5.5 5.0 4.5 4.0 3.5 3.0 2.5 2.0 1.5 1.0 0.5 0.0
f1 (ppm)

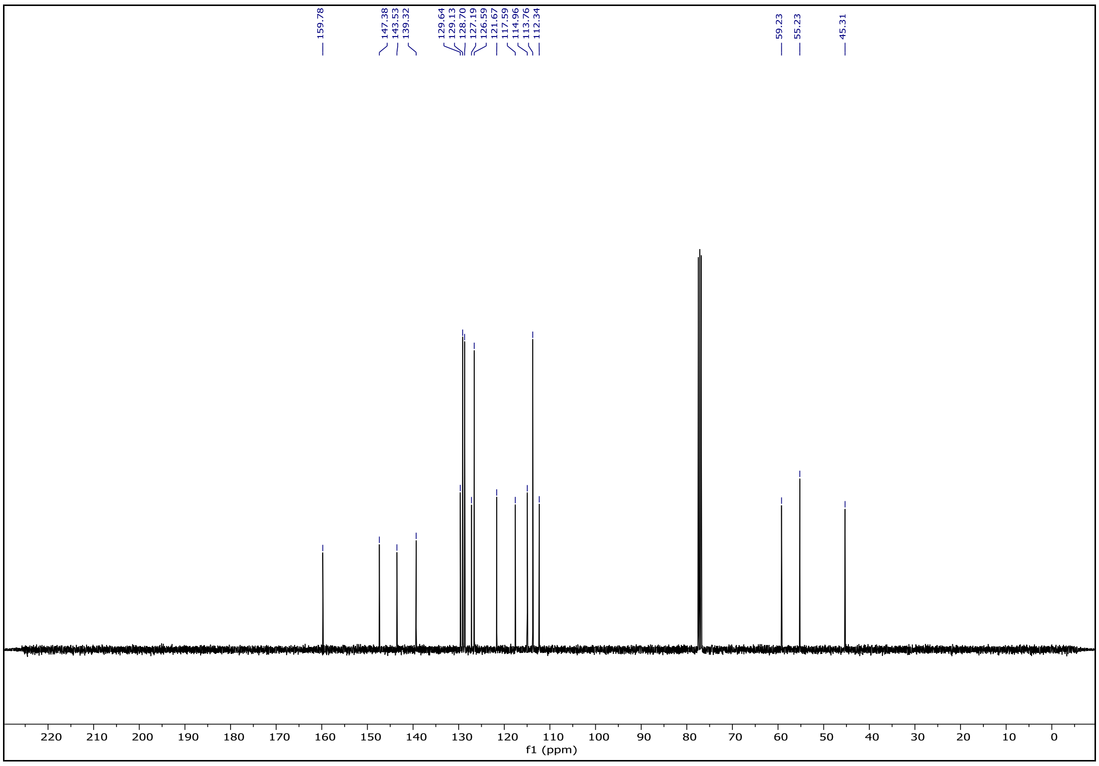
159.78
147.38
143.53
139.32
129.64
129.13
128.70
127.19
126.59
121.67
117.59
114.96
113.76
112.34
59.23
55.23
45.31
220 210 200 190 180 170 160 150 140 130 120 110 100 90 80 70 60 50 40 30 20 10 0
f1 (ppm)

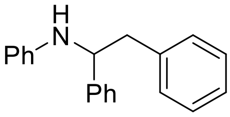

3ab

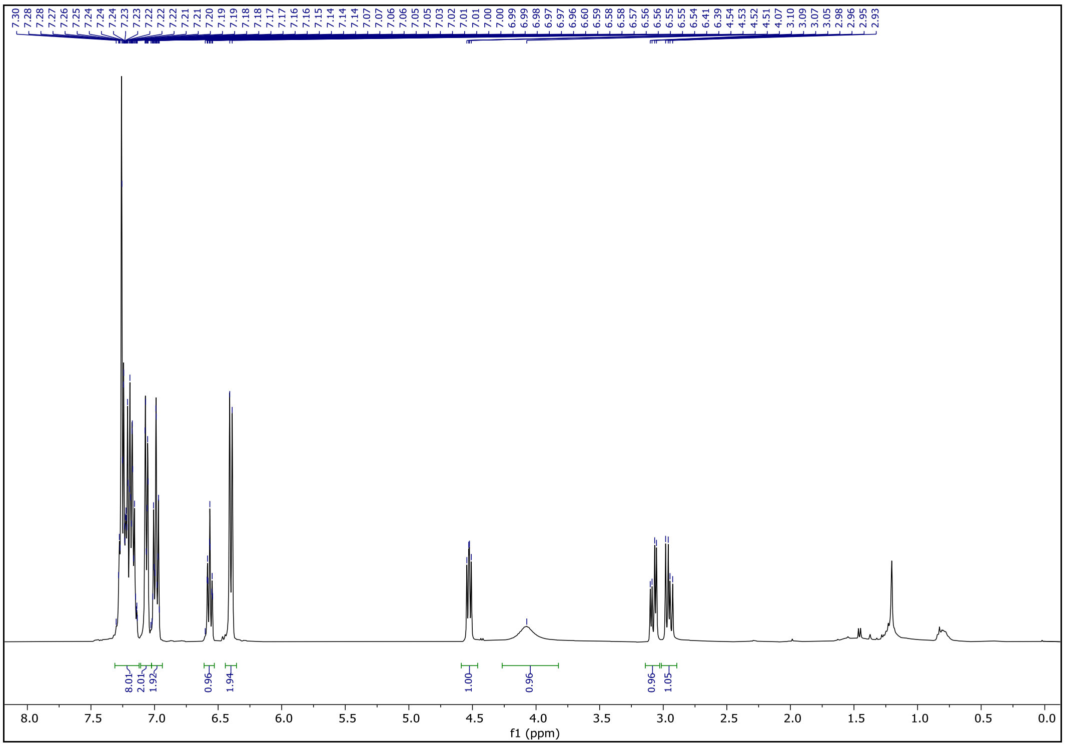

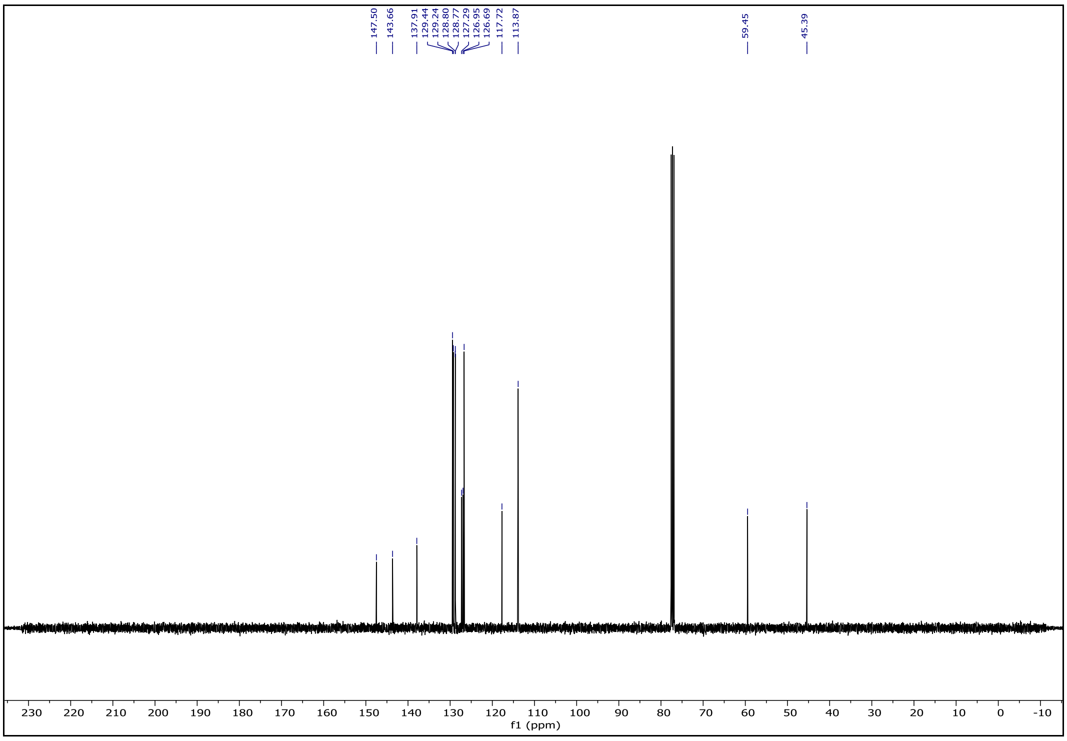

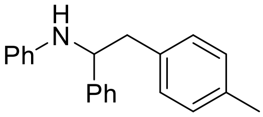

H
N
Ph
Ph

3ac

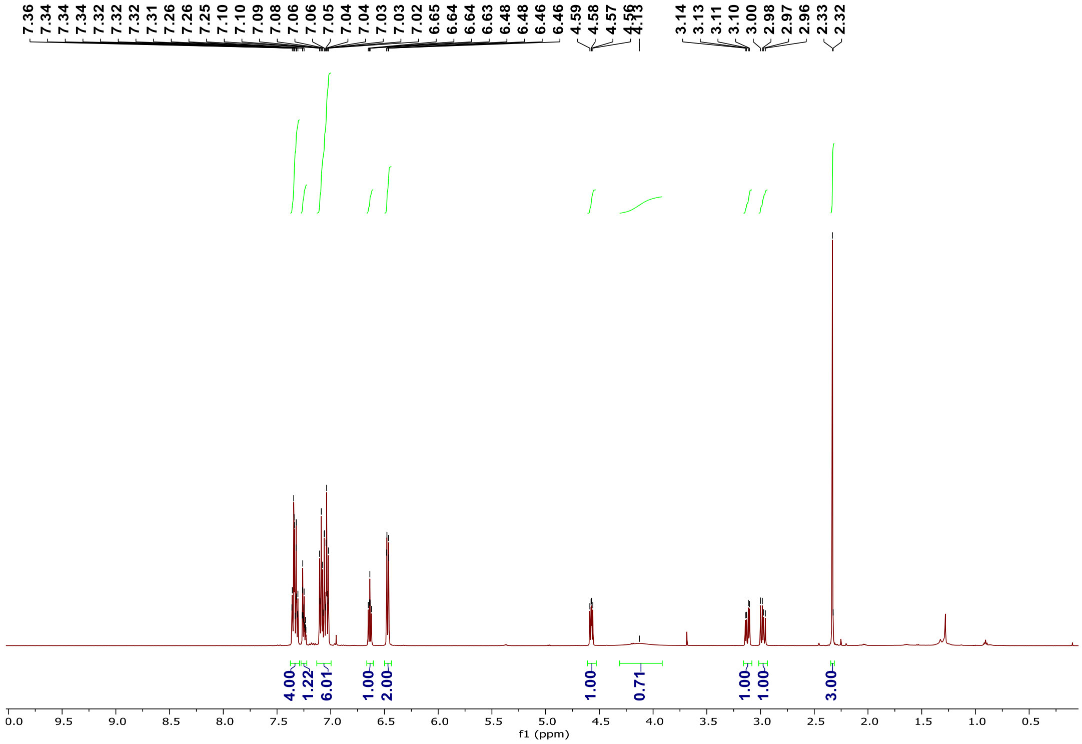

7.36 7.34 7.34 7.34 7.32 7.32 7.32 7.31 7.26 7.26 7.25 7.10 7.10 7.09 7.08 7.06 7.06 7.05 7.04 7.04 7.03 7.03 7.02 6.65 6.64 6.64 6.63 6.48 6.48 6.46 6.46
4.59 4.58 4.57 4.56 4.13
3.14 3.13 3.11 3.10 3.00 2.98 2.97 2.96 2.33 2.32
4.00 1.22 6.01 1.00 2.00 1.00 0.71 1.00 1.00 3.00
f1 (ppm)

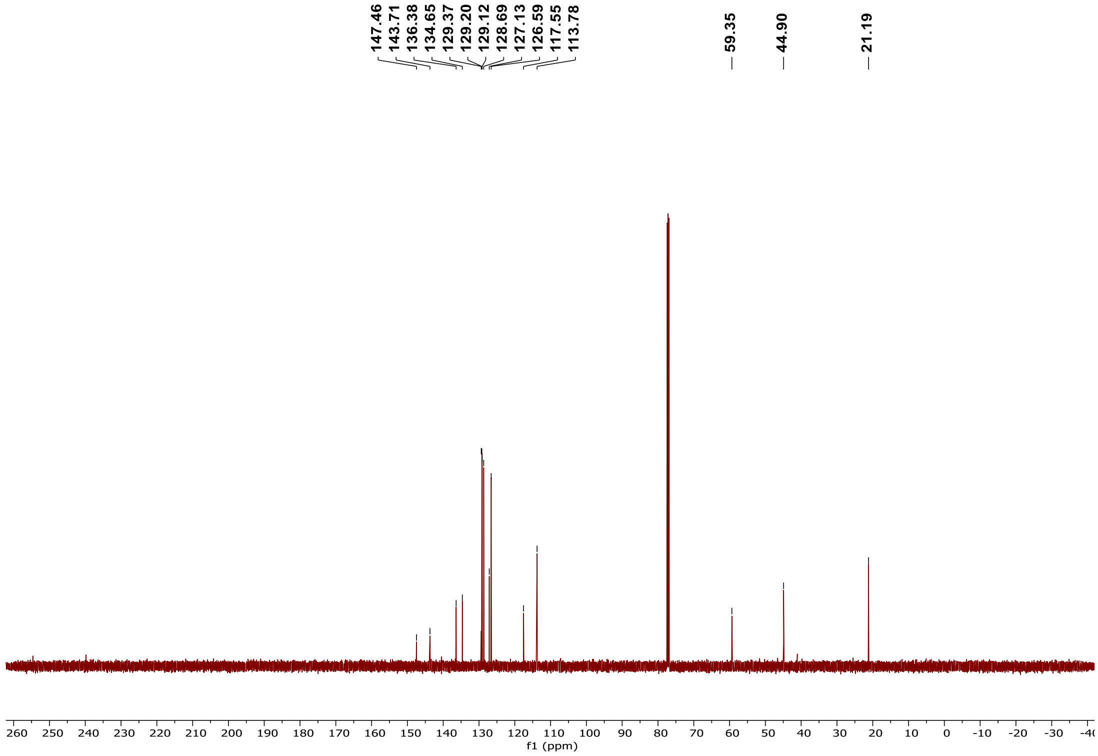

147.46 143.71 136.38 134.65 129.37 129.20 129.12 128.69 127.13 126.59 117.55 113.78
59.35
44.90
21.19
f1 (ppm)

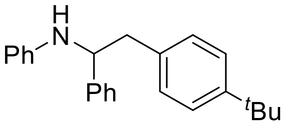
H
N
Ph
Ph
tBu

3ad

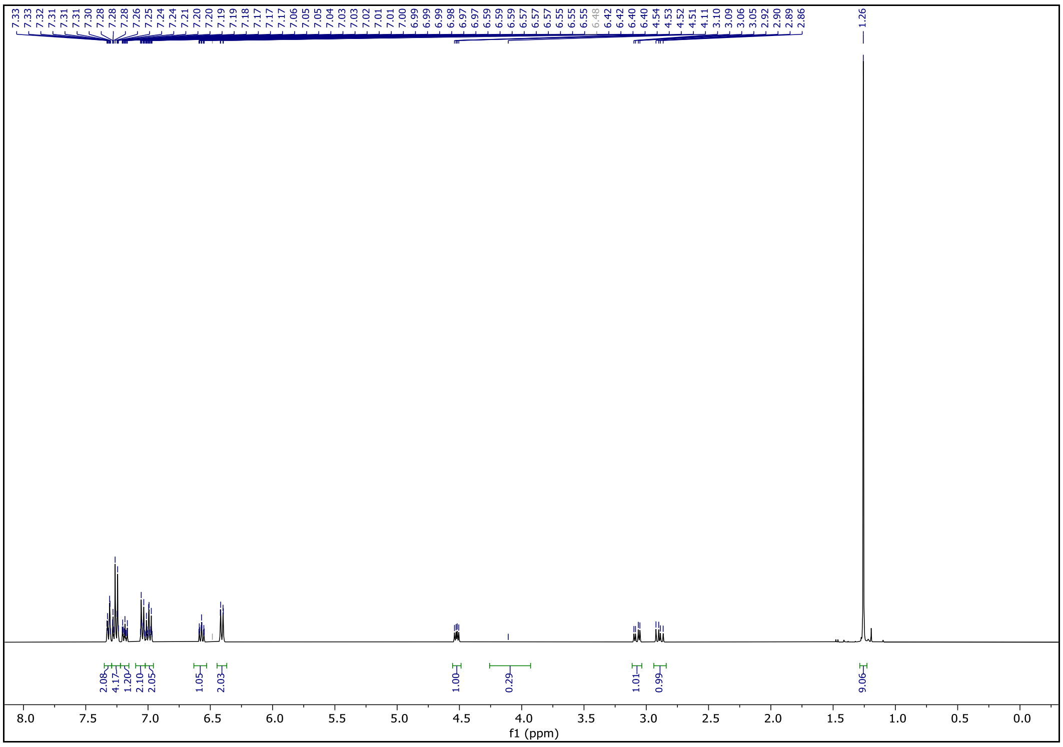
1.26
2.08
4.17
1.20
2.10
2.05
1.05
2.03
1.00
0.29
1.01
0.99
9.06
8.0 7.5 7.0 6.5 6.0 5.5 5.0 4.5 4.0 3.5 3.0 2.5 2.0 1.5 1.0 0.5 0.0
f1 (ppm)

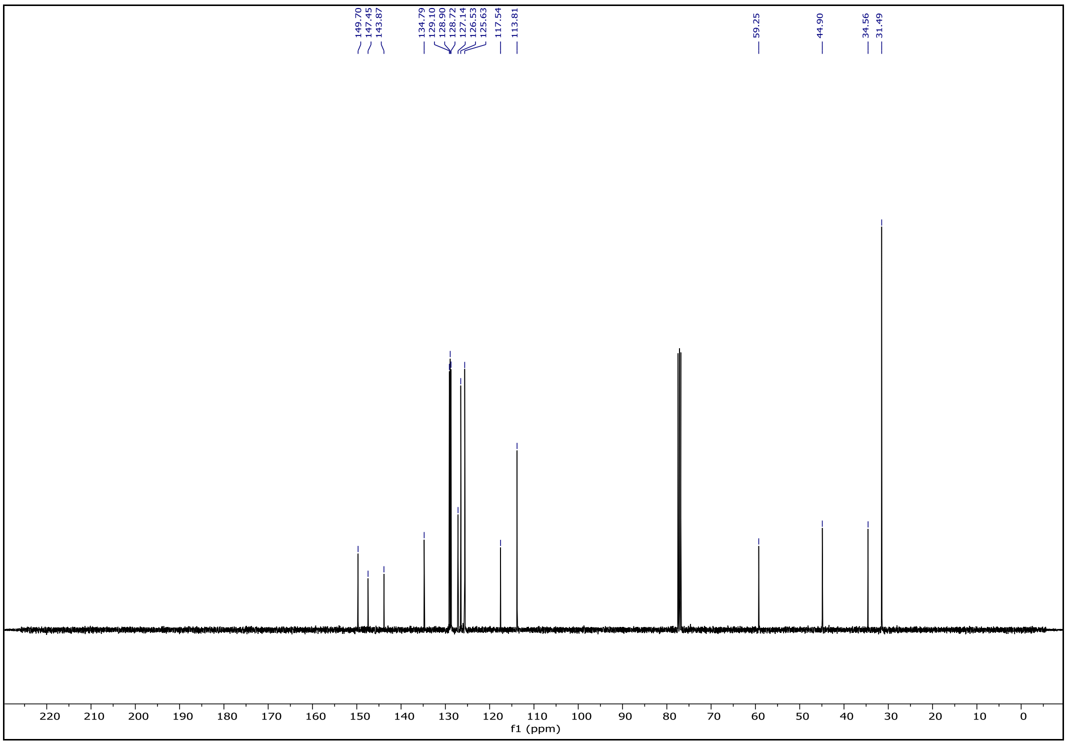
149.70
147.45
143.87
134.79
129.10
128.90
128.72
127.14
126.53
125.63
117.54
113.81
59.25
44.90
34.56
31.49
220 210 200 190 180 170 160 150 140 130 120 110 100 90 80 70 60 50 40 30 20 10 0
f1 (ppm)

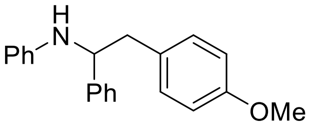

H
N
Ph
Ph
OMe

3ae

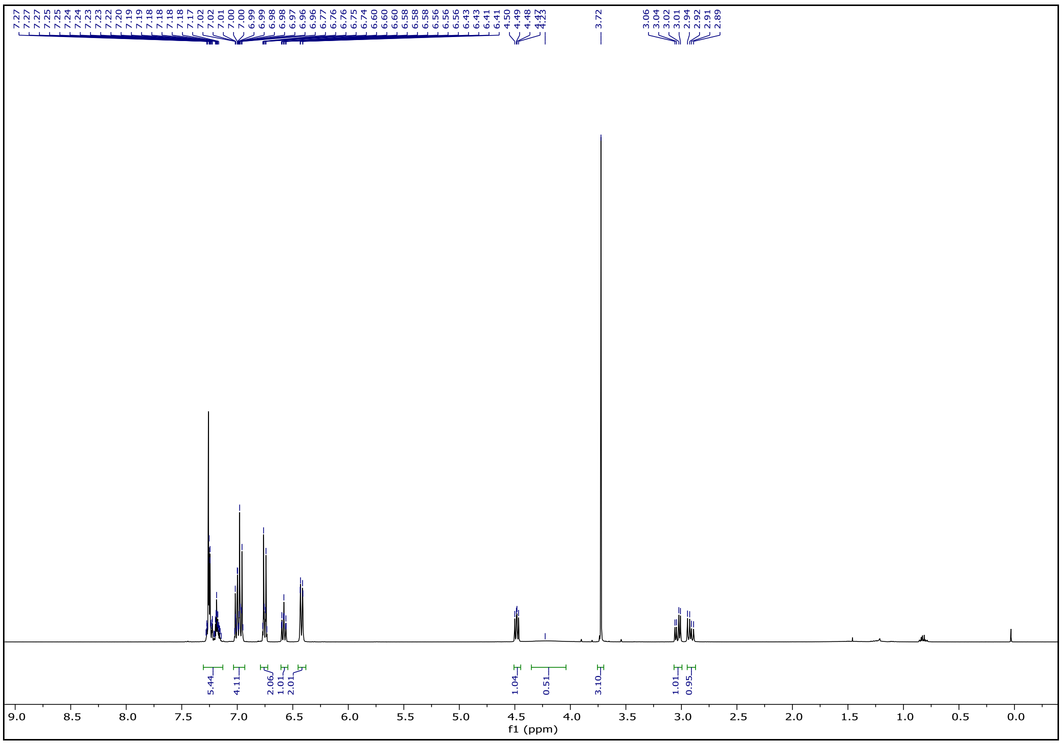

7.27 7.27 7.27 7.25 7.25 7.24 7.24 7.23 7.23 7.22 7.20 7.19 7.19 7.18 7.18 7.18 7.18 7.17 7.02 7.01 7.00 7.00 6.99 6.99 6.98 6.98 6.97 6.96 6.96 6.96 6.77 6.76 6.76 6.75 6.74 6.60 6.60 6.60 6.58 6.58 6.58 6.56 6.56 6.56 6.43 6.43 6.41 6.41 4.50 4.49 4.48 4.47 4.23 3.72 3.06 3.04 3.02 3.01 2.94 2.92 2.91 2.89
5.44 4.11 2.06 1.01 2.01 1.04 0.51 3.10 1.01 0.95
9.0 8.5 8.0 7.5 7.0 6.5 6.0 5.5 5.0 4.5 4.0 3.5 3.0 2.5 2.0 1.5 1.0 0.5 0.0
f1 (ppm)

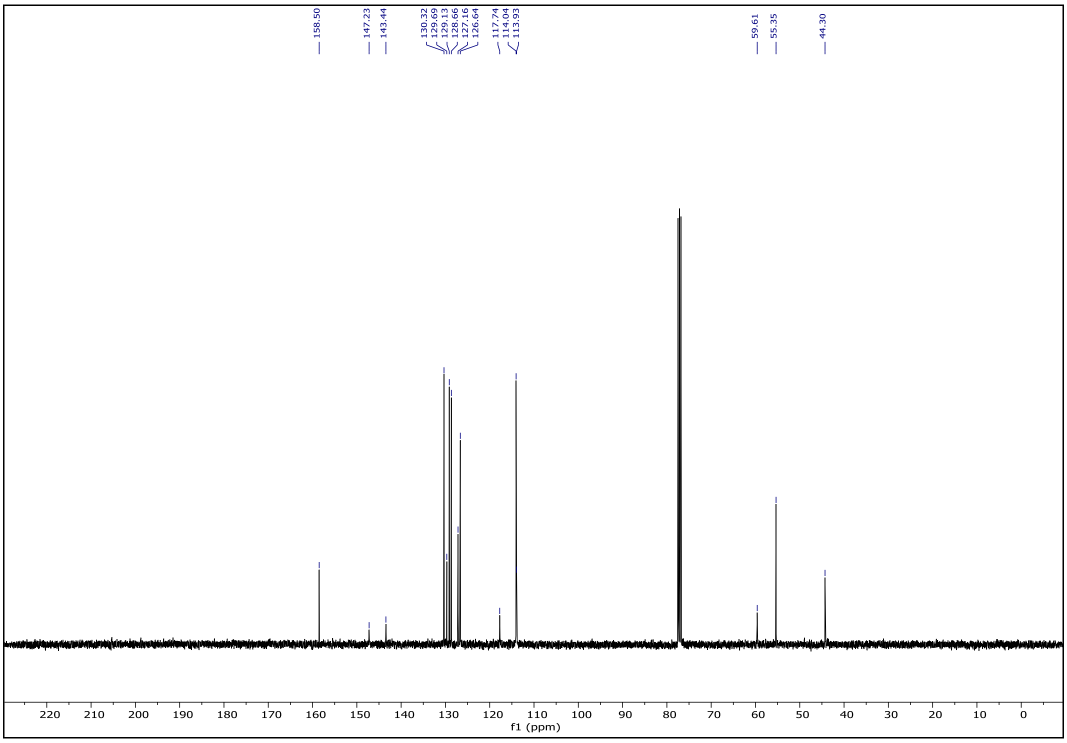

158.50 147.23 143.44 130.32 129.69 129.13 128.66 127.16 126.64 117.74 114.04 113.93 59.61 55.35 44.30
220 210 200 190 180 170 160 150 140 130 120 110 100 90 80 70 60 50 40 30 20 10 0
f1 (ppm)

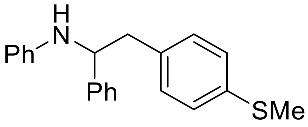

H
N
Ph
Ph
SMe

3af

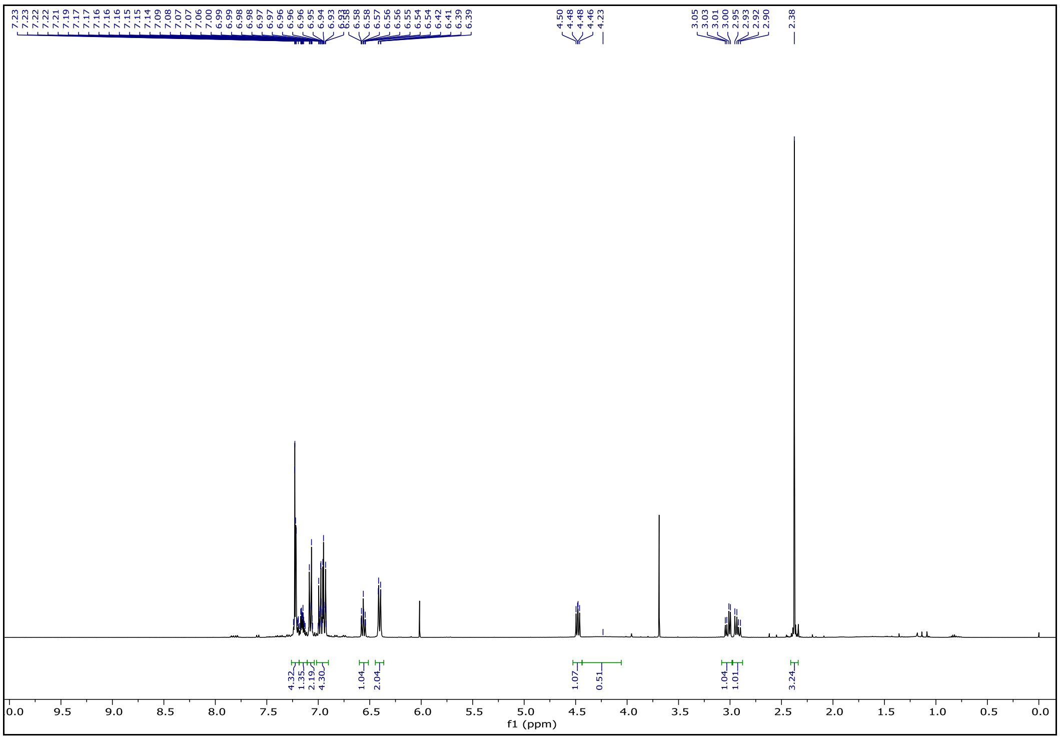

4.50
4.48
4.48
4.46
4.23
3.05
3.03
3.01
3.00
2.95
2.93
2.92
2.90
2.38
4.32
1.35
2.19
4.30
1.04
2.04
1.07
0.51
1.04
1.01
3.24
f1 (ppm)

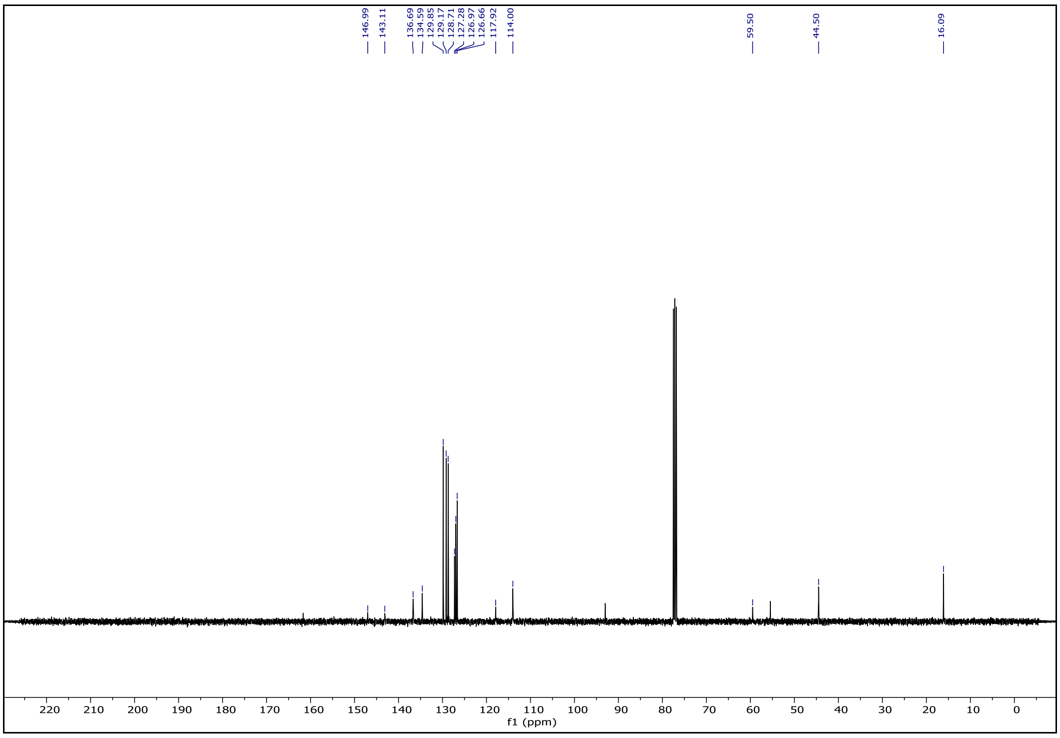

146.99
143.11
136.69
134.59
129.85
129.17
128.71
127.28
126.97
126.66
117.92
114.00
59.50
44.50
16.09
f1 (ppm)

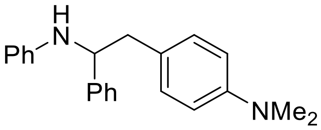
H
N
Ph
Ph
NMe2

3ag

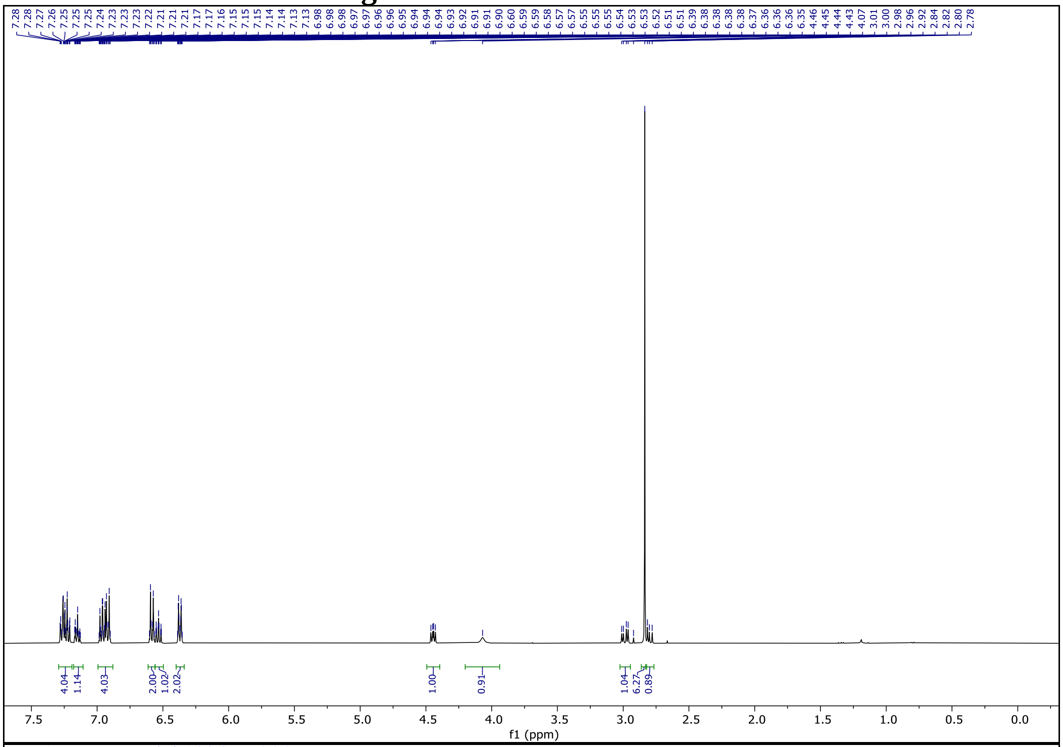
7.28 7.28 7.27 7.26 7.25 7.25 7.25 7.24 7.23 7.23 7.23 7.22 7.21 7.21 7.21 7.17 7.16 7.15 7.15 7.15 7.14 7.14 7.13 7.13 6.98 6.98 6.98 6.97 6.97 6.96 6.96 6.95 6.94 6.94 6.94 6.93 6.92 6.91 6.91 6.90 6.60 6.59 6.59 6.58 6.57 6.55 6.55 6.55 6.54 6.53 6.53 6.52 6.51 6.51 6.39 6.38 6.38 6.38 6.38 6.37 6.36 6.36 6.35 4.46 4.45 4.44 4.43 4.07 3.01 3.00 2.98 2.96 2.92 2.84 2.82 2.80 2.78
4.04
1.14
4.03
2.00
1.02
2.02
1.00
0.91
1.04
6.27
0.89
7.5 7.0 6.5 6.0 5.5 5.0 4.5 4.0 3.5 3.0 2.5 2.0 1.5 1.0 0.5 0.0
f1 (ppm)

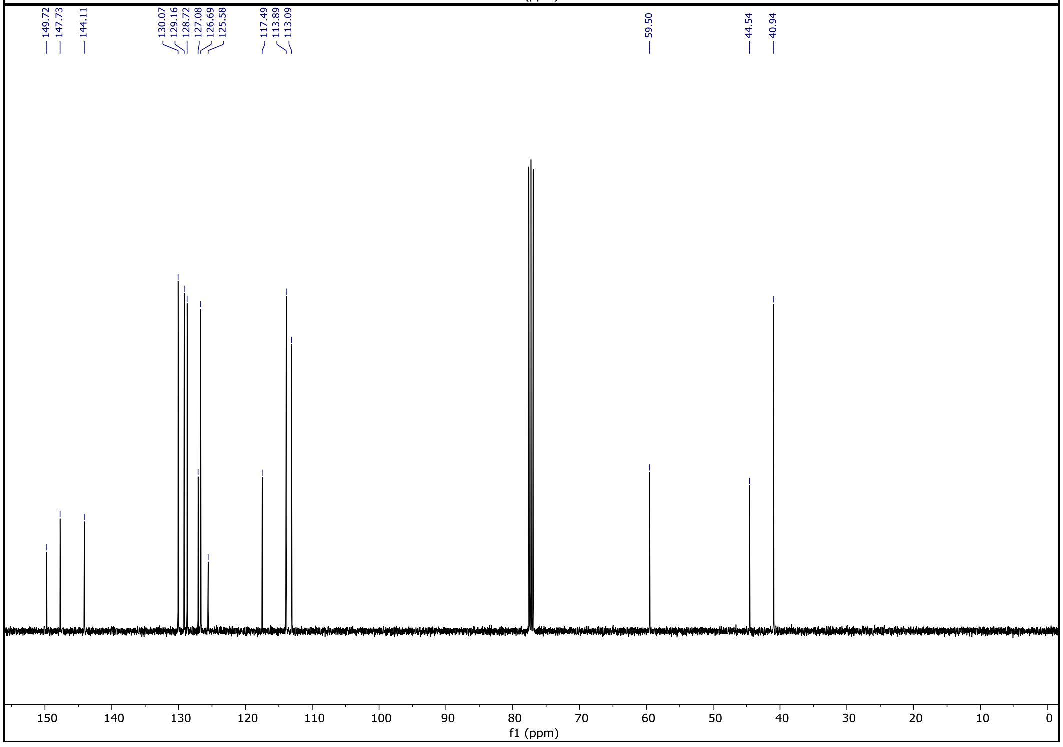
149.72
147.73
144.11
130.07
129.16
128.72
127.08
126.69
125.58
117.49
113.89
113.09
59.50
44.54
40.94
150 140 130 120 110 100 90 80 70 60 50 40 30 20 10 0
f1 (ppm)

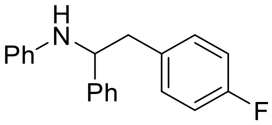

3ah

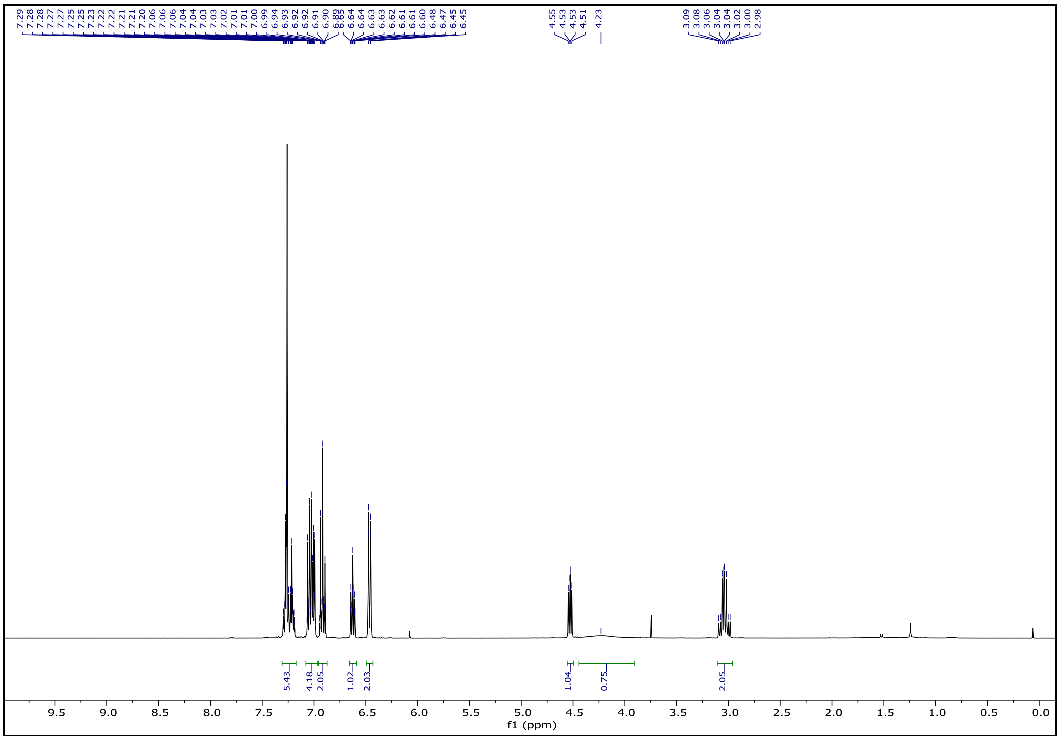

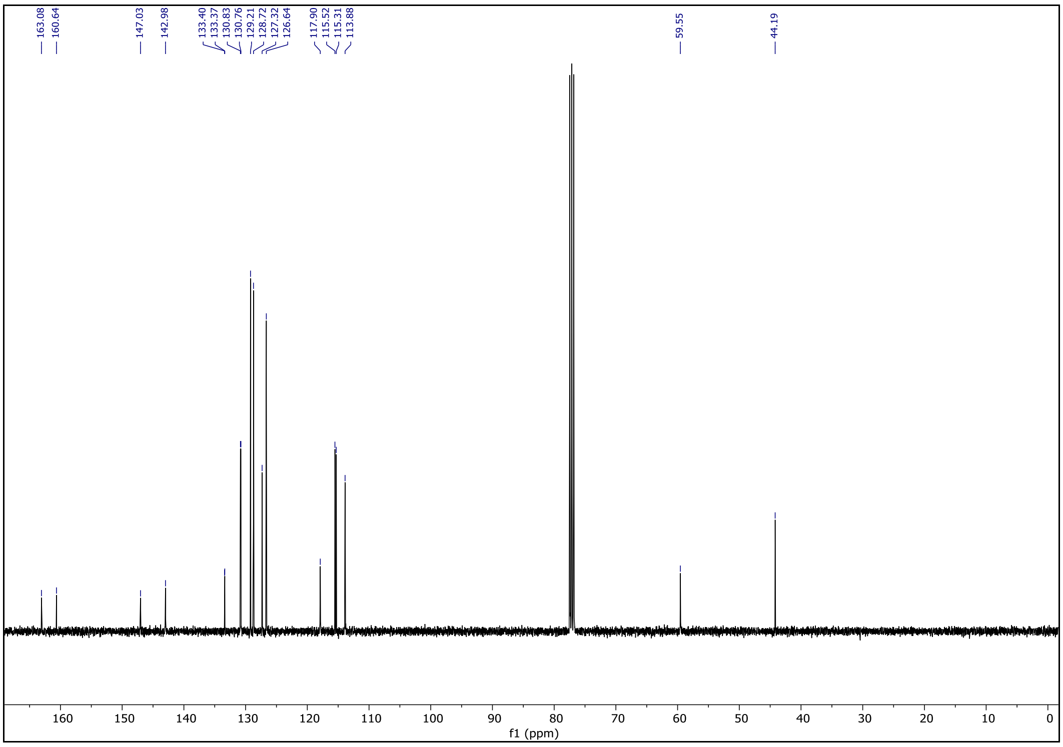

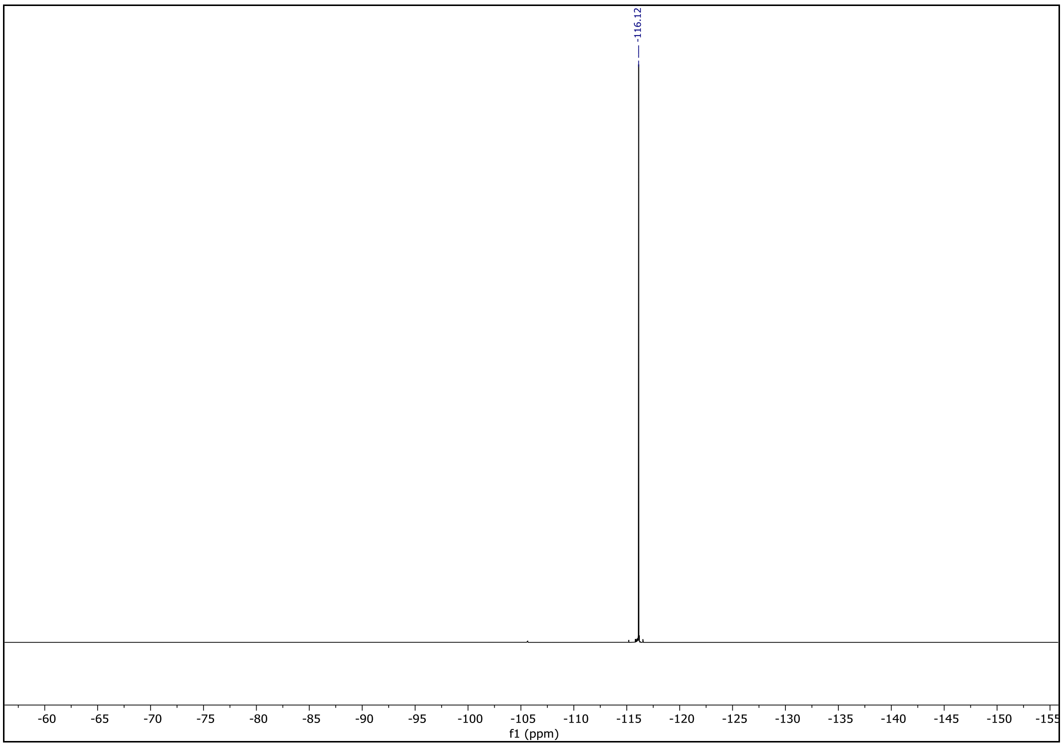
-116.12
-60
-65
-70
-75
-80
-85
-90
-95
-100
-105
-110
-115
-120
-125
-130
-135
-140
-145
-150
-155
f1 (ppm)

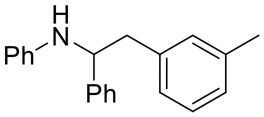

H
N
Ph
Ph

3ai

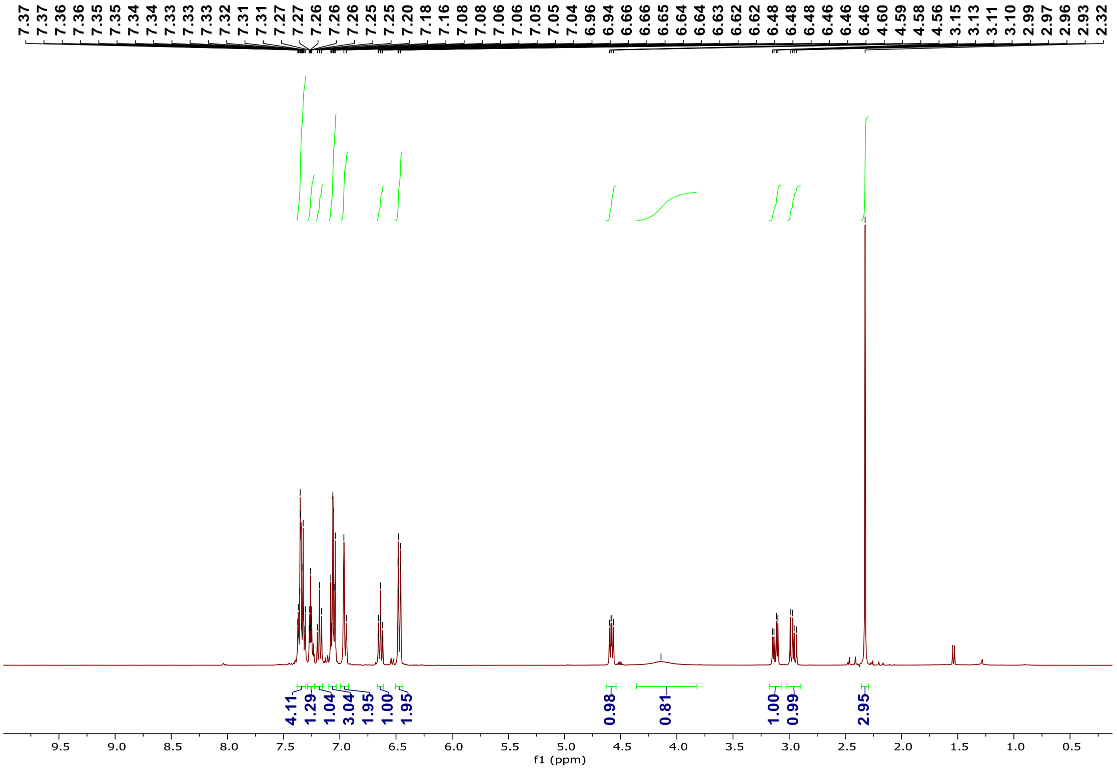

7.37 7.37 7.36 7.36 7.35 7.35 7.34 7.34 7.33 7.33 7.33 7.32 7.31 7.31 7.27 7.27 7.26 7.26 7.26 7.25 7.25 7.20 7.18 7.16 7.08 7.08 7.06 7.06 7.05 7.05 7.04 6.96 6.94 6.66 6.66 6.65 6.64 6.64 6.63 6.62 6.62 6.48 6.48 6.48 6.46 6.46 6.46 4.60 4.59 4.58 4.56 3.15 3.13 3.11 3.10 2.99 2.97 2.96 2.93 2.32
4.11 1.29 1.04 3.04 1.95 1.00 1.95 0.98 0.81 1.00 0.99 2.95
9.5 9.0 8.5 8.0 7.5 7.0 6.5 6.0 5.5 5.0 4.5 4.0 3.5 3.0 2.5 2.0 1.5 1.0 0.5
f1 (ppm)

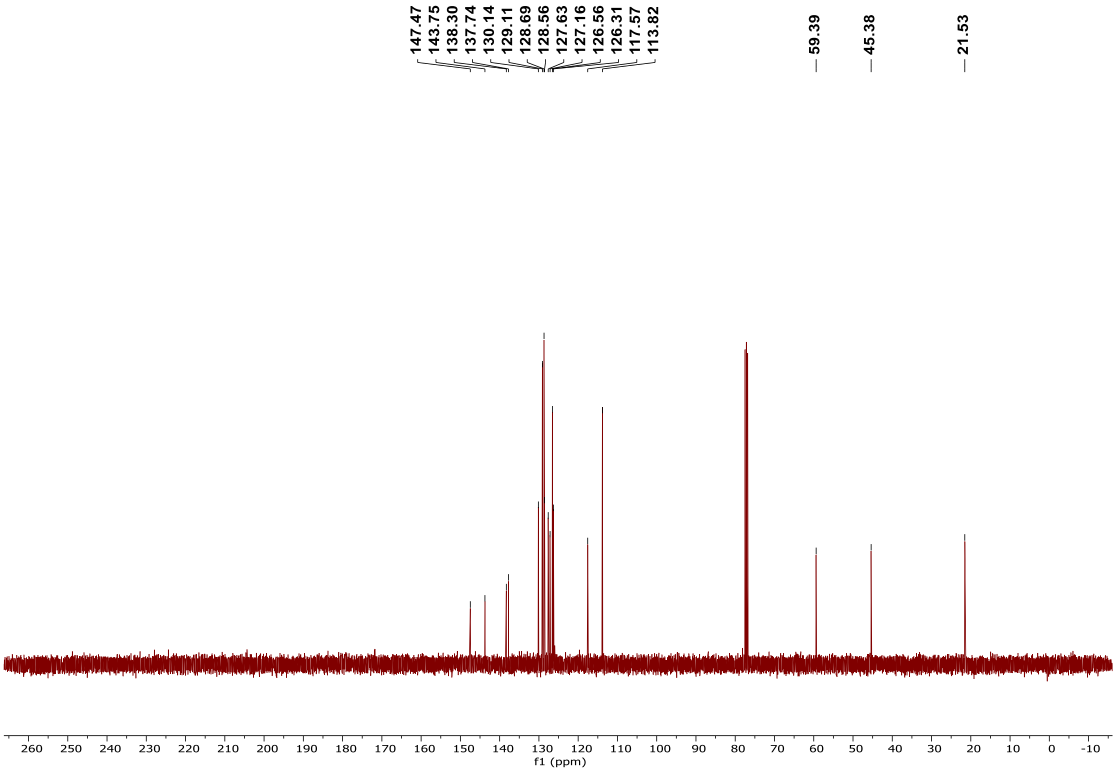

147.47 143.75 138.30 137.74 130.14 129.11 128.69 128.56 127.63 127.16 126.56 126.31 117.57 113.82
59.39
45.38
21.53
260 250 240 230 220 210 200 190 180 170 160 150 140 130 120 110 100 90 80 70 60 50 40 30 20 10 0 -10
f1 (ppm)

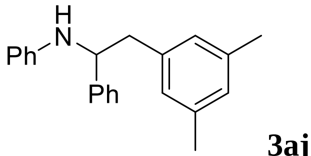

H
N
Ph
Ph

3aj

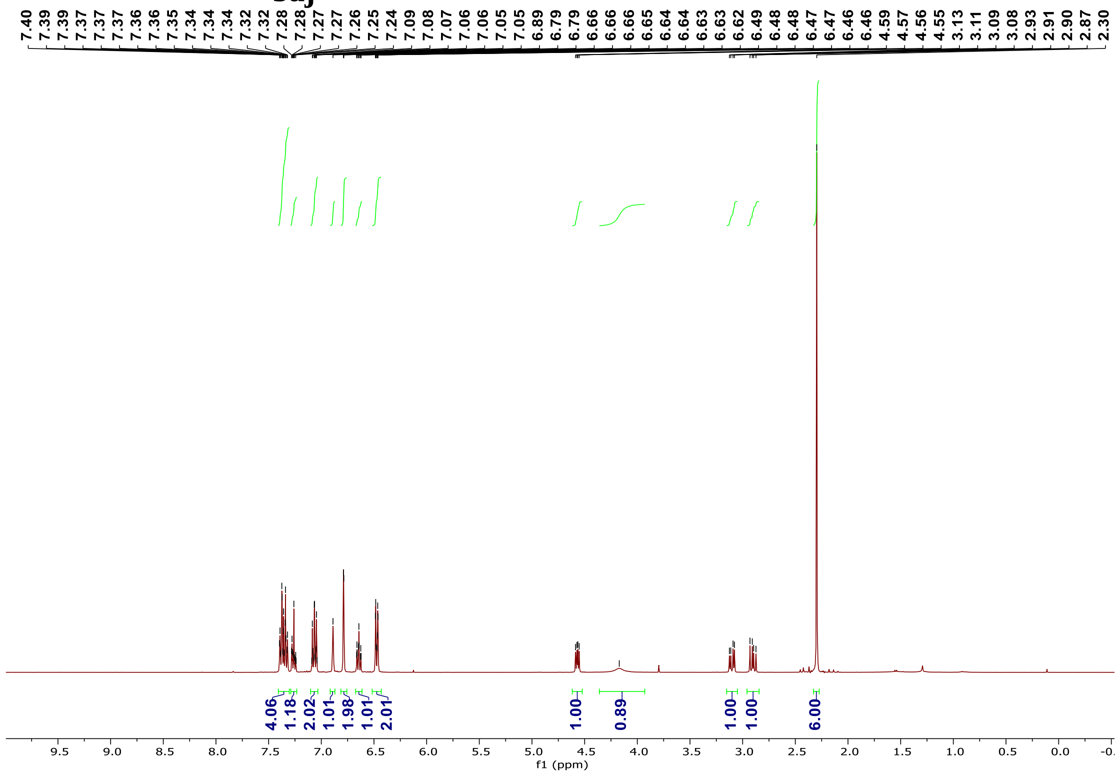

7.40 7.39 7.37 7.37 7.36 7.36 7.35 7.34 7.34 7.34 7.32 7.32 7.28 7.28 7.27 7.27 7.26 7.25 7.24 7.09 7.08 7.07 7.06 7.06 7.05 6.89 6.79 6.66 6.66 6.66 6.65 6.64 6.64 6.63 6.62 6.49 6.48 6.48 6.47 6.47 6.46 6.46 4.59 4.57 4.56 4.55 3.13 3.11 3.09 3.08 2.93 2.91 2.90 2.87 2.30
4.06 1.18 2.02 1.01 1.98 1.01 2.01 1.00 0.89 1.00 1.00 6.00
f1 (ppm)

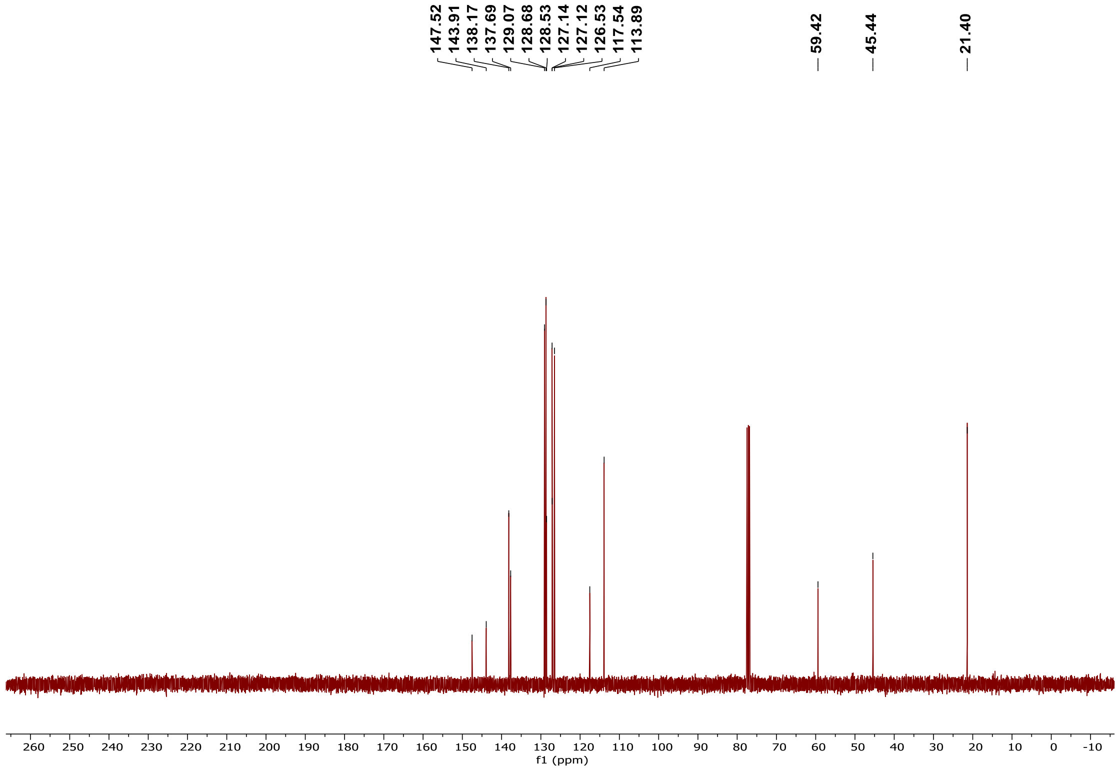

147.52 143.91 138.17 137.69 129.07 128.68 128.53 127.14 127.12 126.53 117.54 113.89
59.42
45.44
21.40
f1 (ppm)

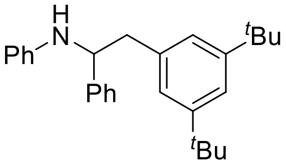

3ak

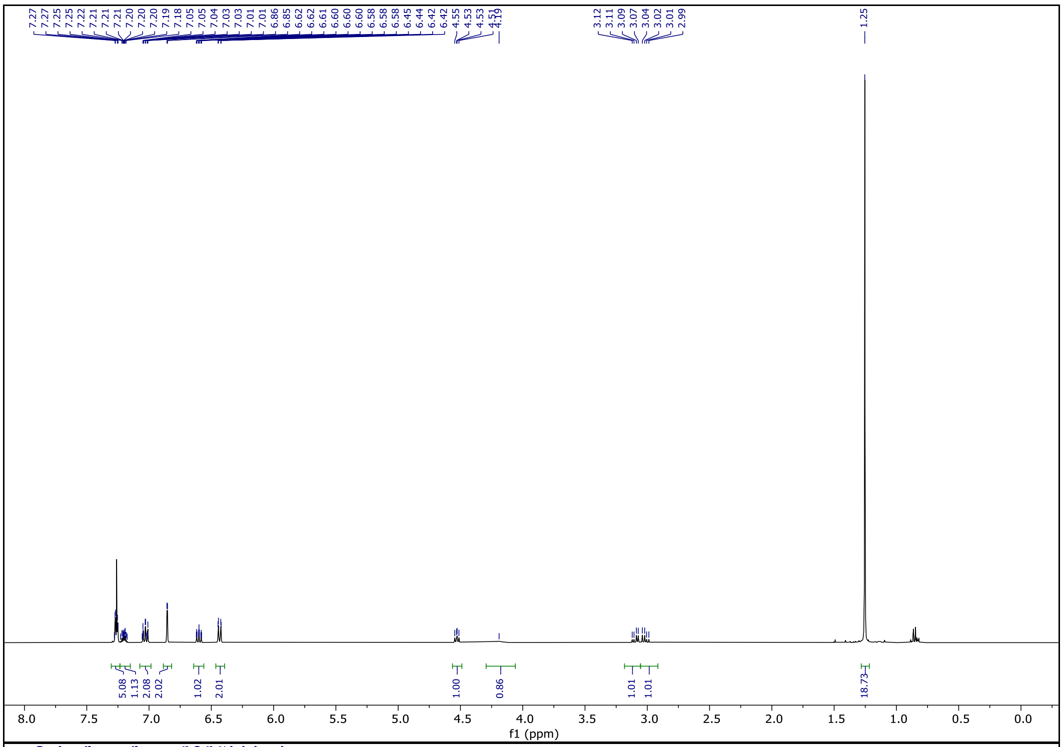

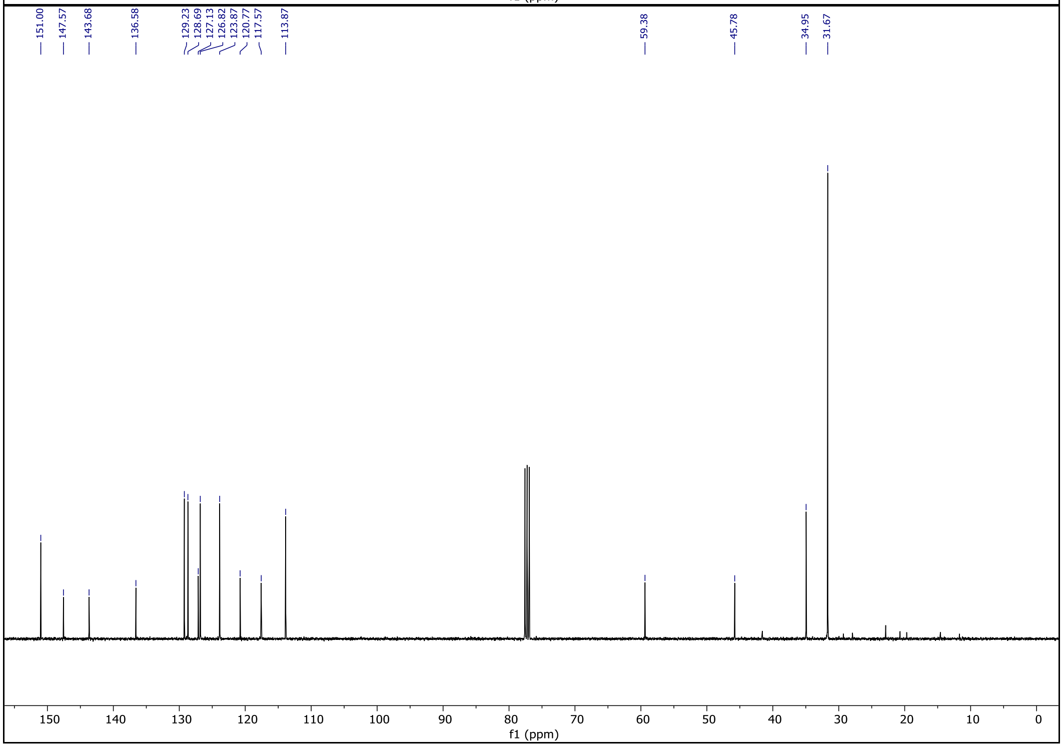

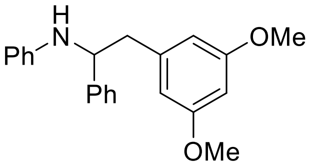

3al

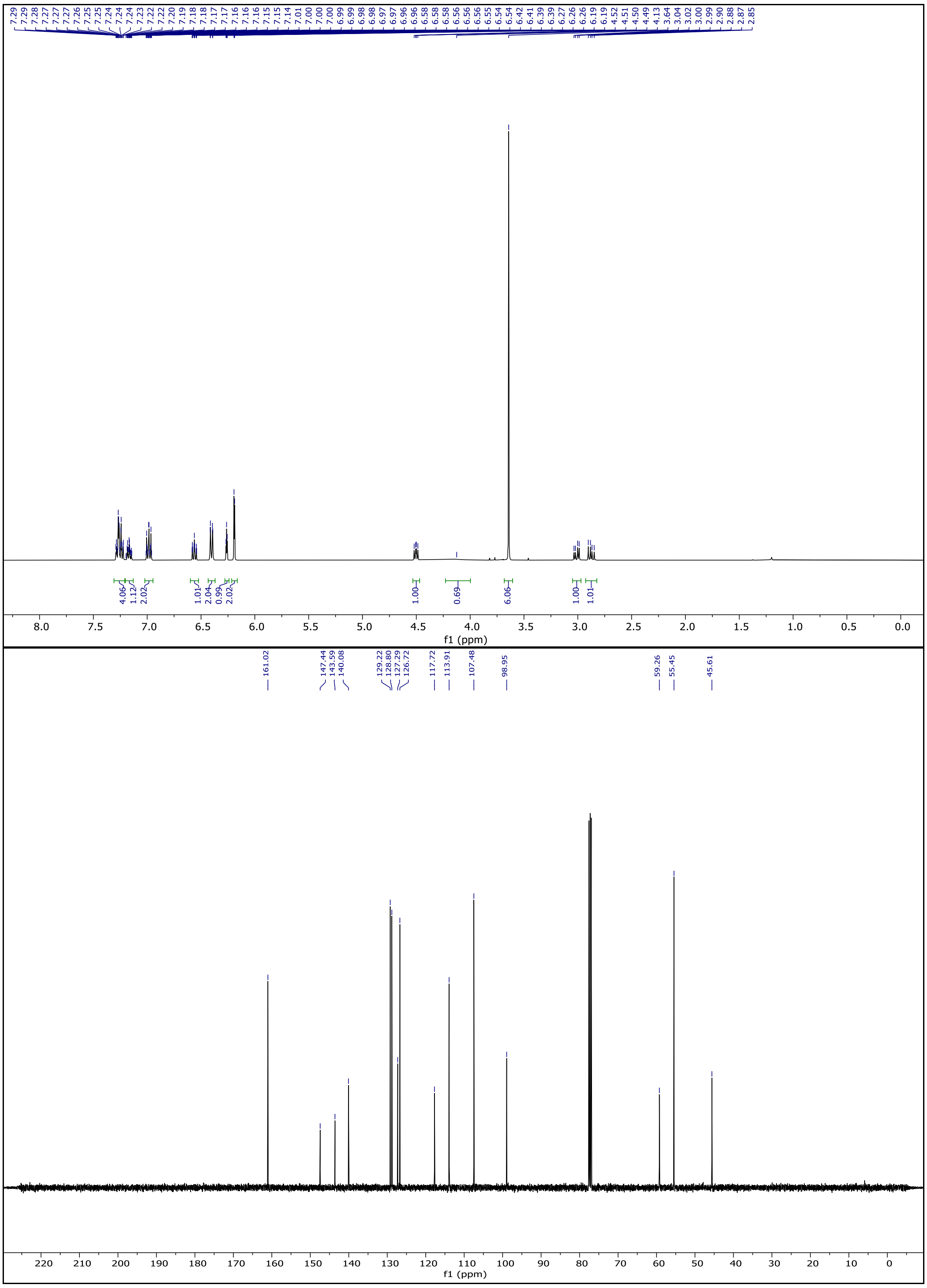

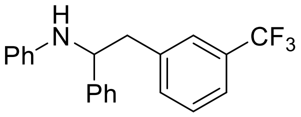

3am

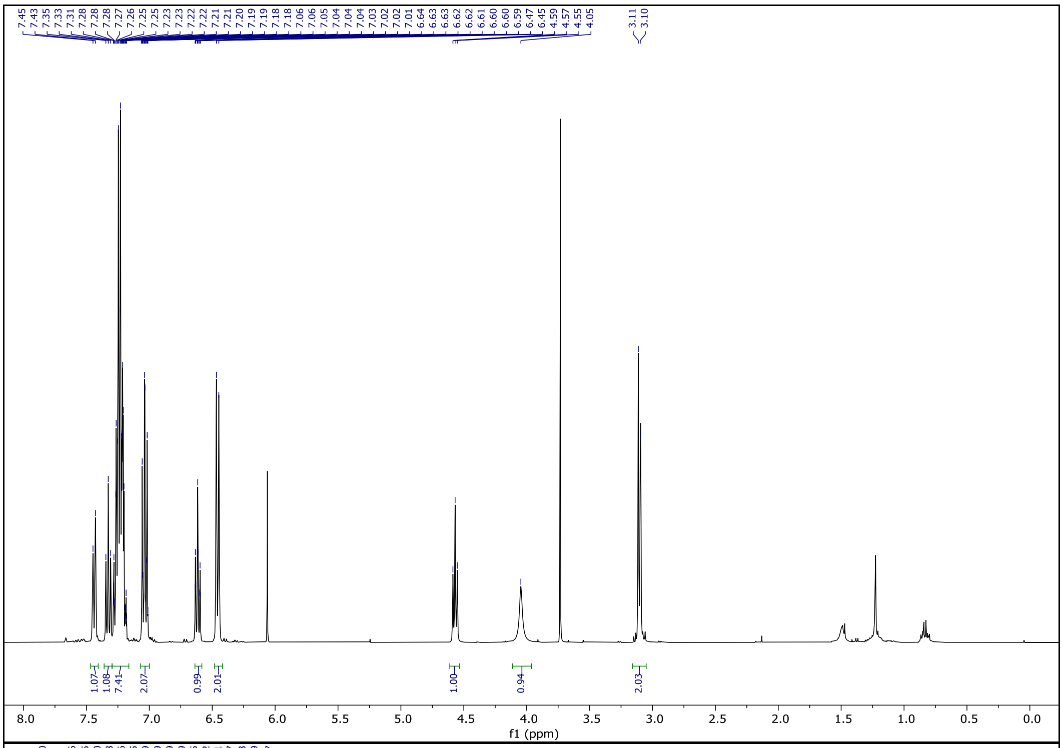

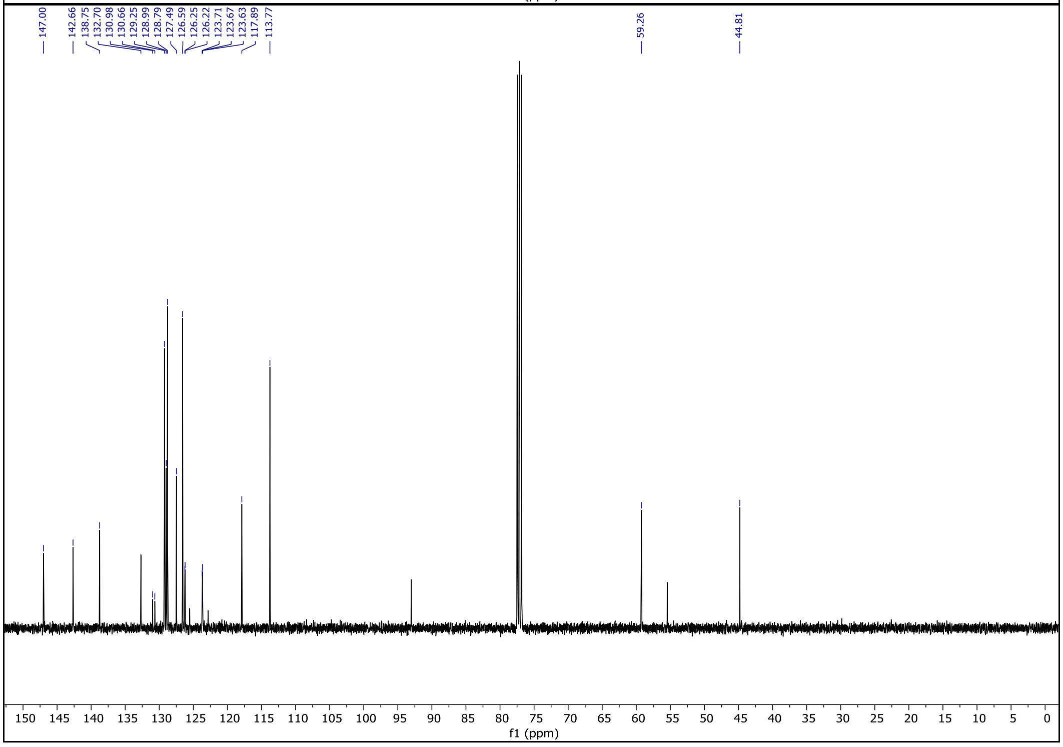

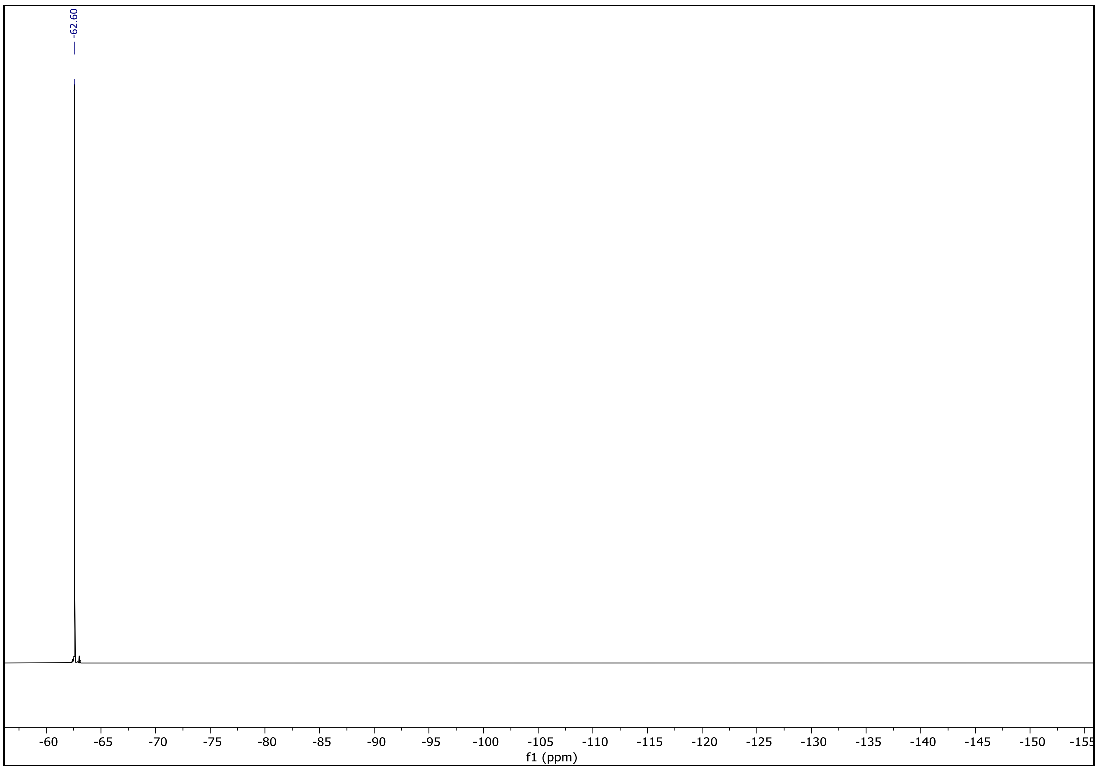
-62.60
-60
-65
-70
-75
-80
-85
-90
-95
-100
-105
-110
-115
-120
-125
-130
-135
-140
-145
-150
f1 (ppm)

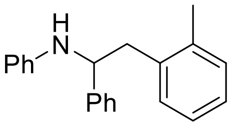

3an

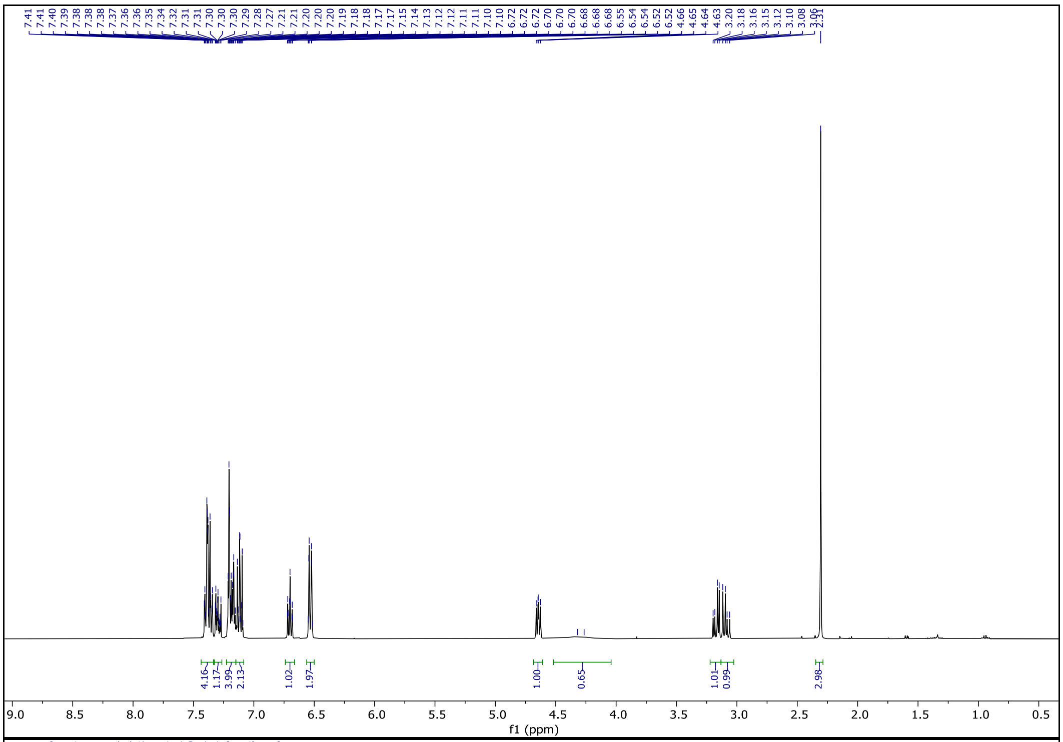

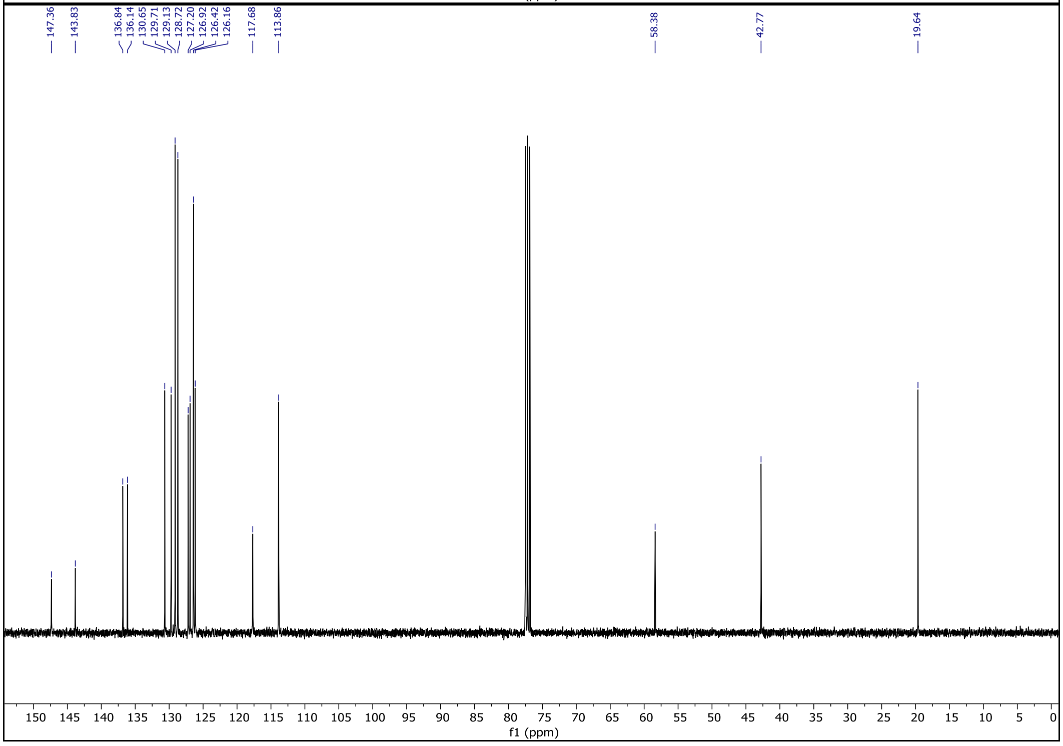

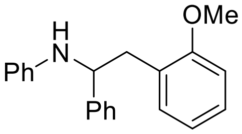

3ao

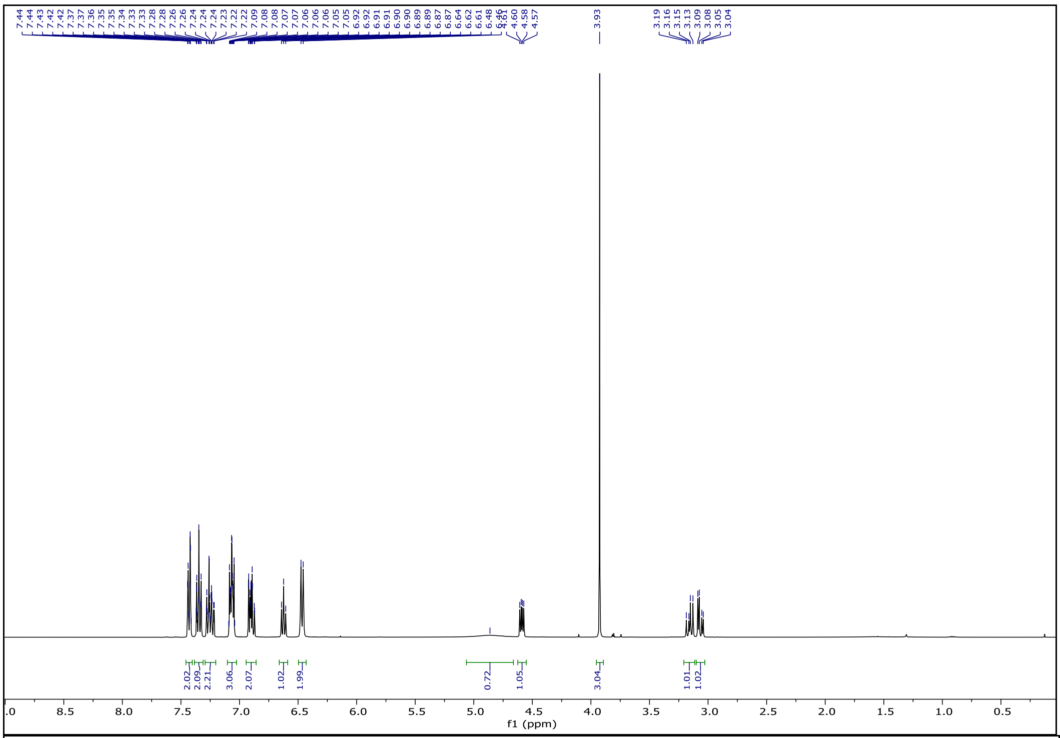

7.44
3.93
3.19
3.04
2.02
2.09
2.21
3.06
2.07
1.02
1.99
0.72
1.05
3.04
1.01
1.02
f1 (ppm)

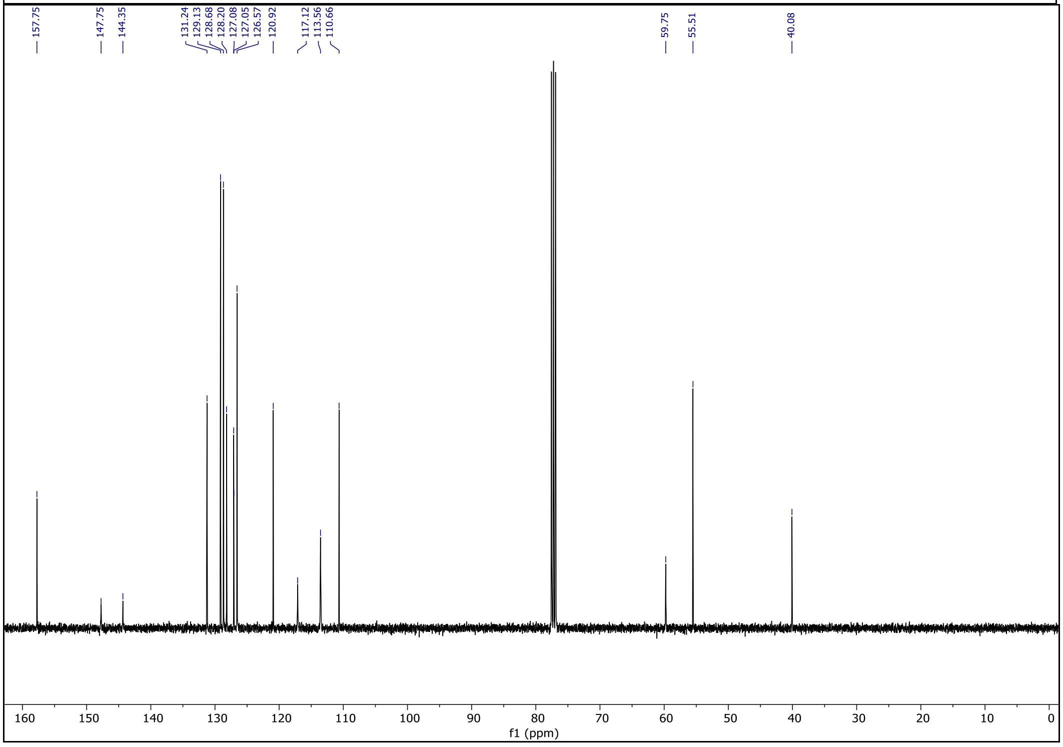

157.75
147.75
144.35
131.24
129.13
128.68
128.20
127.08
127.05
126.57
120.92
117.12
113.56
110.66
59.75
55.51
40.08
f1 (ppm)

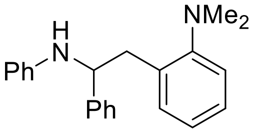

3ap

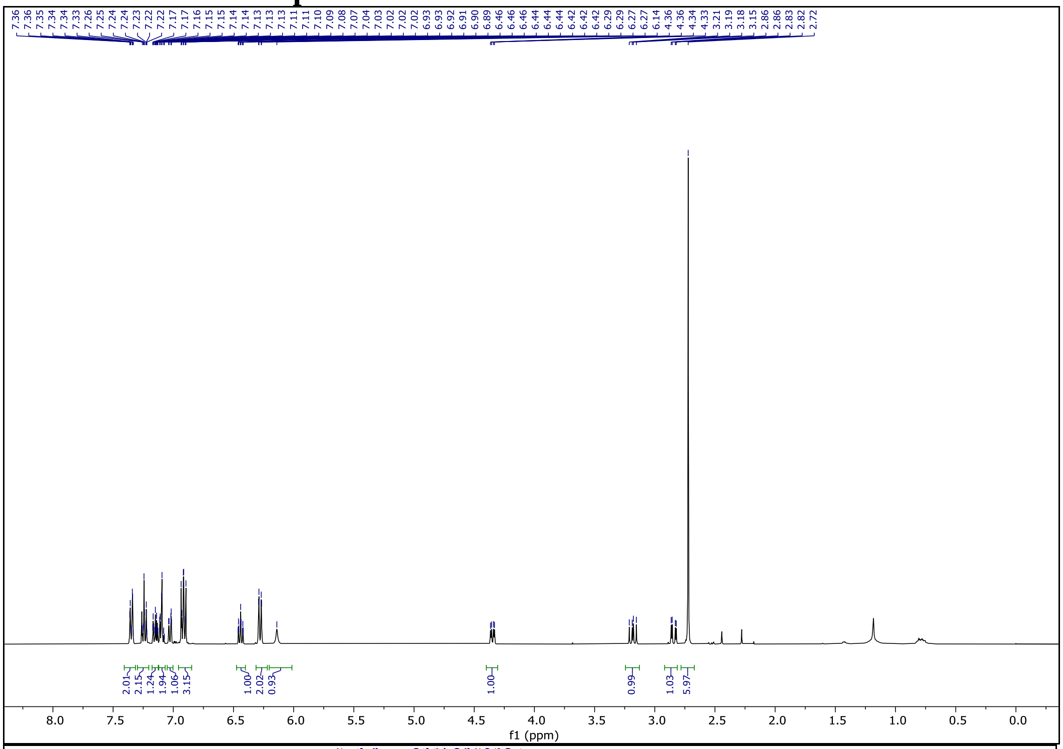

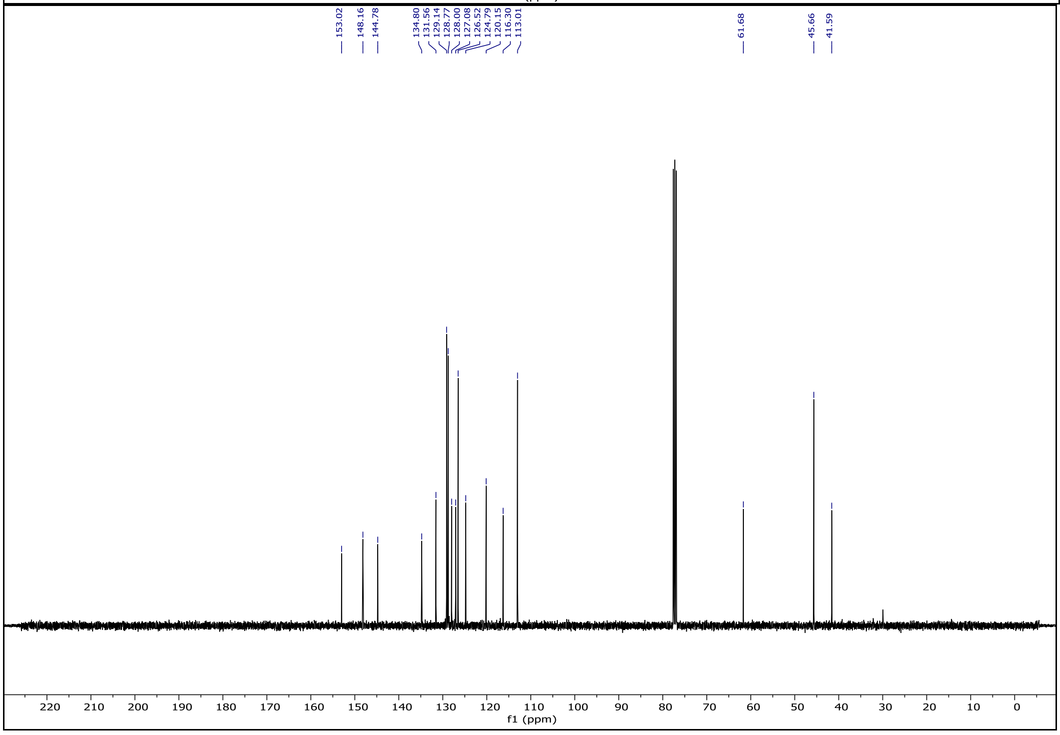

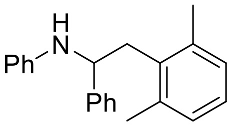

3aq

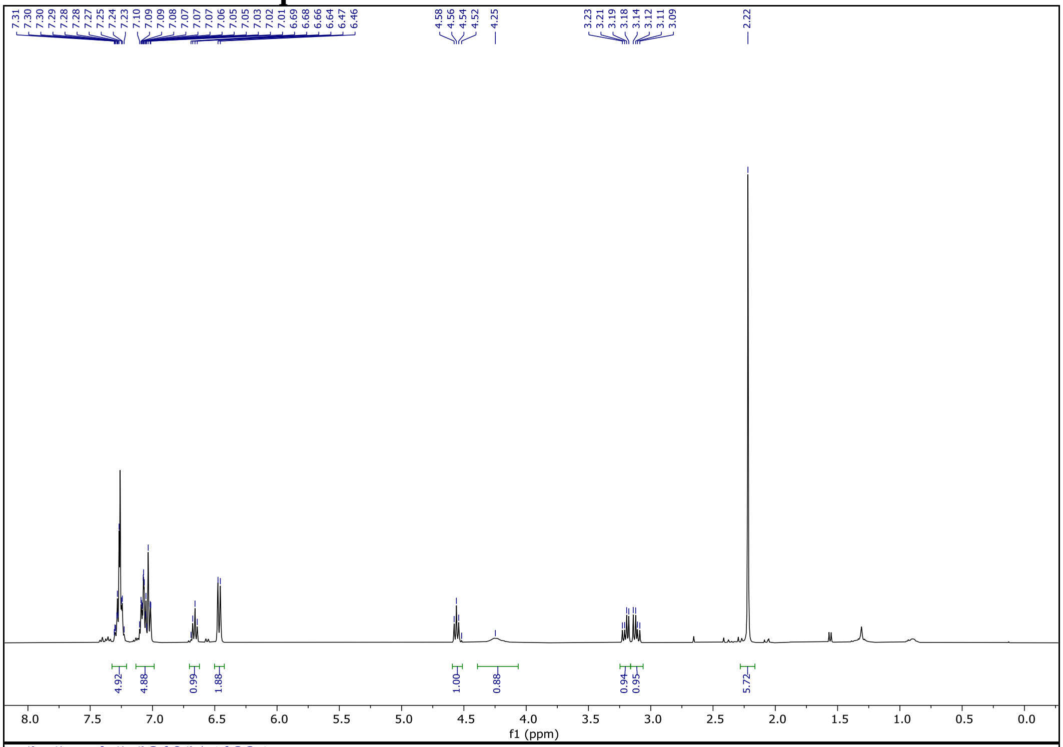

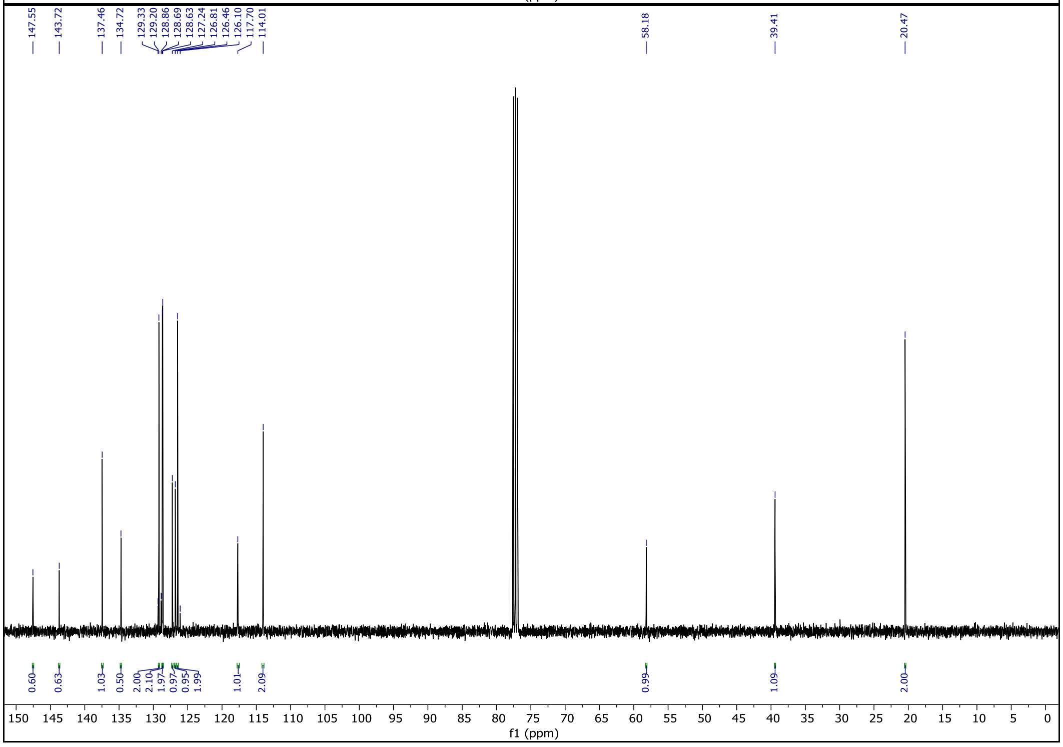

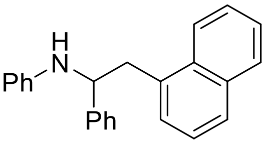

3ar

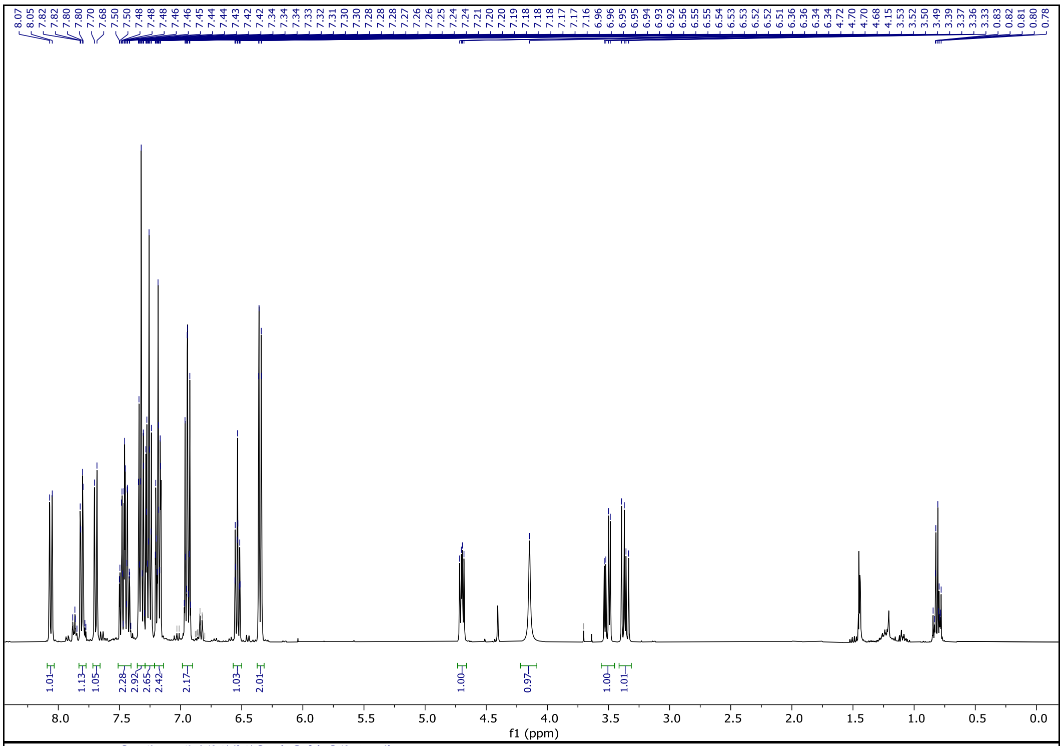

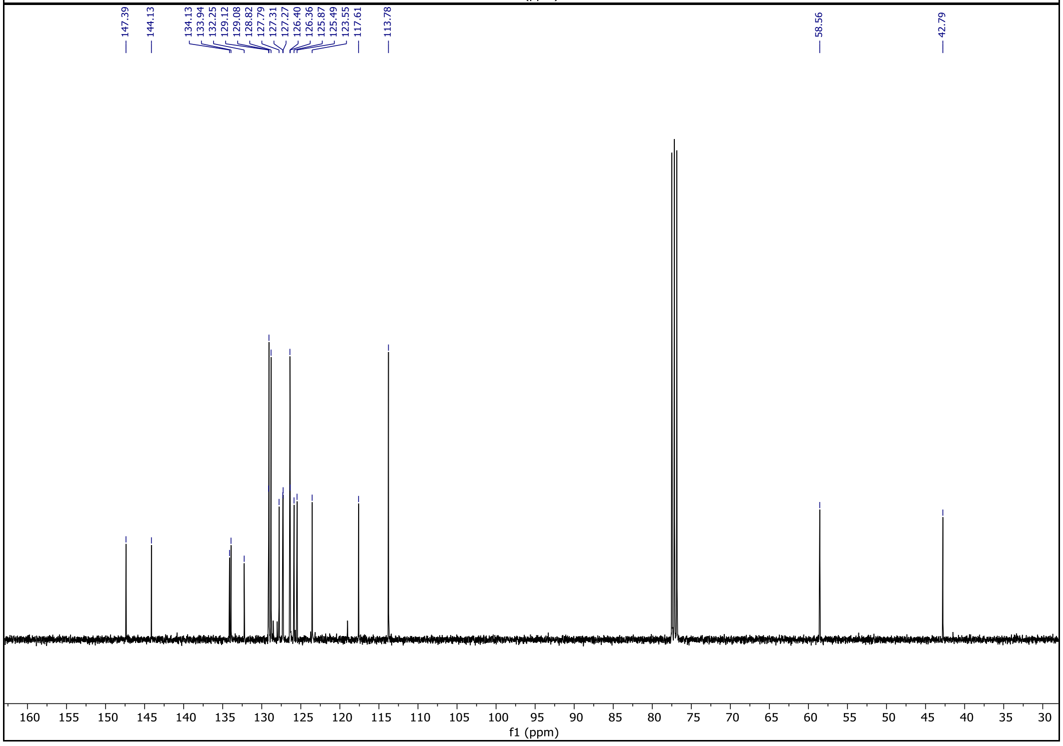

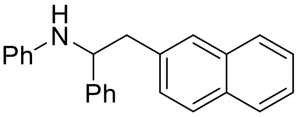

3as

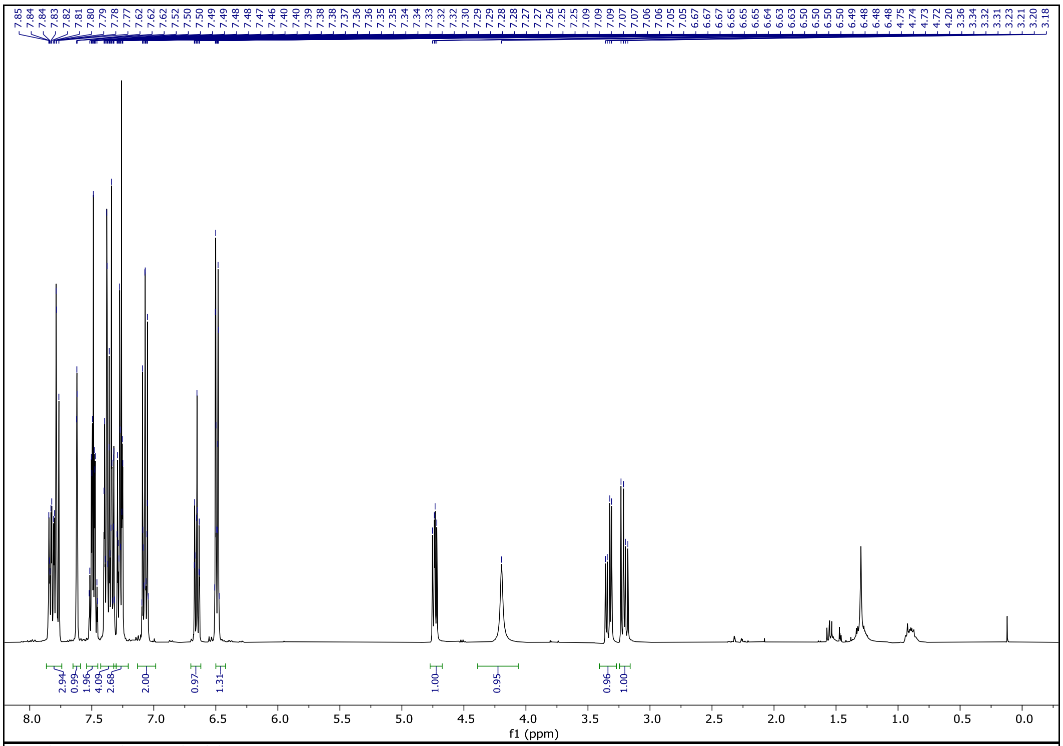

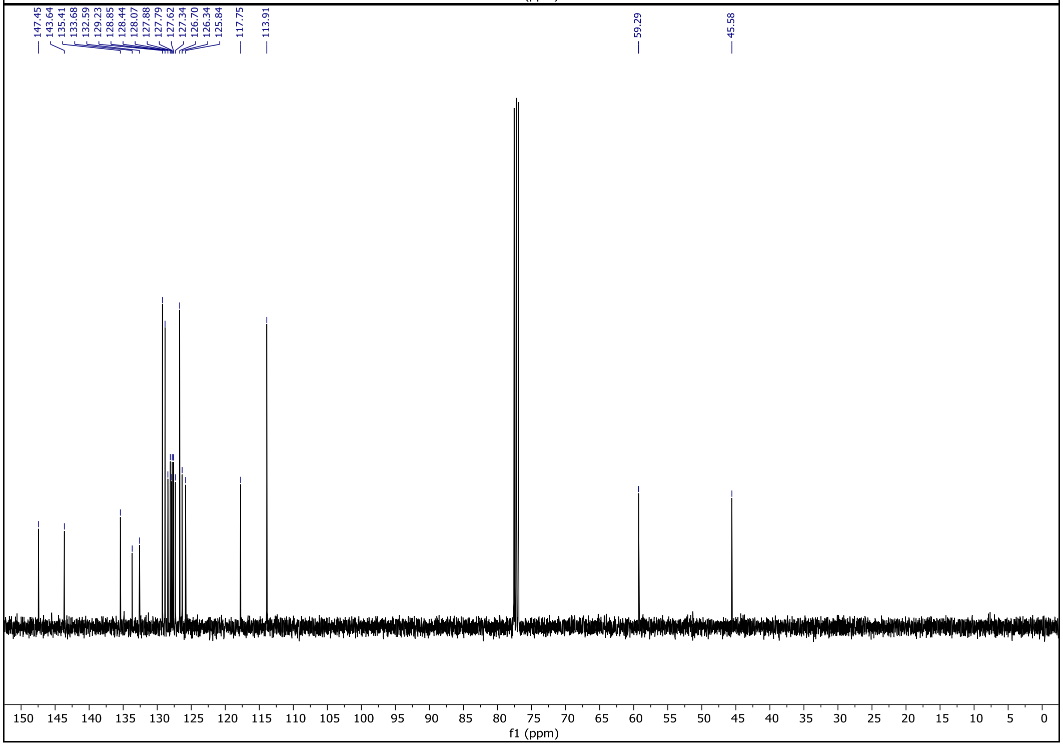

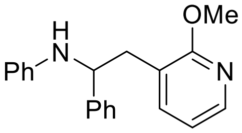
H
N
Ph
Ph
OMe
N

3at

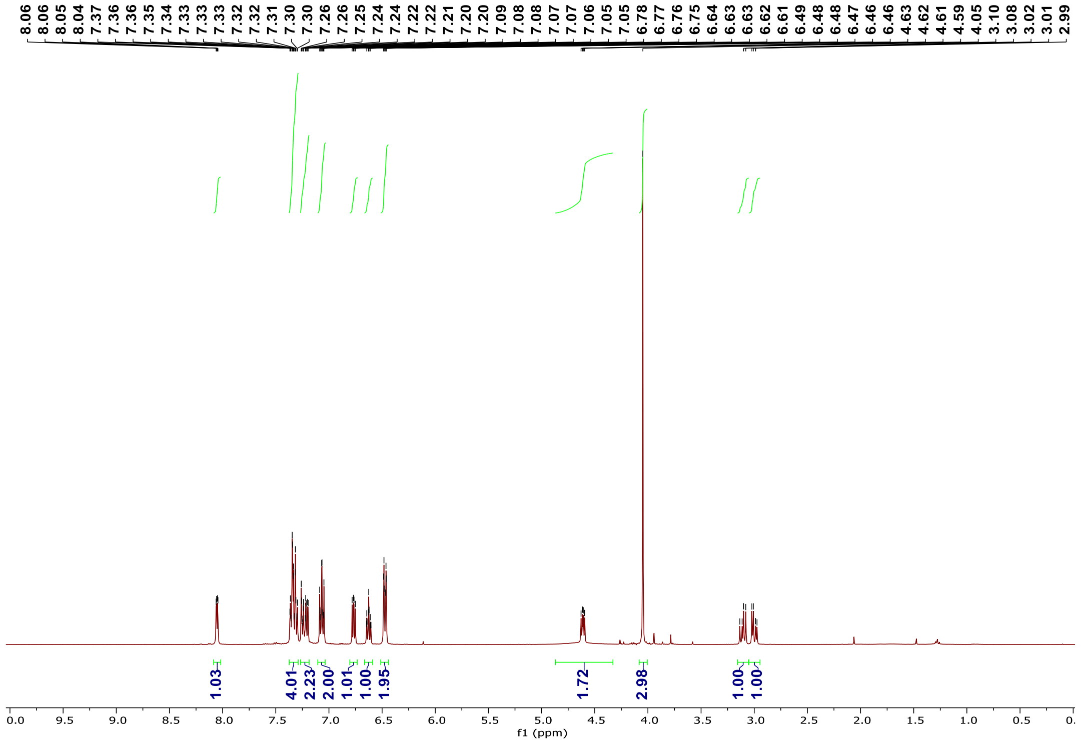
8.06
8.06
8.05
8.04
7.37
7.36
7.36
7.35
7.34
7.33
7.33
7.33
7.32
7.32
7.31
7.30
7.30
7.26
7.26
7.25
7.24
7.24
7.22
7.22
7.21
7.20
7.09
7.08
7.08
7.07
7.06
7.05
7.05
6.78
6.77
6.76
6.75
6.64
6.63
6.63
6.62
6.61
6.49
6.48
6.48
6.47
6.46
6.46
4.63
4.62
4.61
4.59
4.05
3.10
3.08
3.02
3.01
2.99
1.03
4.01
2.23
2.00
1.01
1.00
1.95
1.72
2.98
1.00
1.00
f1 (ppm)

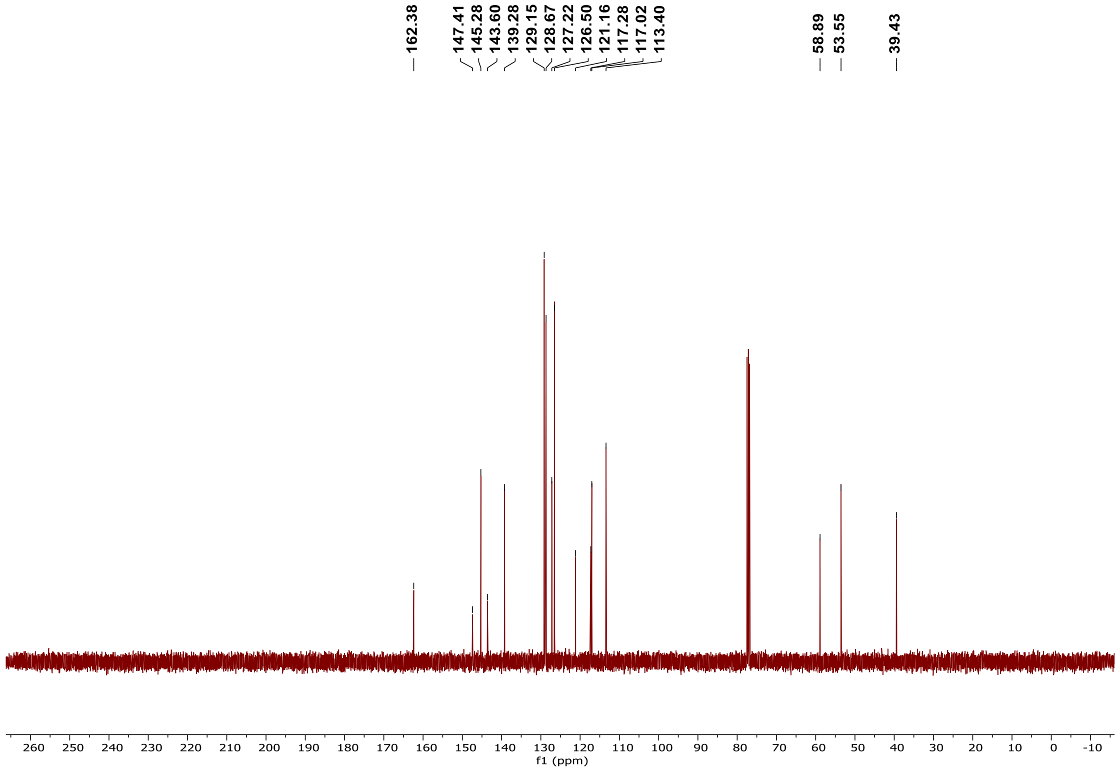
162.38
147.41
145.28
143.60
139.28
129.15
128.67
127.22
126.50
121.16
117.28
117.02
113.40
58.89
53.55
39.43
f1 (ppm)

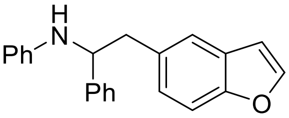
H
N
Ph
Ph
O

3au

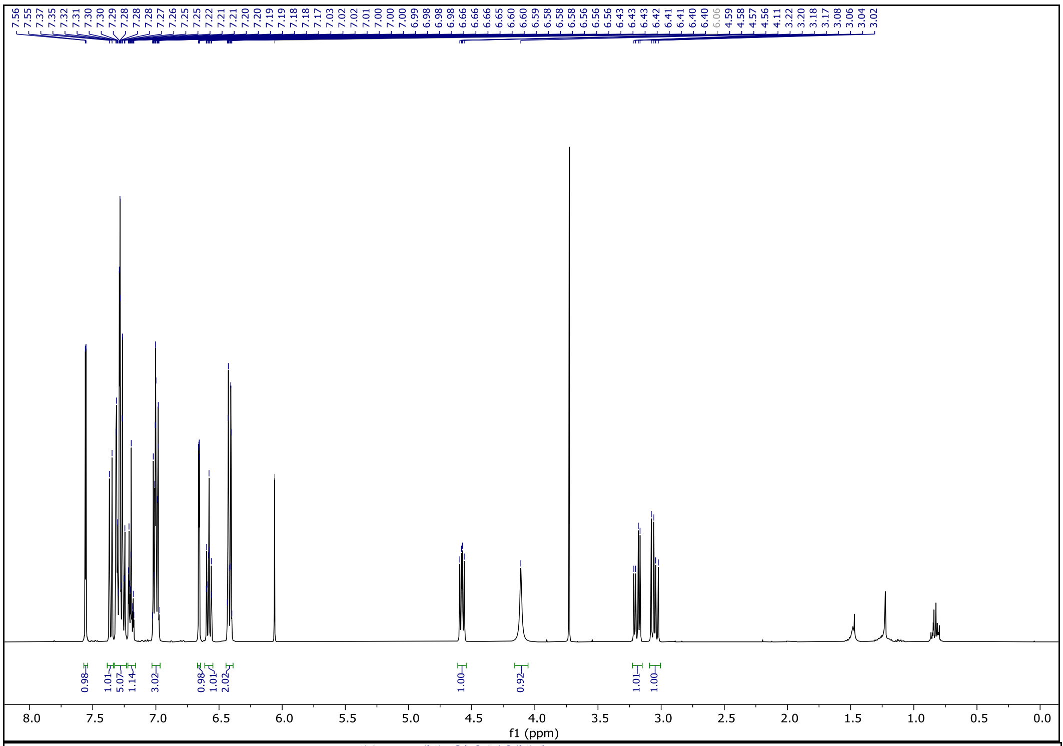
7.56 7.55 7.37 7.35 7.32 7.31 7.30 7.30 7.29 7.28 7.28 7.28 7.27 7.26 7.25 7.25 7.22 7.22 7.21 7.21 7.20 7.20 7.19 7.19 7.18 7.18 7.17 7.03 7.02 7.02 7.01 7.00 7.00 7.00 6.99 6.99 6.98 6.98 6.98 6.66 6.66 6.66 6.65 6.60 6.60 6.59 6.58 6.58 6.58 6.56 6.56 6.43 6.43 6.42 6.41 6.41 6.40 6.40 6.06 4.59 4.58 4.57 4.56 4.11 3.22 3.20 3.18 3.17 3.08 3.06 3.04 3.02
0.98 1.01 5.07 1.14 3.02 0.98 1.01 2.02 1.00 0.92 1.01 1.00
8.0 7.5 7.0 6.5 6.0 5.5 5.0 4.5 4.0 3.5 3.0 2.5 2.0 1.5 1.0 0.5 0.0
f1 (ppm)

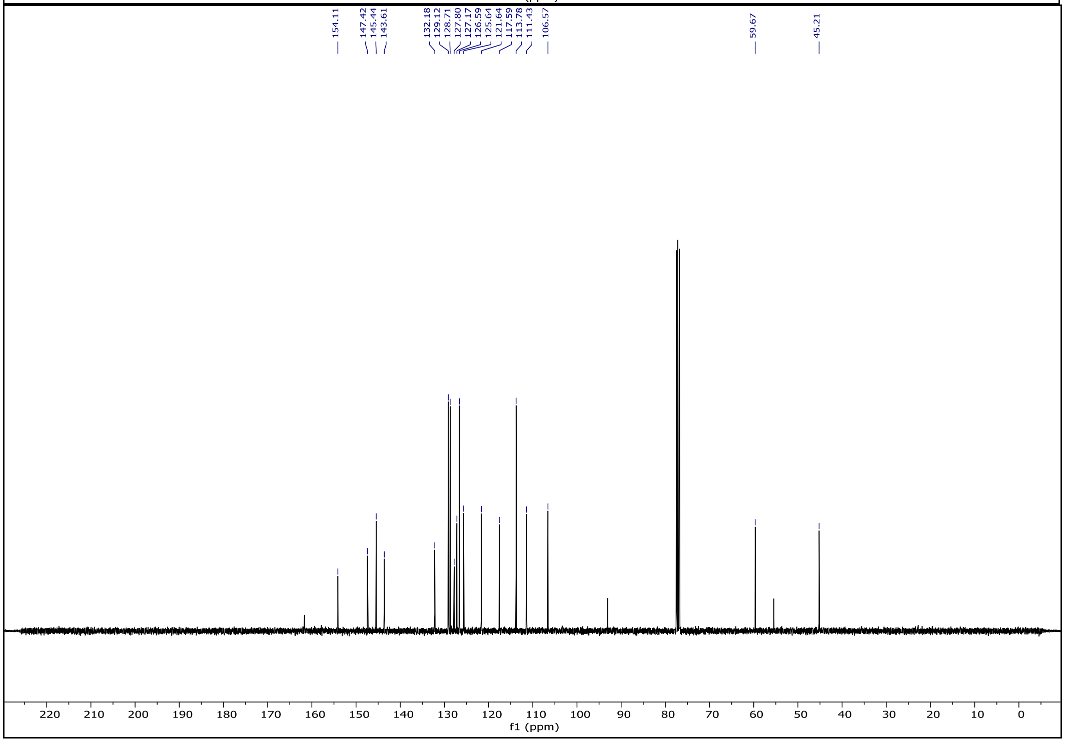
154.11 147.42 145.44 143.61 132.18 129.12 128.71 127.80 127.17 126.59 125.64 121.64 117.59 113.78 111.43 106.57 59.67 45.21
220 210 200 190 180 170 160 150 140 130 120 110 100 90 80 70 60 50 40 30 20 10 0
f1 (ppm)

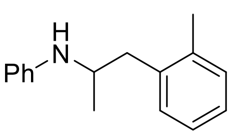
H
N
Ph

3bn

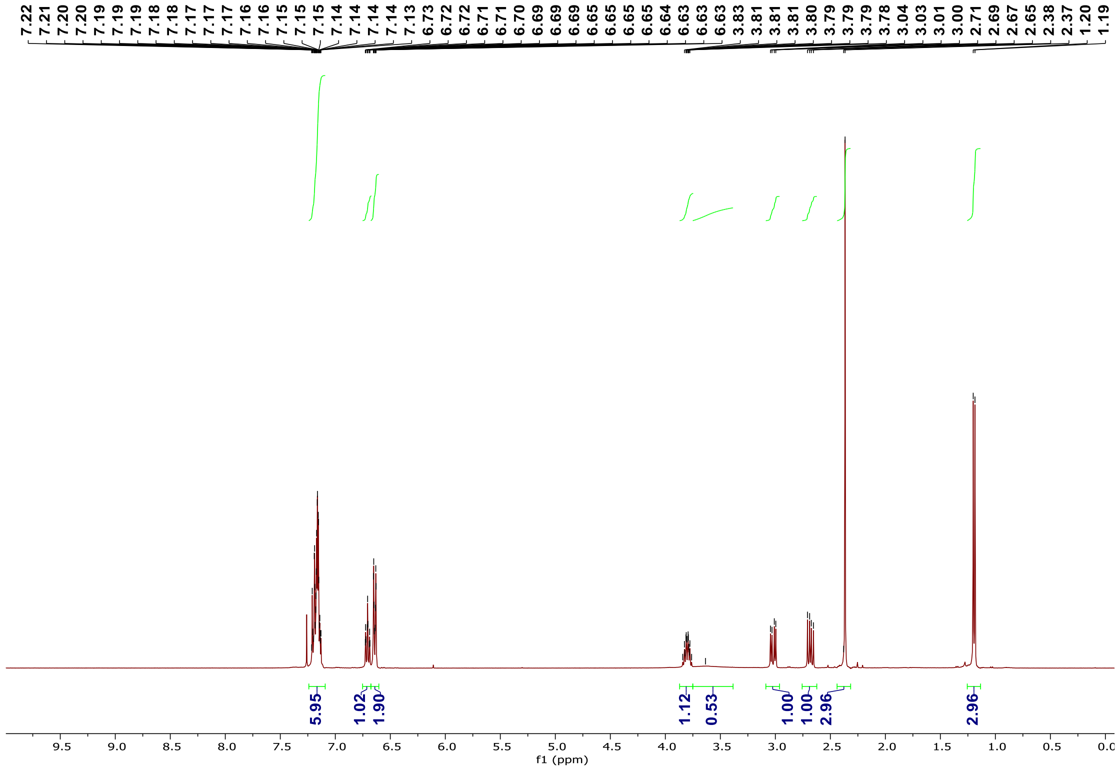
7.22
7.21
7.20
7.20
7.19
7.19
7.19
7.18
7.17
7.17
7.17
7.16
7.16
7.15
7.15
7.15
7.14
7.14
7.14
7.13
6.73
6.72
6.72
6.71
6.71
6.70
6.69
6.69
6.69
6.65
6.65
6.65
6.64
6.63
6.63
6.63
3.83
3.81
3.81
3.81
3.80
3.79
3.79
3.79
3.78
3.04
3.03
3.01
3.00
2.71
2.69
2.67
2.65
2.38
2.37
1.20
1.19
5.95
1.02
1.90
1.12
0.53
1.00
1.00
2.96
2.96
f1 (ppm)

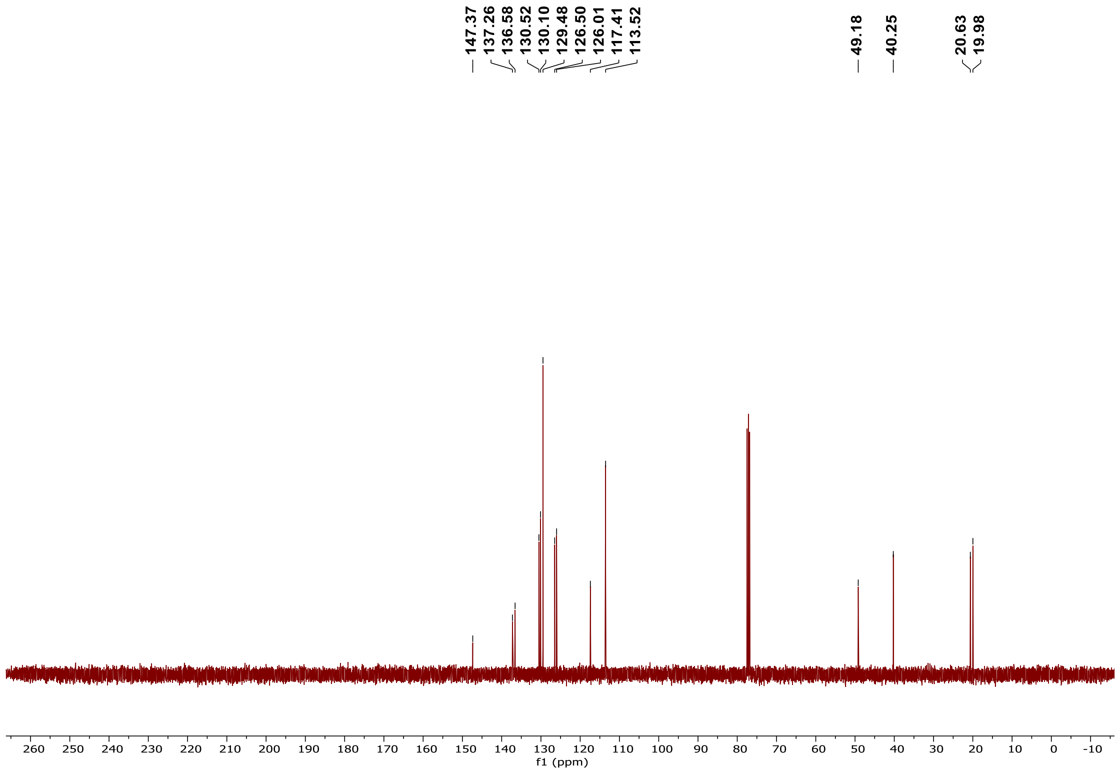
147.37
137.26
136.58
130.52
130.10
129.48
126.50
126.01
117.41
113.52
49.18
40.25
20.63
19.98
f1 (ppm)

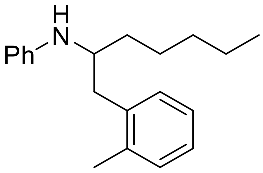
Ph
N
H

3cn

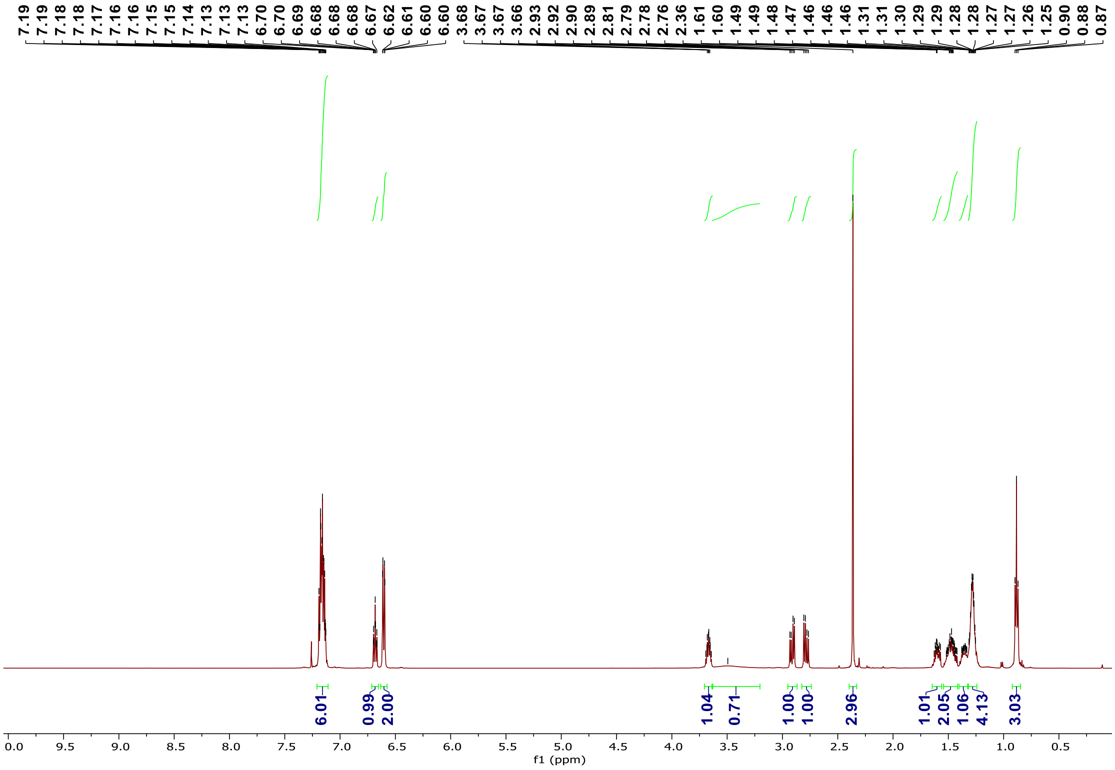
7.19 7.19 7.18 7.17 7.16 7.16 7.15 7.14 7.13 7.13 7.13 6.70 6.70 6.69 6.68 6.68 6.68 6.67 6.62 6.61 6.60 6.60 3.68 3.67 3.66 2.93 2.92 2.90 2.89 2.81 2.79 2.78 2.76 2.36 1.61 1.60 1.49 1.49 1.48 1.47 1.46 1.46 1.46 1.31 1.31 1.30 1.29 1.29 1.28 1.28 1.27 1.27 1.26 1.25 0.90 0.88 0.87
6.01 0.99 2.00 1.04 0.71 1.00 1.00 2.96 1.01 2.05 1.06 4.13 3.03
f1 (ppm)

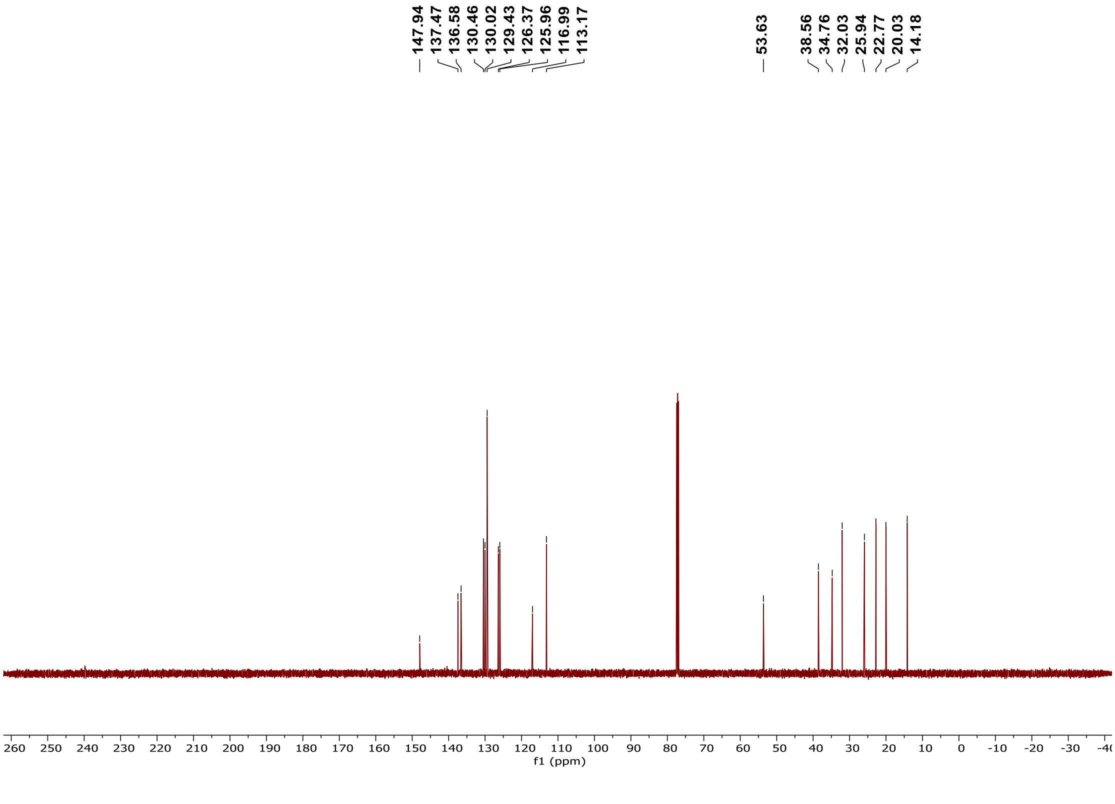
147.94 137.47 136.58 130.46 130.02 129.43 126.37 125.96 116.99 113.17 53.63 38.56 34.76 32.03 25.94 22.77 20.03 14.18
f1 (ppm)

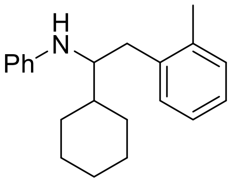
H
N
Ph

3dn

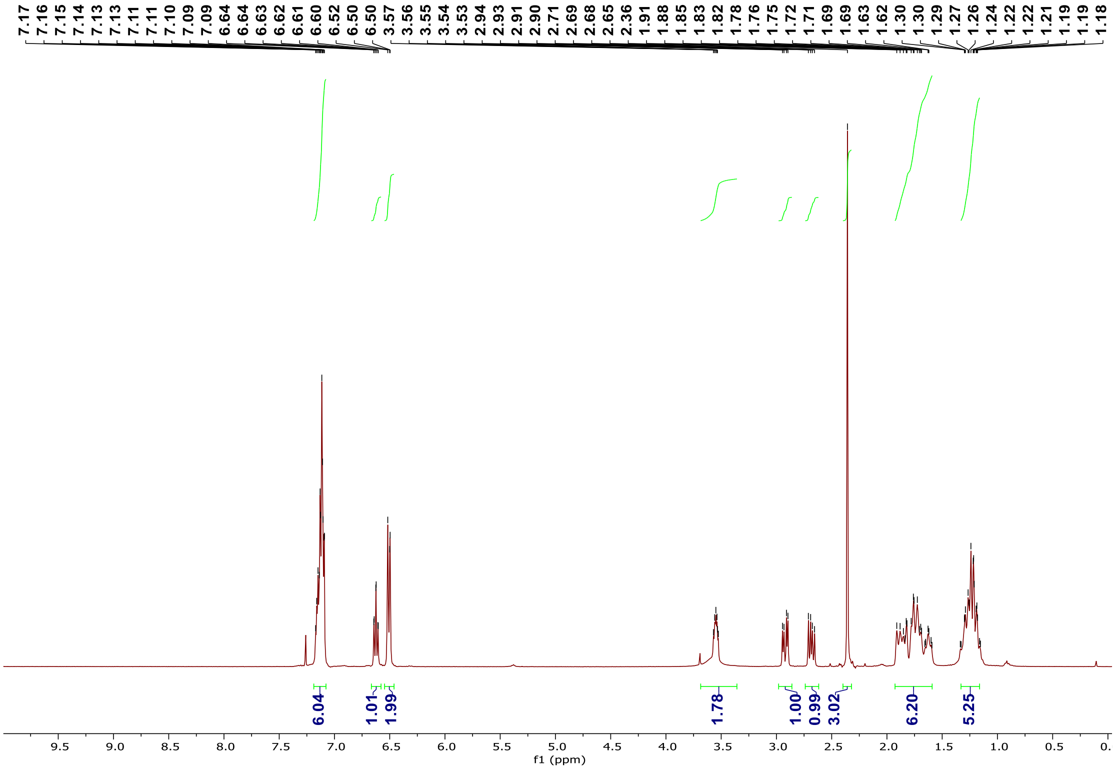
7.17
7.16
7.15
7.14
7.13
7.13
7.11
7.11
7.10
7.09
7.09
6.64
6.63
6.62
6.61
6.60
6.52
6.50
6.50
3.57
3.56
3.55
3.54
3.53
2.94
2.93
2.91
2.90
2.71
2.69
2.68
2.65
2.36
1.91
1.88
1.85
1.83
1.82
1.78
1.76
1.75
1.72
1.71
1.69
1.69
1.63
1.62
1.30
1.30
1.29
1.27
1.26
1.24
1.22
1.22
1.21
1.19
1.19
1.18
6.04
1.01
1.99
1.78
1.00
0.99
3.02
6.20
5.25
9.5
9.0
8.5
8.0
7.5
7.0
6.5
6.0
5.5
5.0
4.5
4.0
3.5
3.0
2.5
2.0
1.5
1.0
0.5
0.
f1 (ppm)

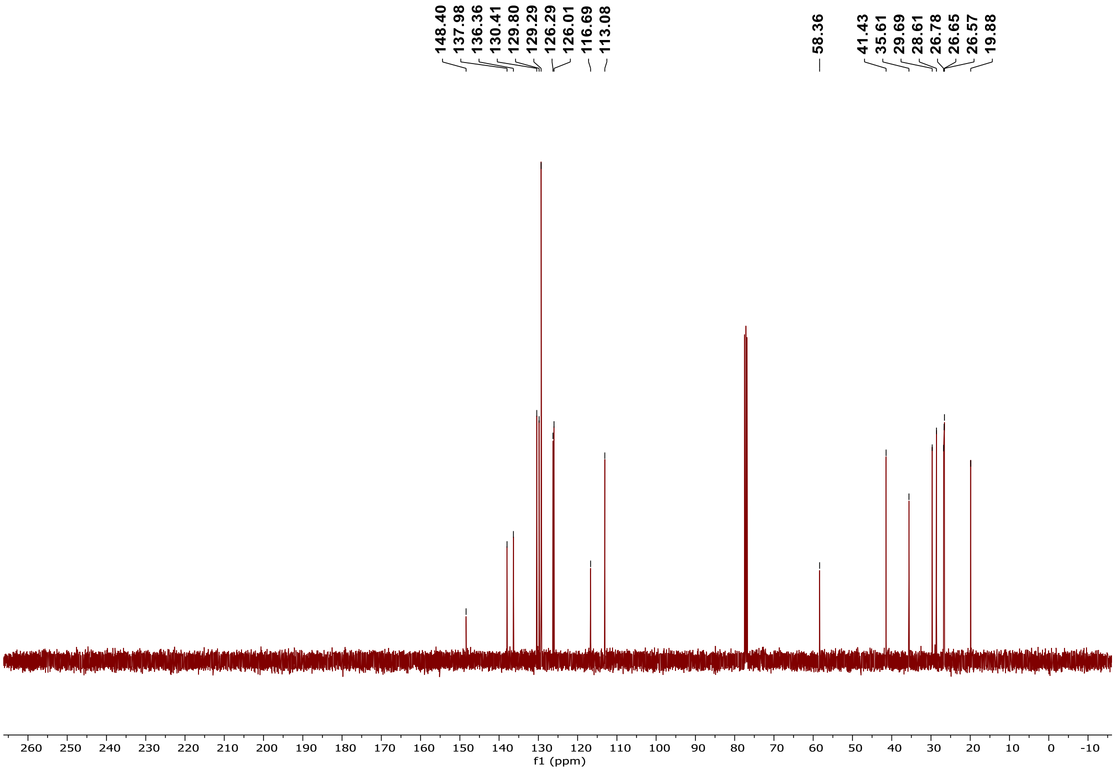
148.40
137.98
136.36
130.41
129.80
129.29
126.29
126.01
116.69
113.08
58.36
41.43
35.61
29.69
28.61
26.78
26.65
26.57
19.88
260
250
240
230
220
210
200
190
180
170
160
150
140
130
120
110
100
90
80
70
60
50
40
30
20
10
0
-10
f1 (ppm)

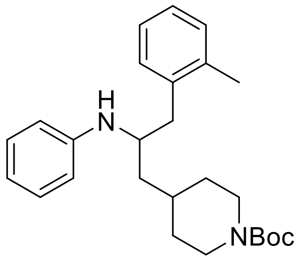
H
N
NBoc

3en

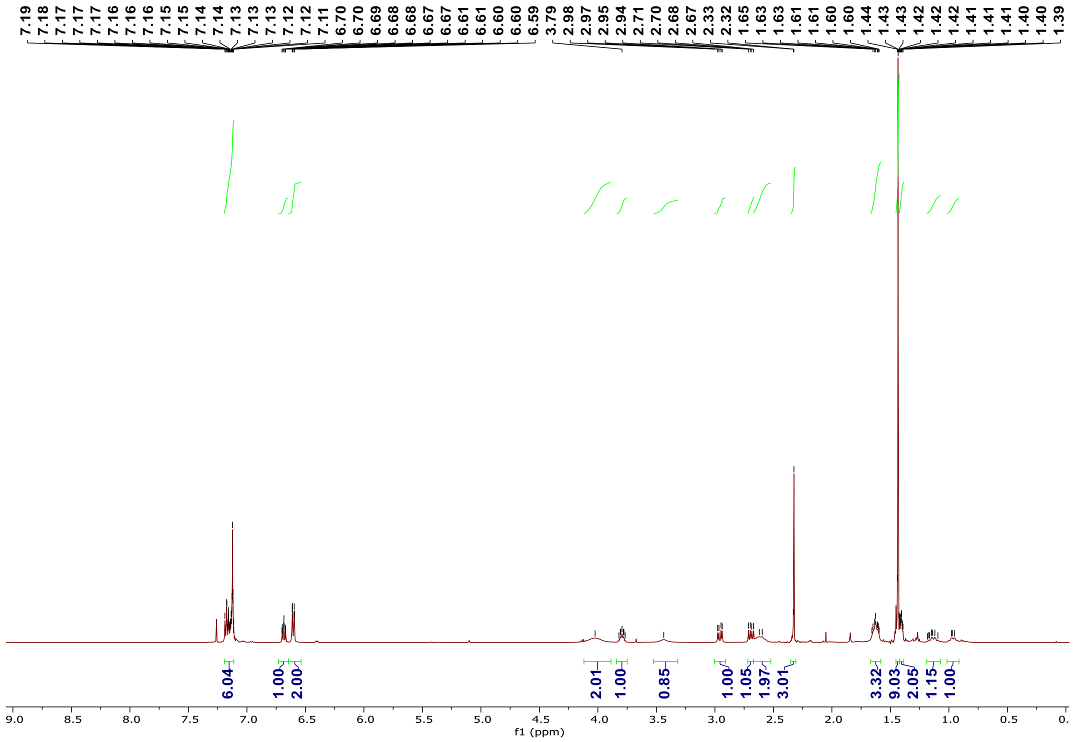
7.19 7.18 7.17 7.17 7.17 7.16 7.16 7.15 7.15 7.14 7.13 7.13 7.13 7.12 7.12 7.11 6.70 6.70 6.69 6.68 6.68 6.67 6.61 6.61 6.60 6.59 3.79 2.98 2.97 2.95 2.94 2.71 2.70 2.68 2.67 2.33 2.32 1.65 1.63 1.63 1.61 1.61 1.60 1.60 1.44 1.43 1.43 1.42 1.42 1.42 1.41 1.41 1.41 1.40 1.39
6.04
1.00
2.00
2.01
1.00
0.85
1.00
1.05
1.97
3.01
3.32
9.03
2.05
1.15
1.00
9.0 8.5 8.0 7.5 7.0 6.5 6.0 5.5 5.0 4.5 4.0 3.5 3.0 2.5 2.0 1.5 1.0 0.5 0.
f1 (ppm)

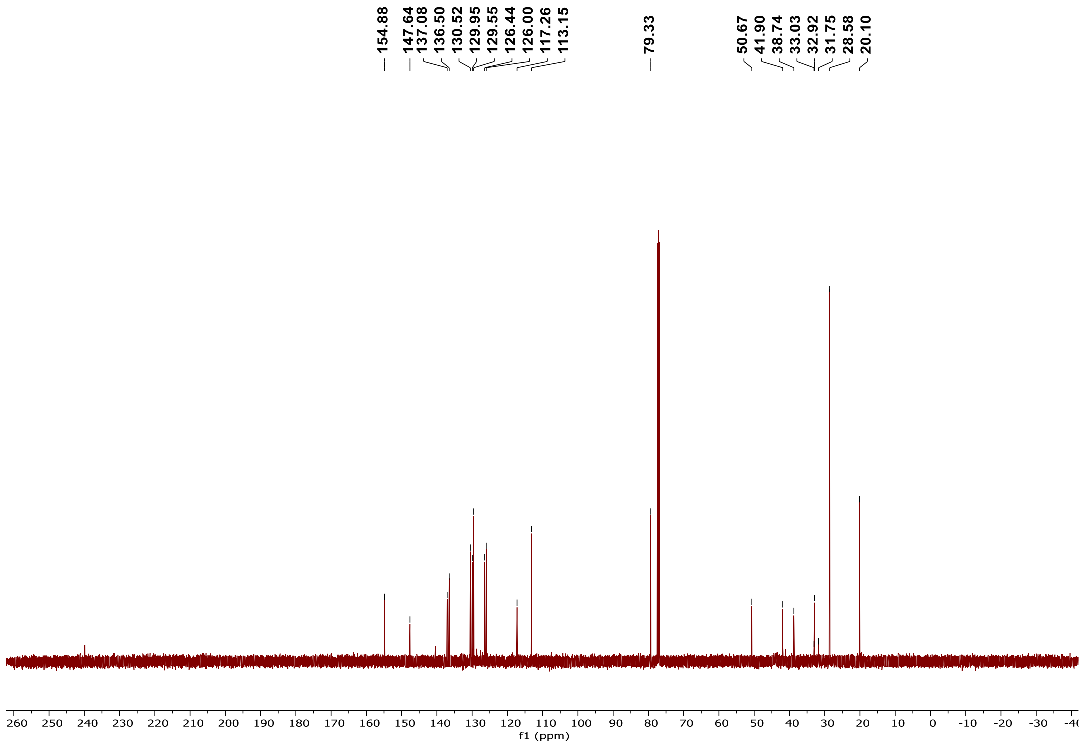
154.88
147.64
137.08
136.50
130.52
129.95
129.55
126.44
126.00
117.26
113.15
79.33
50.67
41.90
38.74
33.03
32.92
31.75
28.58
20.10
260 250 240 230 220 210 200 190 180 170 160 150 140 130 120 110 100 90 80 70 60 50 40 30 20 10 0 -10 -20 -30 -40
f1 (ppm)

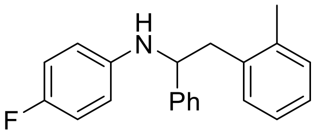
H
N
F
Ph

3gn

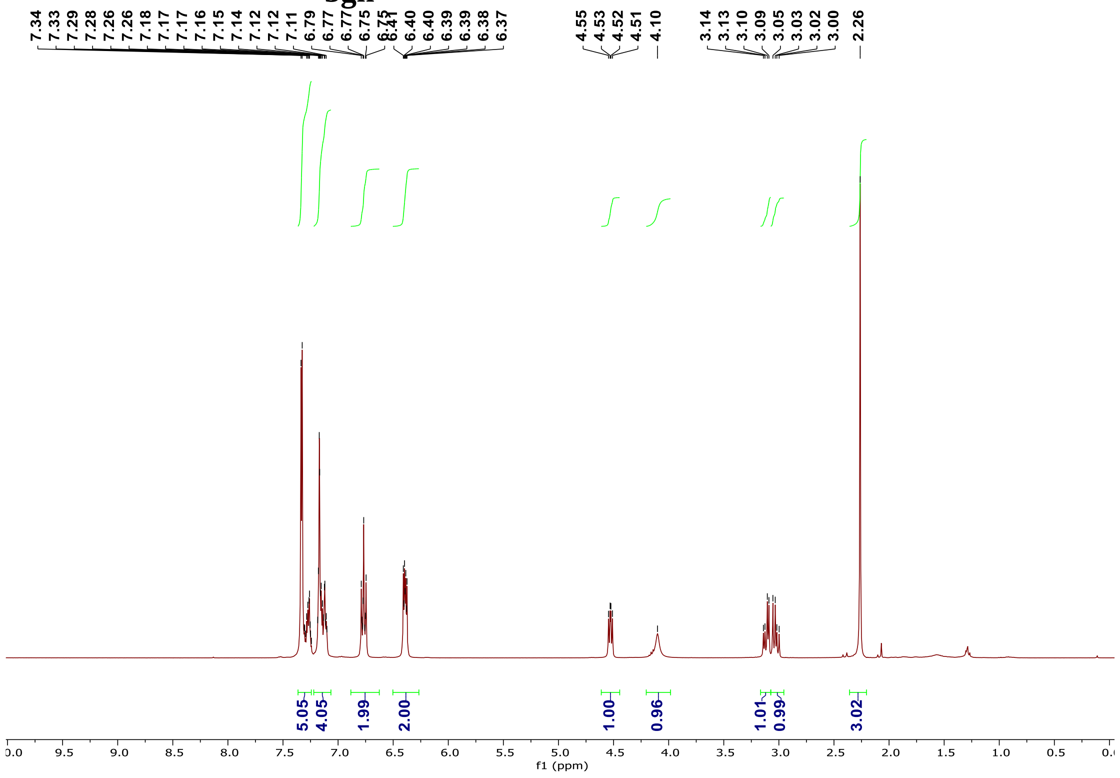
7.34
7.33
7.29
7.28
7.26
7.26
7.18
7.17
7.17
7.16
7.15
7.14
7.12
7.12
7.11
6.79
6.77
6.77
6.75
6.41
6.40
6.40
6.39
6.39
6.38
6.37
4.55
4.53
4.52
4.51
4.10
3.14
3.13
3.10
3.09
3.05
3.03
3.02
3.00
2.26
5.05
4.05
1.99
2.00
1.00
0.96
1.01
0.99
3.02
f1 (ppm)

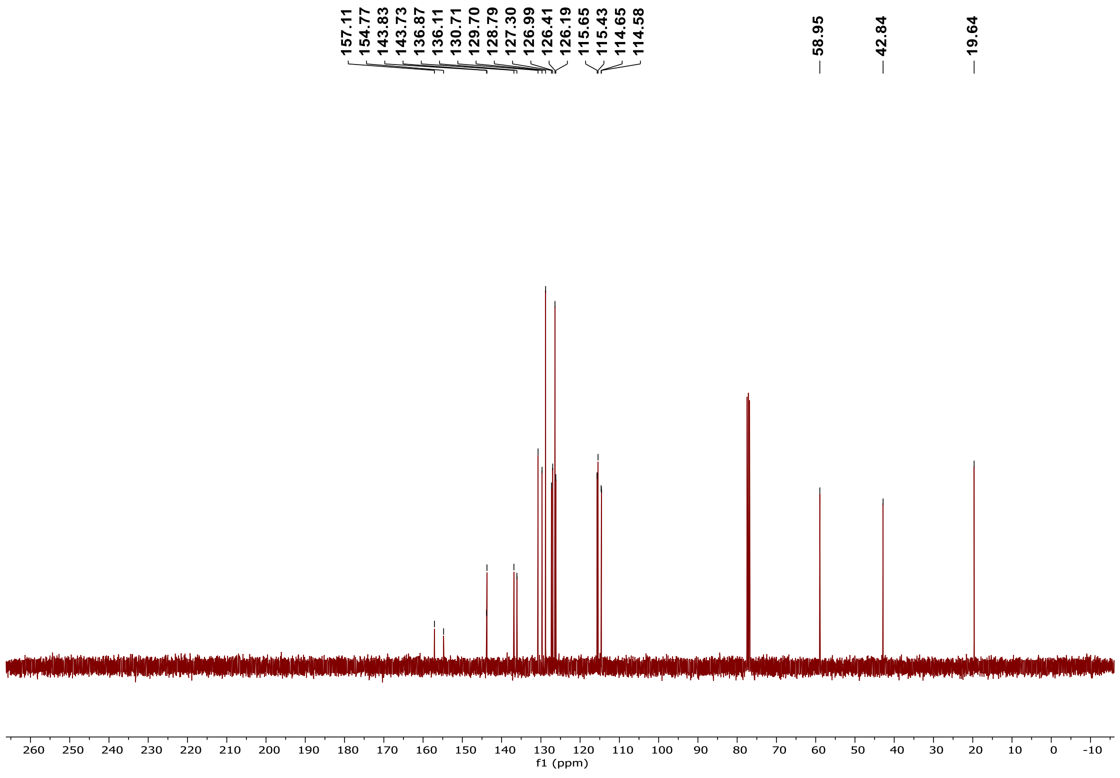
157.11
154.77
143.83
143.73
136.87
136.11
130.71
129.70
128.79
127.30
126.99
126.41
126.19
115.65
115.43
114.65
114.58
58.95
42.84
19.64
f1 (ppm)

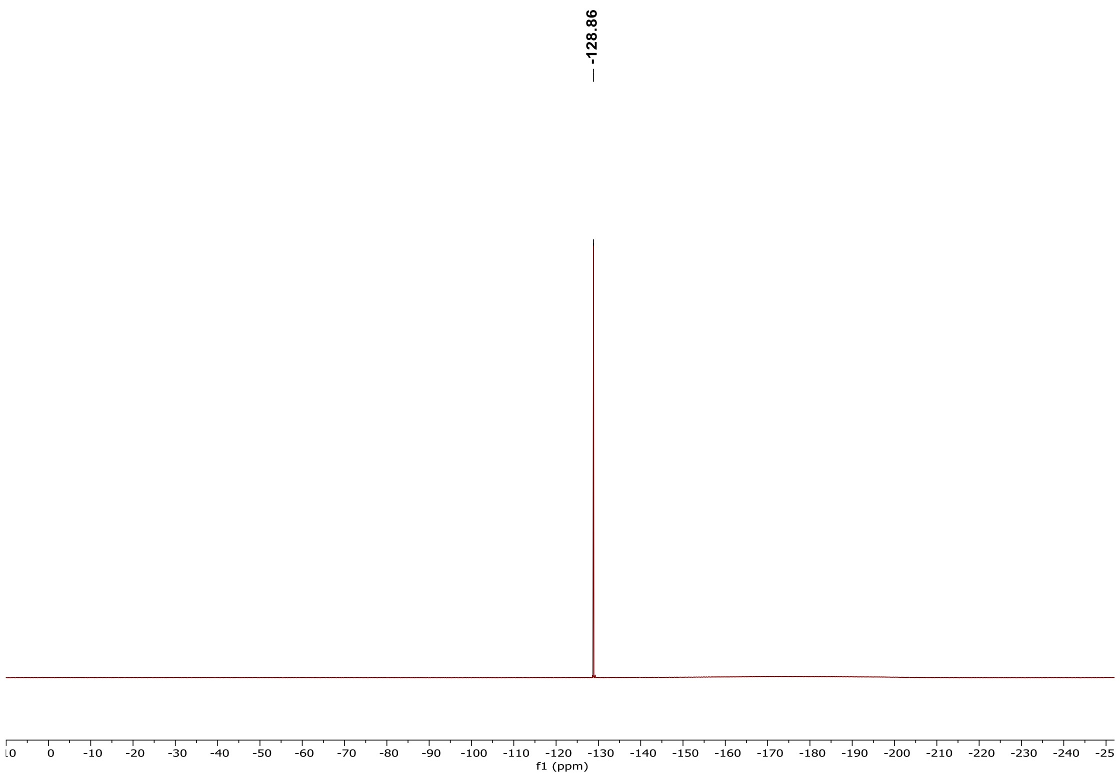
-128.86
10
0
-10
-20
-30
-40
-50
-60
-70
-80
-90
-100
-110
-120
-130
-140
-150
-160
-170
-180
-190
-200
-210
-220
-230
-240
-25
f1 (ppm)

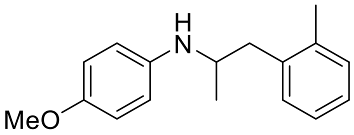

3hn

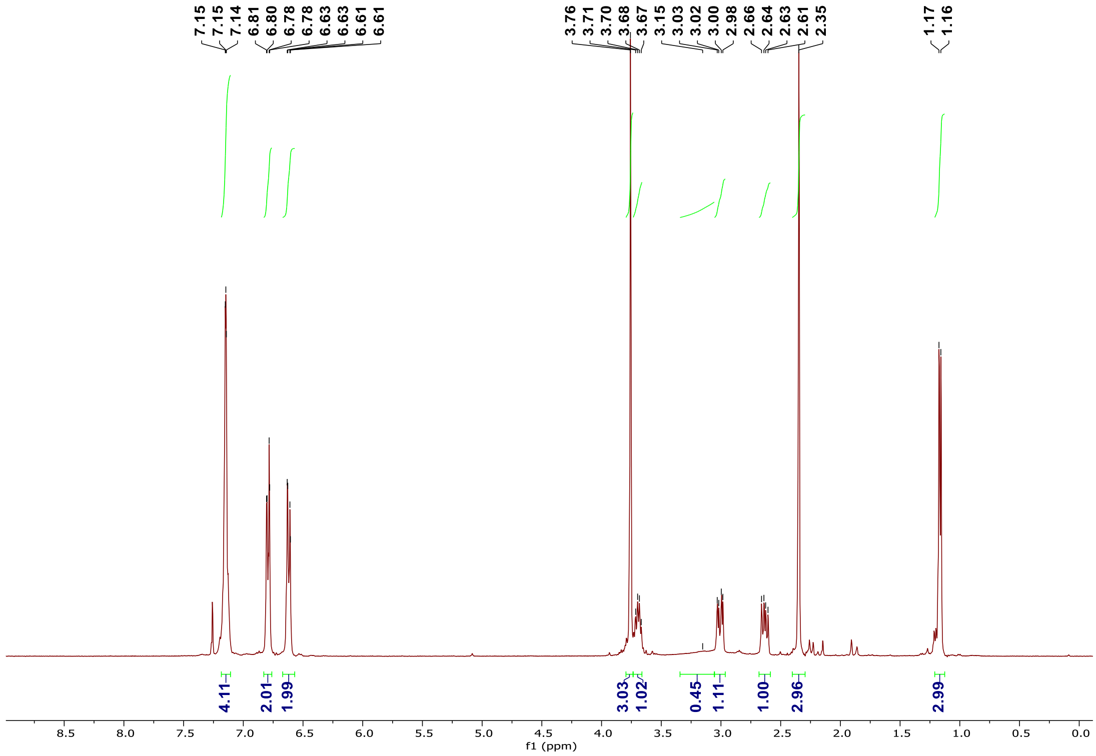

7.15
7.15
7.14
6.81
6.80
6.78
6.78
6.63
6.63
6.61
6.61
3.76
3.71
3.70
3.68
3.67
3.15
3.03
3.02
3.00
2.98
2.66
2.64
2.63
2.61
2.35
1.17
1.16
4.11
2.01
1.99
3.03
1.02
0.45
1.11
1.00
2.96
2.99
f1 (ppm)

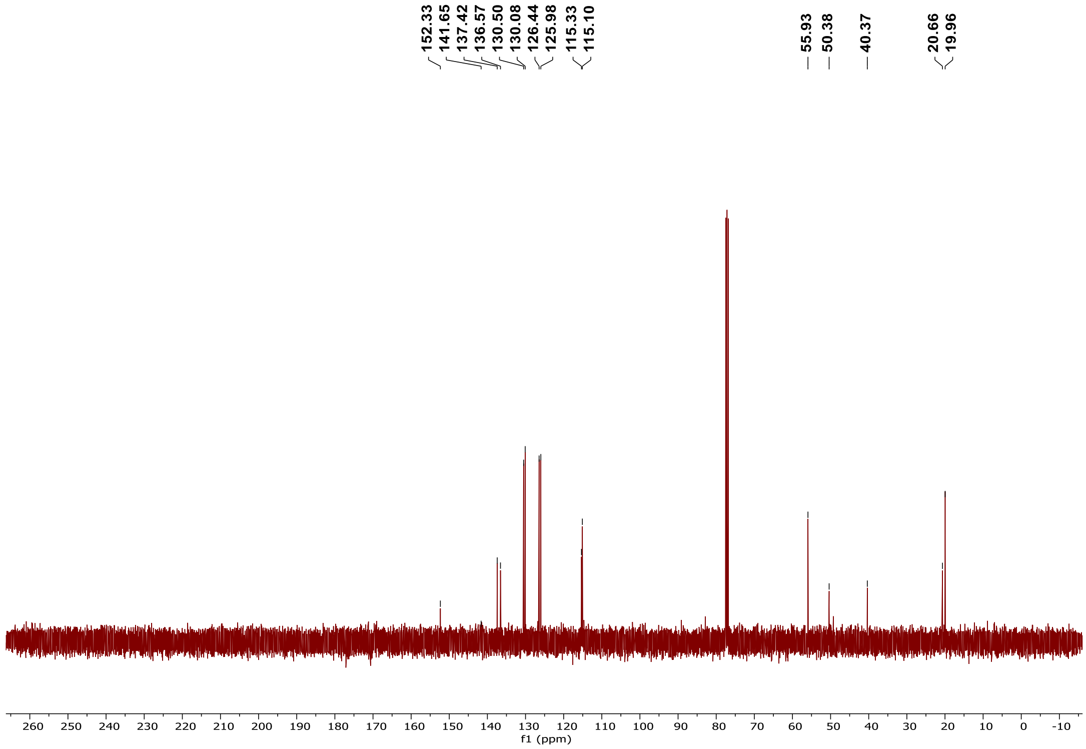

152.33
141.65
137.42
136.57
130.50
130.08
126.44
125.98
115.33
115.10
55.93
50.38
40.37
20.66
19.96
f1 (ppm)

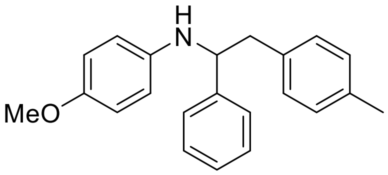

3ic

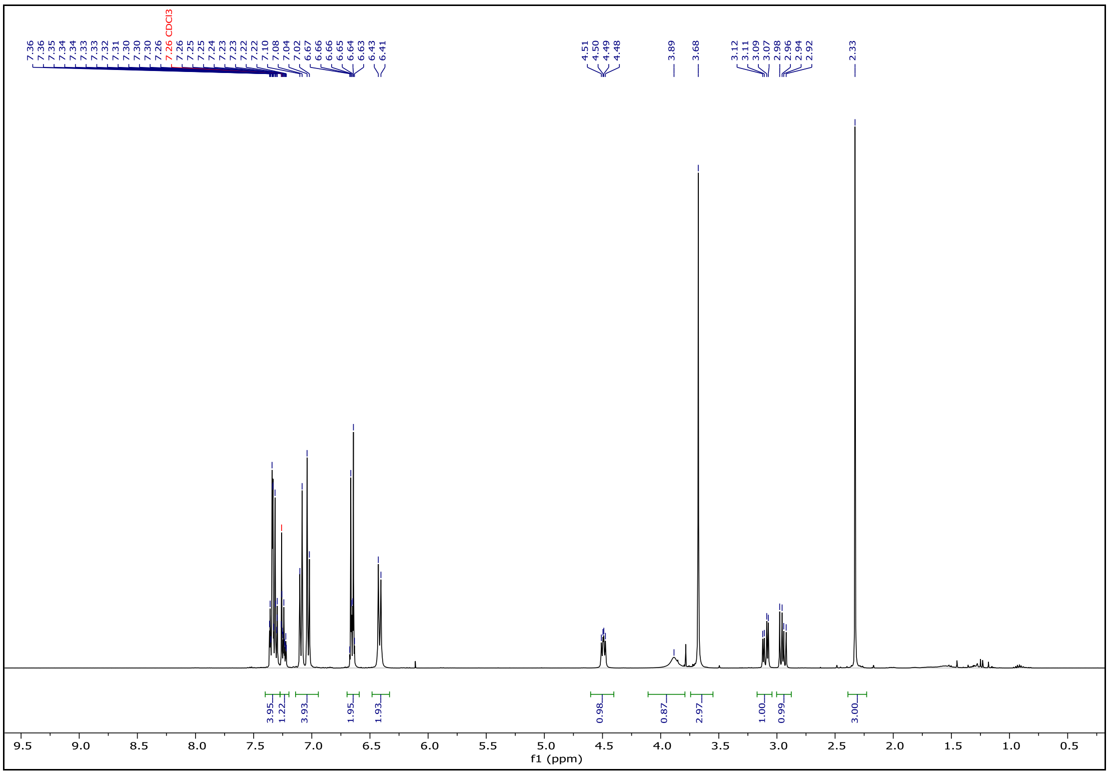

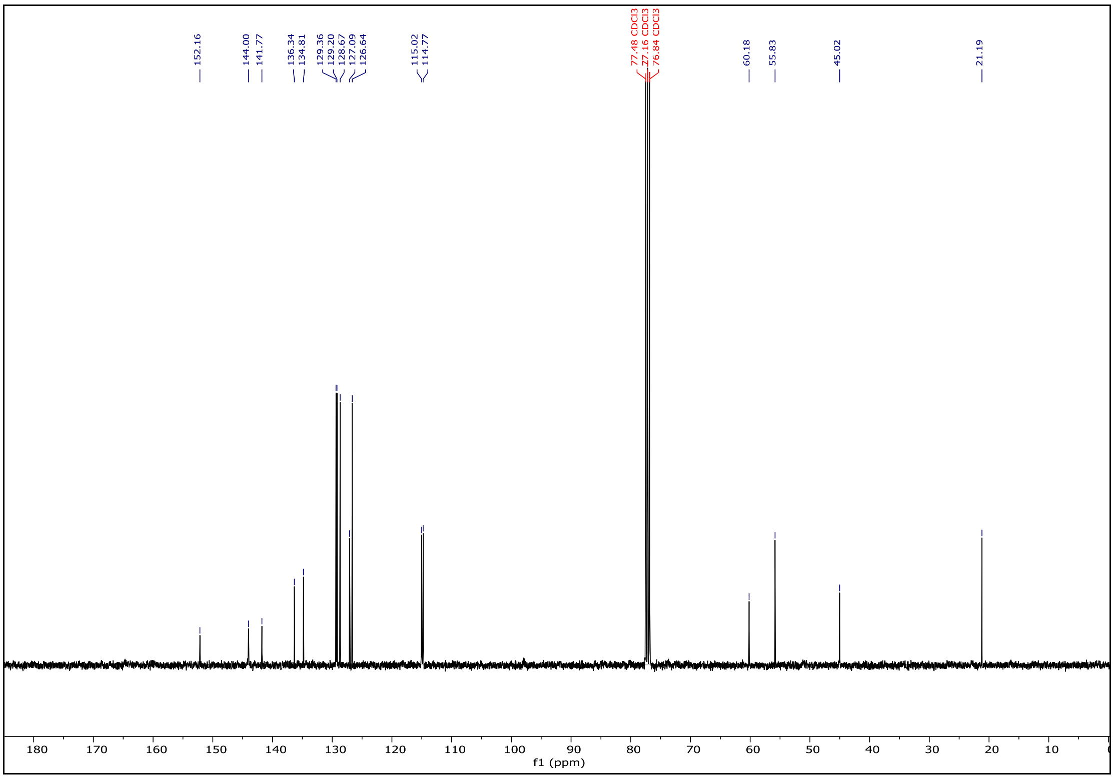

10. References

[1] H. Li, G. A. Grasa, T. J. Colacot, *Org. Lett.* **2010**, *12*, 3332-3335.

[2] a) T. Yasukawa, H. Nakajima, R. Masuda, Y. Yamashita, S. Kobayashi, *J. Org. Chem.* **2022**, *87*, 13750-13756; b) D. M. Mercea, M. G. Howlett, A. D. Piascik, D. J. Scott, A. Steven, A. E. Ashley, M. J. Fuchter, *Chem. Commun.* **2019**, *55*, 7077-7080.

[3] L. E. Longobardi, T. Mahdi, D. W. Stephan, *Dalton Trans.* **2015**, *44*, 7114-7117.

[4] J. S. M. Samec, J.-E. Bäckvall, *Chem. Eur. J.* **2002**, *8*, 2955-2961.

[5] Q. Lei, Y. Wei, D. Talwar, C. Wang, D. Xue, J. Xiao, *Chem. Eur. J.* **2013**, *19*, 4021-4029

[6] a) K. R. Reddy, E. Lubian, M. P. Pavan, H.-J. Kim, E. Yang, D. Holten, J. S. Lindsey, *New J. Chem.* **2013**, *37*, 1157-1173; b) D. P. Walker, B. A. Acker, E. J. Jacobsen, D. G. Wishka, *J. Heterocyclic Chem.* **2008**, *45*, 247-257.

[7] C. Weindl, S. L. Helmbrecht, L. Hintermann, *J. Org. Chem.* **2023**, *88*, 4155-4161.

[8] N. Chen, X.-J. Dai, H. Wang, C.-J. Li, *Angew. Chem. Int. Ed.* **2017**, *56*, 6260-6263.

[9] L. Leng, J. M. Ready, *ACS Catal.* **2020**, *10*, 13196-13201.

[10] Y.-Z. Wang, S.-D. Liu, L. Cheng, L. Liu, C.-J. Li, *Org. Chem. Front.* **2023**, *10*, 3021-3026.

[11] E. L. Gall, C. Haurena, S. Sengmany, T. Martens, M. Troupel, *J. Org. Chem.* **2009**, *74*, 7970-7973.

[12] K.-H. Kim, C.-Y. Lee, C.-H. Cheon, *J. Org. Chem.* **2015**, *80*, 6367-6374.

[13] X. Xie, T. Y. Zhang, Z. Zhang, *J. Org. Chem.* **2006**, *71*, 6522-6529.

[14] C. Weindl, S. L. Helmbrecht, L. Hintermann, *J. Org. Chem.* **2023**, *88*, 4155-4161.

[15] L. Ackermann, *Org. Lett.* **2005**, *7*, 3123-3125.

[16] G. Wicker, R. Zhou, R. Schoch, J. Paradies, *Angew. Chem. Int. Ed.* **2022**, *61*, e202204378.
